# The effects of self-efficacy enhancing program on foot self-care behaviour of older adults with diabetes: A randomised controlled trial in elderly care facility, Peninsular Malaysia

**DOI:** 10.1371/journal.pone.0192417

**Published:** 2018-03-13

**Authors:** Siti Khuzaimah Ahmad Sharoni, Hejar Abdul Rahman, Halimatus Sakdiah Minhat, Sazlina Shariff-Ghazali, Mohd Hanafi Azman Ong

**Affiliations:** 1 Centre for Nursing Studies, Faculty of Health Sciences, Universiti Teknologi MARA, Puncak Alam Campus, Puncak Alam, Selangor, Malaysia; 2 Department of Community Health, Faculty of Medicine and Health Sciences, Universiti Putra Malaysia, UPM Serdang, Selangor, Malaysia; 3 Department of Family Medicine, Faculty of Medicine and Health Sciences, Universiti Putra Malaysia, UPM Serdang, Selangor, Malaysia; 4 Department of Statistics, Faculty of Computer Science and Mathematics, Universiti Teknologi MARA, Segamat Campus, Segamat, Johor, Malaysia; TNO, NETHERLANDS

## Abstract

**Background:**

Self-care behaviour is essential in preventing diabetes foot problems. This study aimed to evaluate the effectiveness of health education programs based on the self-efficacy theory on foot self-care behaviour for older adults with diabetes.

**Methods:**

A randomised controlled trial was conducted for 12 weeks among older adults with diabetes in elderly care facility in Peninsular Malaysia. Six elderly care facility were randomly allocated by an independent person into two groups (intervention and control). The intervention group (three elderly care facility) received a health education program on foot self-care behaviour while the control group (three elderly care facility) received standard care. Participants were assessed at baseline, and at week-4 and week-12 follow-ups. The primary outcome was foot-self-care behaviour. Foot care self-efficacy (efficacy expectation), foot care outcome expectation, knowledge of foot care and quality of life were the secondary outcomes. Data were analysed with Mixed Design Analysis of Variance using the Statistical Package for the Social Sciences version 22.0.

**Results:**

184 respondents were recruited but only 76 met the selection criteria and were included in the analysis. Foot self-care behaviour, foot care self-efficacy (efficacy expectation), foot care outcome expectation and knowledge of foot care improved in the intervention group compared to the control group (p < 0.05). However, some of these improvements did not significantly differ compared to the control group for QoL physical symptoms and QoL psychosocial functioning (p > 0.05).

**Conclusion:**

The self-efficacy enhancing program improved foot self-care behaviour with respect to the delivered program. It is expected that in the future, the self-efficacy theory can be incorporated into diabetes education to enhance foot self-care behaviour for elderly with diabetes living in other institutional care facilities.

**Trial registration:**

Australian New Zealand Clinical Trial Registry ACTRN12616000210471

## Introduction

More than 134.6 million older adults worldwide have diabetes, and the number is projected to increase beyond 252.8 million by 2035 [1]. The rate of diabetes complication is high in many countries; 27.2% had macrovascular and 53.5% had microvascular complications [2]. Diabetes management requires major changes in behaviour [3]. It includes knowledge, skills and confidence to make improvements in self-care behaviour and deal with associated psychological aspects [4]. Foot care is part of standard practice guidelines in diabetes self-care behaviour [1, 5–7]. Older diabetics need to perform foot self-care behaviour regularly to prevent and delay potential complications [6].

Several factors in the older population may influence their self-care behaviour including physical limitation, health status, as well as cognitive and psychosocial aspects [8–9]. Self-efficacy is defined as “individuals’ beliefs about their capabilities to produce designated levels of performance in any activity that has influence over events that affect their lives” [10]. It focuses on beliefs about the abilities of a person to perform a specific action [11–13]. Self-efficacy can be increased by providing clear instructions, skills or training, and demonstrating the desired behaviour [14]. This concept has been increasingly applied as a model of health behaviour and as a framework for developing health intervention programs in various populations [15, 16]. Previous studies showed positive improvements in foot self-care behaviour among individuals with diabetes after incorporating the self-efficacy concept in their intervention program [17–19]. However, due to different methodological approaches and outcomes, the results might not be generalised to other populations.

A systematic review reported that there were positive effects of foot care education program on patient’s knowledge and foot care behaviour [20]. However, in their review, only one of the 11 randomised controlled trials (RCT) demonstrated a low risk of bias. Besides, a more recent systematic review was unable to support whether foot ulcers among adults with diabetes can be healed by controlling blood glucose levels [21]. Hence, there is a need for more RCTs with a robust methodology.

In Malaysia, the National Health Morbidity Survey (2015) reported that 17.5% of the population have diabetes [22]. The rapid increase of the ageing population in Malaysia would translate to an estimated accelerated increase in the number of older adults with diabetes [23, 24]. There was a general increasing trend in diabetes prevalence with age; from 2.0% in the 18–19 years age group to a prevalence ranging between 20.8% to 26.2% among those aged 60–64 years [25]. The number of microvascular complication was 75.0% [26], and the prevalence of neuropathy, diabetic foot ulcer and amputation were 70.0%, 11.1% and 11.0%, respectively [27]. The National Orthopaedic Registry Malaysia (2009) reported that the highest prevalence of diabetic foot problems were among the older population (38.3%), those with primary/low education level (49.3%), retired (13.8%), and unemployed (11%) [28]. The highest risk factors of foot problems were diabetics involved in large amounts of walking/standing while performing activities at work (47.4%), wearing slippers (47.3%), and nearly half of them were barefoot at home (49.5%) [28]. Diabetes self-care behaviour was poor among older Malaysian adults [29] and they have low expectations in healthcare [30].

The ageing population in Malaysia is becoming a challenge for healthcare workers. The older adults require adequate care and facilities for a better quality of life and healthy ageing [31]. Lack of family members can be a major missing element in the support system [32]. Some stayed in elderly care facility or day care centres. Approximately 9.0% of older Malaysian adults living in public elderly care facility have diabetes and 32.0% were on three or more medications [33]. The author found that the older adults in care centres have a moderate level of well-being [34]. Diabetes care activities such as blood glucose monitoring and medication intake in public elderly care facility in Malaysia were managed by the local healthcare staff. However, the efficacy and influence of a health education program on their foot self-care behaviour is still largely unexplored in Malaysia. Thus, this study proposes to contribute new data pertaining to the older population living in elderly care facility in Malaysia. The objective of this study was to evaluate the effectiveness of health education programs based on the self-efficacy theory on foot self-care behaviour for older adults with diabetes.

## Materials and methods

### Design and setting of the study

A randomised controlled trial was conducted over 12 weeks at six public elderly care facility in Peninsular Malaysia, Malaysia. Data was collected between February and July 2016 (Table 1). These elderly care facility are called Rumah Seri Kenangan (RSK), under the Ministry of Women, Family and Community Development. Currently, there are eight public elderly care facility in Peninsular Malaysia; seven in the Western region, and one in the Eastern region. There are approximately 1,496 older adults staying in the seven public elderly care facility, in the Western region [35]. All elderly care facility in the Western region were selected; one for piloting and the other six for this trial. The number of older adults with diabetes is about 20–40 in each elderly care facility.

**Table 1:** The effects of self-efficacy enhancing program on foot self-care behaviour of older adults with diabetes: A randomised controlled trial in elderly care facility, Peninsular Malaysia.

**Date**	**Procedures (recruitment)**	**Material/ instrument**	**Time**	**Location**	**Provider**
Week-0Feb/ March 2016	Day-1: explanation about the study (brief) and screening process	Manual file, screening tool (Katz Index, M-ECAQ, M-GDS)	20 mins	RSK	Researcher
	Explanation about the study (detail) and consent taking	Manual file, subject info sheet and consent form	10 mins		
					Research assistants
	Day-2: baseline data	Manual file, questionnaire	30 mins		
	**Activities (random allocation procedures)**	30 mins			
				
**THE INTERVENTION GROUP**					
**Date**	**Procedures the self-efficacy enhancing program**	**Material/ instrument**	**Time**	**Location**	**Provider**
Week-0March/ April 2016	✓ Day-1: a group seminar presentation on foot care	Manual file, laptop, screen projector, PPT and foot kit	30 mins	RSK clinic	Researcher
	**Physical & emo. states:** the participants are physically and emotionally stable to involve in the program				
	Knowledge transfer: oral (PPT) and written (pamphlet) information				
	Tailored action plan: establish an agreement on new goals together				
	**Performance accomplishment:** advise start to work with small realistic steps, to the new behaviour				
	**Verbal persuasion:** build up rapport and thrust-worthiness, give guidance and encouragement				
	**Vicarious experience:** advice to read and refer to the pamphlet (symbolic modelling)				
	Leave responsibility and encourage an active role (independent) of the participants				
	Briefing to the local nurse: to make a serial visit to the participants	Manual file, checklist reminder and pamphlet	30 mins		
Week-1Week-2 & Week-3	✓ The researcher make a phone call to the local nurse for weekly visits	Telephone and checklist reminder	5 mins	RSK	Researcher & localhealth care provider
	Remind the participants of foot self-care behaviour				
	Verbal persuasion: give positive feedback and encouragement				
	Vicarious experience: advice to read and refer to the pamphlet (symbolic modelling)				
Week-4April/ May 2016	Day-1: data collection	Manual file, questionnaire	20 mins	RSK	Research assistants
	✓ Follow-up: one-to-one discussion	Manual file, foot kit and checklist reminder	20 mins		Researcher
	Self-evaluation: get a feedback on goals, determine of obstacles, if any				
	**Physical & emo. states:** give attention to participants who have difficult situations (pain, illness, stress)				
	**Performance accomplishment:** continue to practice the desired behaviour successfully				
	**Vicarious experience:** share experiences each other (social modelling)				
	**Vicarious experience:** advice to read and refer to the pamphlet (symbolic modelling)				
	**Verbal persuasion:** give encouragement, advise and specific guidance				
Week-6, Week-8 & Week-10	✓ The researcher make a phone call to the local nurse for biweekly visits	Telephone and checklist reminder	5 mins	RSK	Researcher & localhealth care provider
	Remind the participants of foot self-care behaviour				
	Verbal persuasion: give positive feedback and encouragement				
	Vicarious experience: advice to read and refer to the pamphlet (symbolic modelling)				
Week-12June/ July 2016	Day-1: data collection	Manual file, questionnaire	20 mins	RSK	Research assistants
	Evaluation: one-to-one discussion (repeat the activities as conducted at week-4, if required	Manual file, foot kit and checklist reminder	20 mins		Researcher
	Advise the participants to keep continue with the positive foot self-care behaviour				
**THE CONTROL GROUP**					
**Date**	**Procedures the standard care**	**Material/ instrument**	**Time**	**Location**	**Provider**
Week-0March/ April 2016	Usual health care			RSK	Local healthcare provider
Week-1Week-2 & Week-3	Usual health care			RSK	Local healthcare provider
Week-4April/ May 2016	Day-1: data collection	Manual file, questionnaire	20 mins	RSK	Research assistants
	Usual health care			RSK	Local healthcare provider
Week-6, Week-8 & Week-10	Usual health care			RSK	Local healthcare provider
Week-12June/ July 2016	Day-1: data collection	Manual file, questionnaire	20 mins	RSK	Research assistants
	✓ A group seminar presentation on foot care	Manual file, laptop, screen projector, PPT and foot kit	30 mins	RSK clinic	Researcher
	**Physical & emo. states:** the participants are physically and emotionally stable to involve in the program				
	Knowledge transfer: oral (PPT) and written (pamphlet) information				
	Tailored action plan: establish an agreement on new goals together				
	**Performance accomplishment:** advise start to work with small realistic steps, to the new behaviour				
	**Verbal persuasion:** build up rapport and thrust-worthiness, give guidance and encouragement				
	**Vicarious experience:** advice to read and refer to the pamphlet (symbolic modelling)				
	Leave responsibility and encourage an active role (independent) of the participants				
	Advise the participants to keep continue with the positive foot self-care behaviour				

The Consolidated Standard of Reporting Trials (CONSORT) flow diagram was used for the outline of the design [36]. Methodological assessment and design guideline of this study was adopted from the CONSORT statement for assessing non-pharmacologic treatments checklist [37, 38].

### Characteristics of participants

In this trial, the inclusion criteria for participation were: Malaysian, aged 60 years or more, have been diagnosed with diabetes by medical doctor, presented with or without diabetic foot problems, the ability to communicate sufficiently (in Malay) to understand the education program, the ability to perform daily activities independently (e.g., bathing, feeding, grooming etc.), and have no major complications, which would interfere with the program (e.g., blind, mute, deaf, or bed-ridden).

The main researcher (S. K. A. S) performed the process of screening for functional status, cognitive function and depressive symptoms. Potential participants were assessed for functional status using the Katz Index of Independence in Activities of Daily Living (ADLs) [39], and those who scored ≥ 4 were included in this study. The information of functional status assessment was determined by direct observation and/ or from personal health record.

Participants diagnosed with cognitive function impairment and/or with depressive symptoms and/or with mental health conditions and presenting psychotic symptoms were excluded from the study. Cognitive function and depressive symptoms were assessed via personal interview using the Malay version of the Elderly Cognitive Assessment Questionnaire (M-ECAQ) [40], and the Malay Geriatric Depression Scale (M-GDS) [40]. Scores ≤ 4 for the M-ECAQ and ≥ 2 for the M-GDS indicated cognitive impairment and the presence of depressive symptoms, respectively [40].

The sample size estimation was carried out using the hypothesis testing of two population means formula [41]. The sample size considered the standard errors associated with confidence intervals (95% = 1.96) (2-tailed) and power (80% = 0.842) [41]. Based on a previous study, the mean difference in the foot self-care score (baseline: 32.32±6.76) and post-intervention: 36.22±6.95) were used [42]. The calculated sample size was adjusted for design effect; assuming a cluster size of 6 and intracluster correlation of 0.05. This means 59 of participants are needed for each group to participate in this study. However, an additional number of participants was required to retain 20% of potential attrition. Therefore, a minimum of 71 is considered an adequate number to recruit for a dropout rate and potential of attrition. Thus, a total of 142 eligible respondents were needed in order to retain 118 respondents (59 respondents per group) at the end of the study.

### Recruitment and screening

Participants who meet the inclusion criteria and agreed to participate were invited to join the program. A screening process was conducted to determine the eligibility and to minimise selection bias that may influence the effectiveness of the program. Potential participants were assessed one-to-one by the main researcher for functional status, cognitive function and depression level.

### Randomisation and blinding

Randomisation at individual level was not conducted. Simple randomisation was conducted with a cluster size of “6” at centre level (i.e. elderly care facility). After baseline assessment, six elderly care facility were allocated randomly (sequentially numbered in a sealed envelope) by a lecturer who was not involved in this study. Six numbers were generated using the random sequence number generator where each group had an equal chance to be either in the intervention group or control group. The odd numbers (1, 3 and 5) were taken as the intervention group (RSK Johor Bharu, RSK Taiping and RSK Kangar) and the even numbers (2, 4 and 6) were placed under the control group (RSK Ulu Kinta, RSK Cheng and RSK Bedong).

In this single blinded study, only the researcher (S.K.A.S) knew the group allocation (intervention or control). Individuals assessing the outcomes (e.g. research assistants), analysing the data (e.g. statistician) or others who were involved in this study (e.g. administration staff or local healthcare provider) were unaware of the group assignment. Participants in the intervention group were only aware during the first day of the intervention program.

### Description of materials, processes, interventions and comparisons

The Template for Intervention Description and Replication (TIDieR) checklist guidelines [43] was used to describe the intervention components. The intervention group received the health education program while the control group received the standard care. Standard care was defined as a routine or usual healthcare service for diabetes patients received from the local healthcare provider in the elderly care facility. After completion of the study, the control group received the same program as the intervention group.

#### Elements of theory in the intervention

The theoretical background of the program was based on Albert Bandura’s Self-efficacy theory with the emphasis on taking action to promote self-efficacy [18, 44]. It included components for enhancing self-efficacy level such as performance accomplishment, vicarious experience, physical and emotional states, and verbal persuasion [13, 14]. Self-efficacy enhancing activities were applied together with knowledge transfer during the intervention program.

#### Materials and procedures

In this study, the materials that were used consisted of a questionnaire, a Power Point presentation (PPT) and a pamphlet (for participants), a checklist reminder (for the local healthcare provider), and a manual file (for the researcher and research assistant). In this study, questionnaires were used for the screening process and measuring research outcomes. As a measure to improve participant retention, a foot kit (containing a pamphlet on foot care, nail-clipper, moisturising lotion, small towel) and goodies were given to the participants.

The information distributed included awareness of risk factors and its complications, hygiene and inspection, skin and nail care, appropriate footwear, injury prevention, and when to seek a healthcare professional. The knowledge was given to participants, local healthcare providers, and research assistants.

The procedures involved a total of four fieldwork visits, screening and baseline assessment, the intervention/ health education program was conducted within one month after baseline assessment, with follow ups at week-4 and week-12.

During each follow-up, experience sharing, feedback on goals as well as an assessment of obstacles and problematic situations were conducted by the main researcher. Routine visits to participants were conducted biweekly (to monitor adherence) until the end of the program (week-12).

#### Intervention provider

The health education program was given by the main researcher (registered nurse). Two research assistants (registered nurses) conducted the data collection at baseline, week-4, and week-12. They received specific training with a manual file and were trained to carefully observe how the main researcher conducted the data collection process during the pilot study. In this trial, research assistants collected the data (on the same day and at same location).

Routine visits were made by a healthcare provider (who is in-charge of the elderly care facility) to give continuous support to the participants and they were guided by the checklist. The local healthcare provider received a 30-minute briefing by the main researcher on how to remind and advise the participants on using the checklist.

#### Location, modes of delivery, duration of intervention

The program was conducted either at a meeting room or the clinic of each elderly care. During the intervention program, a group seminar (the intervention/health education program) was delivered to the intervention group, consisting of a 20-30-minute Power Point presentation (8–10 participants/ group/ session). A 20-minute one-to-one discussion was conducted during the follow-ups.

#### Tailoring, modifications, and intervention adherence

The content of the health education program was adapted from international and local sources [1, 5–7, 25, 27]. The intervention program was culturally tailored for Malaysian older adults with diabetes living in elderly care facility. An interview was conducted with a diabetes nurse educator, an endocrinologist, a family medicine specialist, and an older adult with diabetes to obtain information about the most common problems they experienced in following the recommended diabetes foot self-care behaviour. As most the participants were older adults and have a low socio-economic background, the structure and content of the education program had been simplified to increase the understanding and adherence of participants.

The questionnaire (Malay version) underwent validity checks and reliability tests. A panel of six experts judged the content of the questionnaires using the Content Validity Ratio (CVR). The formula for CVR = (2ng / N)– 1, was used for validity conformity [45]. A CVR result of < 0.00 were excluded in any items of the questionnaire, meaning less than 50% of the experts in a panel size of N believe that the item was essential and valid [46].

A pilot study was conducted in RSK Cheras, Selangor, Malaysia prior to the conduct of the main study. Thirty-one older adults were assessed for their willingness to answer the questionnaire (Malay version), adequacy of time and to observe any ambiguous words and instructions. The results of the internal consistency test (Cronbach's Alpha) for foot self-care behaviour, foot care self-efficacy (efficacy expectation), foot care outcome expectation, knowledge of foot care and quality of life (physical symptoms) and (psychosocial functioning) were 0.68, 0.91, 0.88, 0.86, 0.68 and 0.68 respectively [47]. The results of the test-retest reliability for foot self-care behaviour (r_s_ = 0.69), foot care self-efficacy (efficacy expectation) (r_s_ = 0.73), foot care outcome expectation (r_s_ = 0.69), knowledge of foot care (r_s_ = 0.67) and quality of life (physical symptoms) (r_s_ = 0.69) and (psychosocial functioning) (r_s_ = 0.67) were significant (p < 0.05). Minor modifications on the education program and the questionnaire were made based on the pilot study results. The revised education program and questionnaire were not re-tested.

The fidelity of the program was carefully assessed and evaluated by the research team, the research ethics committee of the university, and the Social Welfare Department. Research activities were recorded in a log book by the researcher. A series of meetings with the research team was conducted to ensure that the program had been implemented as designed. Every six months, a research progress report was submitted to the university. A full report of the study results was required to be submitted to the Social Welfare Department after completion of the study. No amendments were made to the study protocol except for the education program and the questionnaire.

### Outcome assessment

The primary outcome of this study was foot-self-care behaviour. Foot care self-efficacy (efficacy expectation), foot care outcome expectation, knowledge of foot care and quality of life were the secondary outcomes. The information was collected through face to face interviews at baseline, week-4 and week-12 after the intervention program.

The demographic data (age, gender, ethnicity, education level, marital status, having children, and duration of stay in the elderly care facility) and clinical characteristics (fasting blood glucose, duration of diabetes, treatment of diabetes, other disease except diabetes, smoking status, previous diabetes education received and hospitalisation due to diabetes problem) were collected as baseline data.

#### Foot self-care behaviour (FSCB)

The scale was developed based on the modified version of Diabetes Foot Self-Care Behaviour Scale (DFSBS) [47] [48] and previous literature [1, 5, 7, 24, 27]. The original DFSBS has good validity and reliability (Cronbach’s alpha = 0.73), and the test retest reliability was 0.92 [47] [48]. In this study, the scale contained 16 items and were rated on a 5-point Likert-type scale with the higher scores representing better foot self-care behaviours. The total score ranged from 16–80.

Foot self-care behaviour consisted of two sections. In section-1, seven items were asked about how many days the respondents had performed the foot self-care behaviour in the past seven days (one week). Section-2 (nine items) was about the frequency in which respondents performed certain foot self-care behaviour. The responses were rated as a 5-point Likert scale [never/ 0 day per week (1), rarely/ 1–2 days per week (2), sometimes/ 3–4 days per week (3), often/ 5–6 days per week (4) and always/ 7 days per week (5)] (Chin & Huang, 2013). The score ranged from 16 to 80; a higher score indicated good foot self-care behaviour.

#### Foot care self-efficacy (efficacy expectation) (FCSE)

The FCSE was developed based on the modified version of Foot Care Confidence Scale (FCCS) [48] [49] and previous literature [1, 5, 7, 24, 27]. The Cronbach’s alpha of the original FCCS was high (0.92) [48] [49]. In this study, there were 10 items in the FCSE measuring self-confidence in managing foot care. The FCSE scale was based on five scores; strongly not confident (score 1) to strongly confident (5). Higher scores indicated higher self-confidence to perform foot care behaviour. The total score ranged from 10–50.

#### Foot care outcome expectation (FCOE)

The scale was developed based on previous literature [14, 48–51]. This scale measured the participant’s confidence that the desirable results can be achieved if they perform proper foot self-care behaviour. It had six items and the scale consisted of five scores; strongly disagree (1), to strongly agree (5). The score ranged from 6–30; a higher score indicated that the participant has a high self-confidence that the foot self-care behaviour he/ she performed will produce a good effect.

#### Knowledge of foot care (KOFC)

The scale was adapted based on previous studies [52–54]. The questions asked were related to diabetic foot complications, risk factors, and foot care behaviour. The scale consisted of 11 items with three possible answers (true, false, don’t know). Each correct answer was given 1 point. A higher score indicated a good level of knowledge of foot care. The total score ranged from 0–11.

#### Quality of life (QoL)

The QoL was measured using the modified version of the Neuropathy and Foot Ulcer Specific Quality of Life (FS-QoL) [55]. The original FS-QoL had been shown to demonstrate good reliability (alpha = 0.86–0.95) [55]. In this study, the participants were required to recall (in the past 4 weeks) their feelings, how foot issues (if any) affect their daily activities, relationships, and feelings. The instrument included items 1–13 (symptoms) and 14–25 (psychosocial functioning). In this study, the scale was divided into two sections. First, the participants needed to respond to foot problems affecting their well-being [always (3), sometimes (2), never (1)] and psychosocial functioning [every time (3), seldom (2), none (1) or agree (3), neither agree or disagree (2) and disagree (1)]. In the second section, the participants were asked about whether the foot problems bother them [none (1), some (2), or very (3)]. The score range for physical symptoms were from 13 to 117 and for psychosocial functioning was from 12 to 108. A lower score indicated a good quality of life.

#### Translation process for the questionnaire

In this study, the questionnaire was translated from English to Malay. Firstly, a bilingual translator, certified by the Institute of Language and Literature Malaysia translated the questionnaire for the forward translation process.

Secondly, the experts then reviewed the Malay version to identify and resolve the inadequate expressions/concepts of the translation, wordings and meanings of the instrument, as well as any discrepancies between the forward translation and the existing or comparable previous versions of the questions, if any. A third party (medical lecturer) was invited as an expert to give their opinion on the cultural equivalency of the questionnaire and the appropriateness of the language used in the items.

After that, another translator unaware of the original English instruments performed a backward translation of the Malay format into the English language. The backward translator is a native speaker of the English language and fluent in the language of translation. This technique is called semantic translation and the translators are individuals with a medical and health sciences background.

The experts then reviewed the recent English version of the questionnaire for conceptual equivalence with the original source, to identify any linguistic inaccuracies and to check for conceptual discrepancies. This was done to streamline the translations cross-culturally and contribute to the standardisation of the questionnaire. These processes aimed to ensure the equivalent meaning of items in both languages. However, these instruments have not been validated in Malaysia.

### Data analysis

Statistical analysis was carried out based on an intention-to-treat principle. Descriptive statistics were used to describe demographic data, clinical characteristics variables, and instrument scores. The mixed design ANOVA was performed to determine the effects of self-efficacy enhancing program on foot self-care behaviour, foot care self-efficacy (efficacy expectation), foot care outcome expectation, knowledge of foot care and quality of life between the two groups at baseline, week-4 and week-12. Results of inferential analyses was presented as 95% confidence intervals (95% CI) and p-values. Throughout the analysis, a p-value of less than 0.05 was considered statistically significant. Data was analysed independently using IBM Statistical Package for the Social Sciences software version 22 [56].

### Ethical considerations

Ethical approval was obtained from the Ethics Committee for Research Involving Human Subjects, Universiti Putra Malaysia [JKEUPM Ref No. FPSK (FR15) P021] (10/08/2017) and the Department of Social Welfare/ Jabatan Kebajikan Masyarakat Malaysia (Ref No. JKMM 100/12/5/2: 2015 / 003) (12/11/2017). A formal letter from the Department of Community Health, Faculty of Medical and Health Sciences, UPM was issued to the director of each elderly care facility in Peninsular Malaysia so that all the staff would be aware and permission given for the study to commence (Ref No. UPM/FPSK/JKK/600-1/PhDGS40555). Participants were briefed by the main researcher about the topic of study prior to written consent taking. In order to assist the respondents in understanding the reasons for the study, the information sheet and consent form was prepared in Malay language. Participation must be voluntary and they were free to turn down any involvement with this study. Confidentiality and anonymity of the data were ensured as they were entered into a secured computerised database.

This study was submitted for registration with the Australian New Zealand Clinical Trial Registry (20/11/2016) and was registered on the 16th of February 2016 (ACTRN12616000210471). The authors confirm that all ongoing and related trials for this intervention are registered. Due to logistical reasons, data collection for pre-testing was conducted on 4th January 2016 and the actual data collection was done on 22nd of February 2016. Accordingly, any reports and publications were submitted to the respective authorities.

## Results

**Fig 1:**
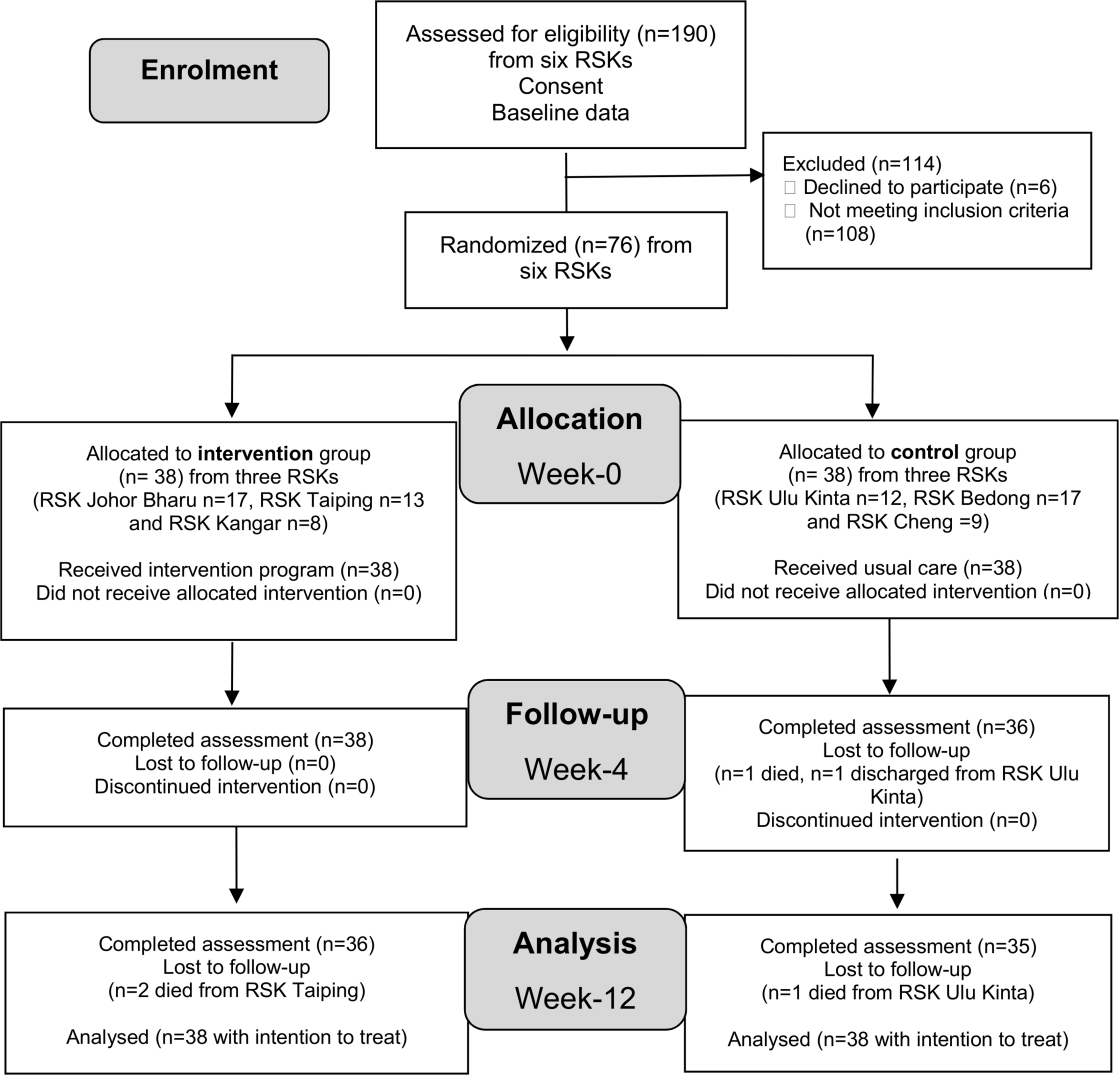
Flow chart of enrolment, allocation, follow up and analysis of the self-efficacy enhancing program on foot self-care behaviour of older adults with diabetes.

A total of 190 older adults with diabetes who stayed in six RKSs were identified from residents’ medical records of the respective clinic. Out of these potential participants, six were not interested in participating. Hence, 184 were screened for eligibility. Of these, 108 older adults with diabetes were excluded. Therefore, only 76 (41.3%) participants were eligible and consented to participate in this study (Fig 1).

For the intervention attendance, all participants (n = 38) in the intervention group were involved in the seminar presentation and week-4 follow-up sessions (100%). All participants (n = 38, 100.0%) in the control group received the usual care, however, only 36 (94.7%) of them were assessed at week-4.

The attrition rate in this study was 2 (2.6%) at week-4 and 3 (6.6%) at week-12. At week-4, one participant from the control group was discharged while another died. A further three participants were lost from follow-up (died) at week-12 from the intervention group (n = 2) and from the control group (n = 1). The final number at week-12 included 36 participants from the intervention group and 35 participants from the control group.

### Participants’ characteristics

Baseline comparisons between the intervention and control groups showed that there were significant differences in ethnicity and treatment (Table 2). At baseline, the average age of the participants in this study was 70 years, where a majority were male, Malay, had not received any formal education or attended primary school, married and have children. The median value for the duration of the participants living in the elderly care facility was three years. On average, the participants have been diagnosed with diabetes for five years. Most of them were on oral medication(s), have co-morbid disease(s), reported that they never received any diabetes education, non-smokers and reported of no history of hospitalisation related to diabetes three months prior to baseline assessment (Table 2).

**Table 2:** Baseline of participants according to demographic data and clinical characteristics by groups (n = 76).

Variable		IG (N = 38)	CG (N = 38)	All (N = 76)	Test Statistics
Age^a^	Mean (± SD)	70.13 (7.73)	69.39 (7.38)	69.76 (7.51)	0.425
Gender^c^					
	Male	24 (63.2)	30 (78.9)	54 (71.1)	2.303
	Female	14 (36.8)	8 (21.1)	22 (28.9)	
Ethnicity^c^					
	Malay	16 (42.1)	25 (65.8)	41 (53.9)	4.290*
	Non-Malay	22 (57.9)	13 (34.2)	35 (46.1)	
Education level^c^					
	Never/ primary	26 (68.4)	27 (71.1)	53 (69.7)	0.062
	Secondary/ tertiary	12 (31.6)	11 (28.9)	23 (30.3)	
Marital status^c^					
	Single	16 (42.1)	14 (36.8)	30 (39.5)	0.220
	Married	22 (57.9)	24 (63.2)	46 (60.5)	
Having child^c^					
	No	17 (44.7)	19 (50.0)	36 (47.4)	0.211
	Yes	21 (55.3)	19 (50.0)	40 (52.6)	
Duration of stay^b^	Median (IQR)	4.00 (6)	3.00 (6)	3.00 (6.0)	630.00
Fasting blood glucose^a^	Mean (± SD)	7.62 (2.60)	8.79 (3.61)	8.21 (3.18)	-1.615
Diabetes duration^b^	Median (IQR)	6.00 (12)	5.00 (12)	5.00 (10.0)	586.50
Treatment^c^					
	Oral only	21 (55.3)	33 (86.8)	54 (71.1)	9.212*
	Insulin (alone or with oral)	17 (44.7)	5 (13.2)	22 (28.9)	
Co-morbidity^c^					
	No	7 (18.4)	14 (36.8)	21 (27.6)	3.224
	Yes	31 (81.6)	24 (63.2)	55 (72.4)	
Diabetes education^c^					
	No	20 (52.6)	28 (73.7)	48 (63.2)	3.619
	Yes	18 (47.4)	10 (26.3)	28 (36.8)	
Smoking^c^					
	No	27 (71.1)	20 (52.6)	47 (61.8)	2.732
	Yes	11 (28.9)	18 (47.4)	29 (38.2)	
Hospitalization^d^					
	No	35 (92.1)	38 (100.0)	73 (96.1)	p = 0.240
	Yes	3 (7.9)	0 (0.0)	3 (3.9)	

IG = Intervention group

CG = Control group

^a^independent t-test was used to compare the means of two groups for a normally distributed data

^b^Wilcoxon-Mann-Whitney test was used to compare the medians of two groups for a skewed data

^c^Chi-square test was used to compare the proportion of two groups for categorical data

^d^Fisher's exact (2-sided) test was used to compare the proportion of two groups for categorical data (if the expected value of each cell was less than five)

*p-value <0.05 = statistically significant

### Foot self-care behaviour, foot care self-efficacy (efficacy expectation), foot care outcome expectation, knowledge of foot care and quality of life

**Fig 2:**
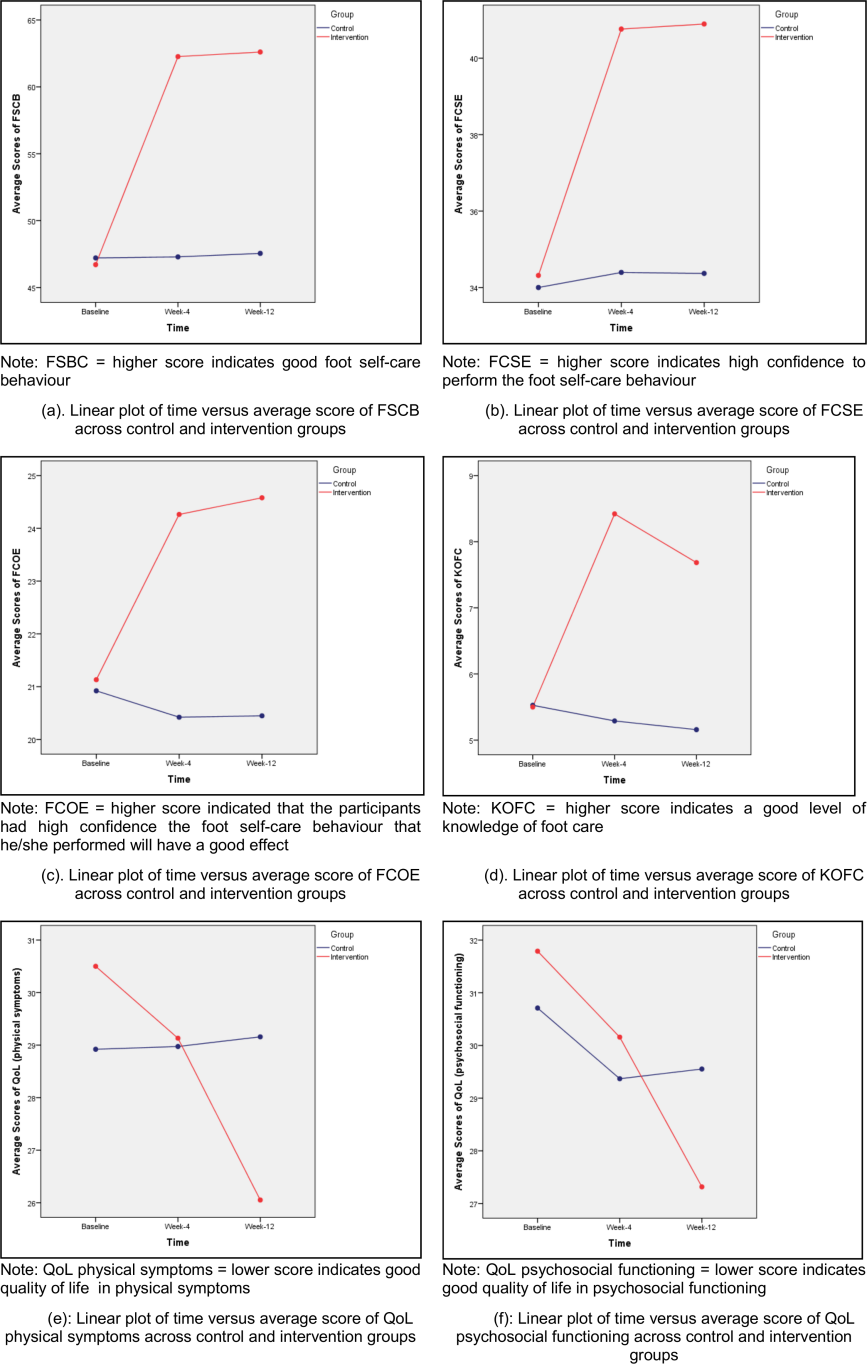
(a)—(f). The linear plot of the time effects for each variable across two groups.

Table 3 shows the descriptive analysis for the variables for each of the three time factors for both groups. The analysis indicated that foot self-care behaviour, foot care self-efficacy (efficacy expectation), foot care outcome expectation, QoL physical symptoms and QoL psychosocial functioning scores improved from the baseline, to week-4, and week-12 for the intervention group. The knowledge of foot care score increased from baseline to week-4, however the score reduced from week-4 to week-12. For the control group, the scores for all variables can be considered as consistent from time to time. Fig 2(A)–2(F) shows the linear plot of the time effects for each variable across two groups.

**Table 3:** Result of baseline, week-4, week-12, and changes (%) between the groups on FSCB, FCSE, FCOE, KOFC, QoL physical symptoms and QoL psychosocial functioning (n = 76).

Variable	Group	Time Based			Change from baseline to week-4 (%)	Change from week-4 to week-12 (%)	Change from baseline to week-12 (%)
		Baseline(M ± SD)	Week-4(M ± SD)	Week-12(M ± SD)			
FSCB							
	IG	46.71 ± 9.80	62.26 ± 8.90	62.61 ± 7.54	14.27	0.28	14.54
	CG	47.21 ± 9.86	47.29 ± 9.20	47.55 ± 7.30	0.08	0.27	0.36
FCSE							
	IG	34.32 ± 5.32	40.76 ± 5.55	40.89 ± 4.91	8.58	0.16	8.74
	CG	34.00 ± 5.31	34.39 ± 5.09	34.37 ± 4.69	0.57	-0.03	0.54
FCOE							
	IG	21.13 ± 2.96	24.26 ± 2.87	24.58 ± 3.08	6.90	0.66	7.55
	CG	20.92 ± 3.98	20.42 ± 3.70	20.45 ± 3.30	-1.21	0.07	-1.14
KOFC							
	IG	5.50 ± 3.41	8.42 ± 1.88	7.68 ± 1.49	20.98	-4.60	16.54
	CG	5.53 ± 3.37	5.29 ± 3.06	5.16 ± 3.09	-2.22	-1.24	-3.46
QoL physical symptoms							
	IG	30.50 ± 10.83	29.13 ± 10.88	26.05 ± 9.72	-2.30	-5.58	-7.87
	CG	28.92 ± 14.63	28.97 ± 12.67	29.16 ± 12.51	0.09	0.33	0.41
QoL psychosocial functioning							
	IG	31.79 ± 16.03	30.16 ± 15.75	27.32 ± 14.14	-2.63	-4.94	-7.56
	CG	30.71 ± 17.60	29.37 ± 16.57	29.55 ± 14.12	-2.23	0.31	-1.92

FSCB = Foot self-care behaviour

FCSE = Foot care self-efficacy

FCOE = Foot care outcome expectation

KOFC = Knowledge of foot care

QoL = Quality of Life

IG = Intervention group

CG = Control group

M = Mean

SD = Standard Deviation

Change from baseline to week-4 (%) = [(mean week-4 + mean baseline) / (mean week-4—mean baseline) x 100%]

Change from week-4 to week-12 (%) = [(mean week-12 + mean week-4) / (mean week-12—mean week4) x 100%]

Change from baseline to week-12 (%) = [(mean week-12 + mean baseline) / (mean week-12—mean baseline) x 100%]

The equality of error variance was tested with Levene’s Test and Hartley’s F-Max Ratio Test. Based on Levene’s Test, the foot care outcome expectation score at week-4 (Levene’s Statistic = 4.436, p <0.05), knowledge of foot care score at week-4 (Levene’s Statistic = 10.064, p <0.05) and at week-12 (Levene’s Statistic = 20.048, p <0.05) as well as QoL psychosocial functioning score at baseline (Levene’s Statistic = 4.675, p <0.05) do not have an equal error variance across the intervention and control groups since Levene’s statistic was significant at 95% confidence level. However, based on Hartley’s F-Max Ratio test, all the interested variables can be considered to have an equal error variance across the intervention and control groups for each time effect (baseline, week-4, and week-12), since the ratio was less than 3.00 for a sample size in the range of 30 to 60 samples [57]. Therefore, it can be concluded that the error variance for each variable in each time factor can be considered equal across the intervention and control groups.

The results (Table 4) indicated that foot self-care behaviour, foot care self-efficacy (efficacy expectation), foot care outcome expectation and knowledge of foot care scores significantly changed across the three time points (p < 0.01). The analysis also showed that there were interaction effects between the variables and group, indicating that the scores of the variables differed in the intervention and control groups across the time points (p < 0.01). In addition, the group effect was also significant (p < 0.01). Therefore, the scores of the variables between the intervention and control groups were significantly different.

**Table 4:** Result of mixed designs ANOVA analyses on FSCB, FCSE, FCOE, KOFC, QoL physical symptoms and QoL psychosocial functioning (n = 76).

Variable	Mauchly’s Test of Sphericity	Test Statisticsª	Effect Sizeᵇ
FSCB	0.605**	62.651**	0.458
Time(FSCB)*Group		59.321**	0.445
Group Effect	N/A	F (1,74) = 30.308**	0.291
FCSE	0.790**	38.157**	0.340
Time(FCSE)*Group		30.187**	0.290
Group Effect	N/A	F (1,74) = 17.304**	0.190
FCOE	0.794**	13.375**	0.153
Time(FCOE)*Group		24.120**	0.246
Group Effect	N/A	F (1,74) = 16.335**	0.181
KOFC	0.774**	12.365**	0.143
Time(KOFC)*Group		18.515**	0.200
Group Effect	N/A	F (1,74) = 11.136**	0.131
QoL (physical symptoms)	0.700**	5.725**	0.072
Time (QoL physical symptoms)*Group		7.117**	0.088
Group Effect	N/A	F (1,74) = 0.030	0.001
QoL (psychosocial functioning)	0.854**	8.572**	0.104
Time (QoL psychososocial fun.)* Group		3.643**	0.047
Group Effect	N/A	F (1,74) = 0.001	0.001

ªSince Mauchly’s Test of Sphericity was significant, the Greenhouse-Geisser test statistics adjustment was used if the value of Mauchly’s estimates of sphericity was less than 0.75, whereas the Huynh-Feldt test statistic was used if the value of Mauchly’s estimates of sphericity was above 0.75. For the Group Effect test statistics, conventional F test was used

ᵇThe Partial Eta Square (η^2^) was used to estimate the effect size of the test

FSCB = Foot self-care behaviour

FCSE = Foot care self-efficacy (efficacy expectation)

FCOE = Foot care outcome expectation

KOFC = Knowledge of foot care

QoL = Quality of Life

N/A = Not Applicable

**p <0.01

*p <0.05.

In terms of QoL for physical symptoms and psychosocial functioning, the results indicated that the score of the variables changed significantly across the three time points (p < 0.01). QoL scores also differed significantly in the intervention and control groups across time points, since the interaction effect between QoL (physical symptoms and psychosocial functioning) and group effect were significant (p < 0.01). However, QoL physical symptoms and QoL psychosocial functioning scores did not show a statistical significant difference across the intervention and control groups (p > 0.05), as the QoL (physical symptoms and psychosocial functioning) were equal for both groups (Table 4).

Table 5 summarises the results of the multiple comparison analysis and pairwise comparisons across time. The analysis indicated that foot self-care behaviour, foot care self-efficacy (efficacy expectation), foot care outcome expectation, knowledge of foot care and QoL psychosocial functioning scores showed statistical significant differences, where the week-4 and week-12 scores were better than the baseline scores from baseline to week-4 and from baseline to week-12 (p < 0.05). For the QoL physical symptoms, the analysis indicated that this pair showed a statistical significant difference from baseline to week-12 and from week-4 to week-12 (p < 0.05).

**Table 5:** Multiple comparison analysis for pairwise comparisons on FSCB, FCSE, FCOE, KOFC, QoL physical symptoms and QoL psychosocial functioning (n = 76).

Variable	Multiple Comparison Analysisª			95% Confidence Intervalª		
	Baseline vs. Week-4	Week-4 vs.Week-12	Baseline vs.Week-12	Baseline vs.Week-4	Week-4 vs.Week-12	Baseline vs.Week-12
FSCB	7.82** (0.99)	0.30(0.51)	8.12** (0.89)	(5.40, 10.23)	(-0.96, 1.56)	(5.94, 10.30)
FCSE	3.42** (0.52)	0.05(0.34)	3.47** (0.49)	(2.15, 4.69)	(-0.77, 0.88)	(2.27, 4.68)
FCOE	1.32** (0.30)	0.17(0.26)	1.49** (0.38)	(0.59, 2.05)	(-0.46, 0.80)	(0.56, 2.41)
KOFC	1.34** (0.29)	-0.43(0.21)	0.91*(0.32)	(0.64, 2.04)	(-0.94, 0.07)	(0.12, 1.70)
QoL physical symptoms	-0.66(0.72)	-1.45*(0.43)	-2.11*(0.72)	(-2.43, 1.11)	(-2.50, -0.40)	(-3.86, -0.35)
QoL psychosocial functioning	-1.49*(0.61)	-1.33(0.62)	-2.82** (0.80)	(-2.97, -0.01)	(-2.85, 0.19)	(-4.78, -0.86)

ªThe Bonferroni Adjustment methods of multiple comparison analysis was used; Value reported in the multiple comparison analysis is a Mean Differences values

Number in the bracket is a Standard Error value

**p <0.01

*p <0.05.

Table 6 shows the multiple comparison analysis of the variables of interest across the intervention and control groups. The analysis indicated that the foot self-care behaviour, foot care self-efficacy (efficacy expectation), foot care outcome expectation, and knowledge of foot care scores for the intervention group were significantly greater than that of the control group (p < 0.01). On the other hand, the QoL (physical symptoms and psychosocial functioning) for the intervention group were not significantly different as compared to the control group scores (p > 0.05).

**Table 6:** Multiple comparison analysis for group effect on FSCB, FCSE, FCOE, KOFC, QoL physical symptoms and QoL psychosocial functioning (n = 76).

Variable	Group	Mean ± SE	Comparison Analysisª	95% Confidence Intervalª
FSCB				
	IG	57.19 ± 1.26	9.84**(1.79)	(6.28, 13.40)
	CG	47.35 ± 1.26		
FCSE				
	IG	38.66 ± 0.75	4.40**(1.06)	(2.29, 6.51)
	CG	34.25 ± 0.75		
FCOE				
	IG	23.33 ± 0.48	2.73**(0.68)	(1.38, 4.07)
	CG	20.60 ± 0.48		
KOFC				
	IG	7.20 ± 0.40	1.88**(0.56)	(0.76, 3.00)
	CG	5.33 ± 0.40		
QoL physical symptoms				
	IG	28.56 ± 1.87	-0.46(2.65)	(-5.73, 4.82)
	CG	29.02 ± 1.87		
QoL psychosocial functioning				
	IG	29.75 ± 2.49	-0.12(3.53)	(-7.15, 6.91)
	CG	29.88 ± 2.49		

ªThe Bonferroni Adjustment methods of comparison analysis was used; value reported in the comparison analysis is a Mean Differences values

Number in the bracket is a Standard Error value

SE = Standard Error

**p <0.01.

## Discussion

The study found that there were improvements in the foot self-care behaviour, foot care self-efficacy (efficacy expectation), foot care outcome expectation and knowledge of foot care following the program. The score for foot self-care behaviour of the intervention group was greater than that of the control group from baseline to week-4 and from baseline to week-12. The findings are in line with several previous intervention studies conducted on foot self-care among the older adults with diabetes, regardless of the different methodology, format and procedures [17, 42, 58]. In contrast, one intervention study found that the score for foot care behaviour were the similar between the two groups after three months following the intervention program [59].

The instrument and intervention module used in this self-efficacy enhancing program was designed for older adults with diabetes. The program activities were in line with the four self-efficacy sources; performance accomplishment, vicarious experience, verbal persuasion and physiological and emotional states [11, 12]. Verbal persuasion was utilised during the seminar presentation, one-to-one discussion and weekly visits. Building trust and rapport with participants were important skills. The older adults often learn from personal experience[1], therefore, performance accomplishment were used when the participants continued practicing the foot self-care behaviour. Sharing experience with each other and referring to the pamphlet were the components of social and symbolic modelling in vicarious experience. Older adults with diabetes could develop their skills together with their peers [60]. Participants who had difficulties in performing the behaviour were assessed for physical and emotional states.

Previous intervention studies demonstrated similar findings, reporting an improvement in the diabetes foot self-efficacy scores before and after implementation of the intervention program [19, 61]. However, the finding from this recent study is inconsistent with other studies [17, 62]. Such intervention studies are recommended to be further evaluated with a more rigorous design. For the foot care outcome expectation, present findings indicated that the scores increased at week-4 and week-12 following the program. This finding is similar with a previous study [63], however, the intervention involved general measures of diabetes self-care education.

The participants in this present study were reported to be more confident in undertaking the foot self-care behaviour after the program. Besides, the participants believed that they can protect their feet if they perform foot care properly. Self-efficacy (efficacy expectation) and outcome expectation are two components in Bandura’s theory. According to Bandura, these principles involved the process of judgment by a person to perform specific tasks that he would like to achieve to certain outcomes [64]. A person will be more likely to succeed in their skills if they have a high belief that they can perform the specific behaviour [63].

The level of knowledge in the intervention group significantly increased between baseline and week-4 follow-up. Similar to previous studies, it was demonstrated that the knowledge scores increased after three months intervention commenced [61, 62]. Meanwhile in other RCT studies, there was no significant difference in the foot care knowledge scores among the older adults with diabetes between the groups [17, 65].

The knowledge level of this present study was slightly reduced between week-4 and week-12 of the study duration. Likewise, a previous study revealed that foot care knowledge level increased immediately after the education program, however, the knowledge score reduced after three months of intervention [66]. It can be concluded that older adults with diabetes requires certain level of cognitive skills in memorising specific tasks. Besides, advanced age, duration of diabetes associated with co-morbidity, smoking, low literacy, high glycaemic level control and physical inactivity might have a great impact in influencing cognitive function [67]. The program had been effective in improving the knowledge level among older adults with diabetes, but, it would be effective if the information can be delivered regularly [60].

In this study, the overall QoL physical symptoms and QoL psychosocial functioning improved from baseline to week-12. However, the scores of both variables were the same between the two groups. The finding in this present study appears similar to previous RCT studies carried out for older adults with diabetes [58, 68]. This finding is difficult to explain as the concept is broad and often described as the objective and subjective dimension of the quality of life [69]. Concerns on aspects of health status, socio-economic, culture, environment and spiritual might influence the quality of life [70]. A previous study had reported that the quality of life among older adults with diabetes was highly influenced by socio-demographic background rather than clinical or health status [71].

For example, financial issues, social and living arrangements may have interfered with the QoL of most of the older adults living in elderly care facility. The elderly care facility generally provides them with food and treatment, clothing, toiletries, bedding and linens. The older adults received a monthly allowance from the government. Some of the residents make handicraft or plant vegetables in a garden for extra income. Besides, some of them are unwilling to stay in the RSKs, but they have no choice due to age factor and no relative to take care of them. Functional limitations in performing ADLs independently, disturbances in social relationship with relatives and disruption in emotional states with other residents and staff in the RSKs would affect the quality of life.

### Limitations of the study

There are several limitations of this study. First and foremost, a duration of 12 weeks for the program was inadequate to assess the sustainability of the program on the outcome measures. Second, the enrolment rate in this study was low. The potential respondents underwent several screening processes and quite a number did not meet the inclusion criteria. Besides, this study was conducted among the elderly with diabetes living in only six RSKs with a relatively small sample. In order to provide a more valid analysis, therefore, a larger number of clusters are needed in future research.

In this study, the interview was conducted during data collection process. Reporting bias may occur when the participants were unable to answer the questionnaire independently. Some of the participants in the control group might have obtained information from other sources (information bias). With repeated answering of the same questionnaires, the participants might be able to recall the questions that they have previously answered. Another limitation is that the close-ended questionnaire design inhibits the researcher the opportunity to explore the details of the participants’ feelings. Finally, other clinical paramaters such as laboratory investigations were not measured in this study, as it would provide additional findings.

## Conclusion

In conclusion, self-care behaviour, foot care self-efficacy (efficacy expectation), foot care outcome expectation and knowledge of foot care improved in the intervention group compared to the control group after 12 weeks of program implementation. However, there were no significant improvements for QoL physical symptoms and QoL psychosocial functioning between the intervention and control groups. In the future, a self-efficacy enhancing program should use a larger sample and it can be conducted in other elderly care facility in Malaysia. Evaluation of long term effects (more than 12 weeks follow-up) is warranted to determine the sustainability of the program in improving foot self-care behaviour.

To the best of the researcher’s knowledge, there was no study done on the effects of foot self-care behaviour among the older adults with diabetes in Malaysia. This would be the first RCT evaluating the effects of foot self-care behaviour using a self-efficacy concept among the older adults with diabetes living in elderly care facility in Peninsular Malaysia.

This study found that, at baseline, majority older adults with diabetes reported did not receive health education on diabetes. Hence, older adults with diabetes in elderly care facility should be encouraged to attend diabetes education programs and advised to practice proper foot self-care behaviour. The healthcare provider need to help the older adults to understand the importance of the preventive strategies to reduce complications which includes regularly foot inspections and examinations. It is to be hoped that the findings from this study can be disseminated to policy makers or public health professionals concerned.

## Supporting information

S1 File Clinical trial protocol. (PDF)

S2 File CONSORT extension for non-pharmacologic treatment checklist. (DOC)

S3 File Proposal for ethical approval. (PDF)

S4 File Research ethics committee approval letter (Universiti Putra Malaysia). (PDF)

S5 File Research ethics committee approval letter (Social Welfare Department Malaysia). (PDF)

S6 File Subject information sheet. (PDF)
