# Supplementary material for: The effects of self-efficacy enhancing program on foot self-care behaviour of older adults with diabetes: A randomised controlled trial in elderly care facility, Peninsular Malaysia
Source: PLoS One. 2018 Mar 13;13(3):e0192417. doi: 10.1371/journal.pone.0192417 (PMC5849313; doi:10.1371/journal.pone.0192417)
Supplement: S1 File — (PDF) [file pone.0192417.s001.pdf]

“Effects of Self-Efficacy Enhancing Program on Foot Self-Care Behaviour of Elderly with Diabetes in Rumah Seri Kenangan, Peninsular Malaysia”

Submitted to the Department of Community Health,
Faculty of Medicine and Health Sciences, Universiti Putra Malaysia, as a partial fulfilment of the requirement for course
Doctor of Philosophy

Supervisor: Associate Professor Dr Hejar Abdul Rahman
Co-supervisor 1: Dr Halimatus Sakdiah Minhat
Co-supervisor 2: Associate Professor Dr Sazlina Shariff Ghazali

Student’s name: Siti Khuzaimah Ahmad Sharoni (GS40555)

Abstract

Background: Diabetes related foot problems can affect health status. Foot self-care behaviour is an essential management in preventing foot complications of diabetes. Self-efficacy theory has been reported to improve foot self-care behaviour of elderly with diabetes.

Objective: Main aim here is to develop, implement and evaluate the effects of self-efficacy enhancing program on foot self-care behaviour of elderly with diabetes in Rumah Seri Kenangan, Peninsular Malaysia.

Methods: Randomised Control Trial Design, single blind with two groups; intervention and control will be used as a study design. The study will be conducted in Rumah Seri Kenangan, Peninsular Malaysia. A probability cluster sampling will been chosen and a total of 142 respondents who met the inclusion criteria will be invited for participation. During education program, the intervention group will be given a seminar and demonstration about foot self-care behaviour. Meanwhile the control group will receive a usual health care. A series of follow up will be continued for 12 weeks. Pre and post questionnaires which includes demographic data and clinical characteristics, foot self-care behaviour, foot care efficacy expectation, foot care outcome expectation, knowledge on foot care and quality of life will be distributed within a given time frame. The data will address each of the research questions by descriptive and inferential statistics.

Conclusion: The aim of the education program will that, after completed the program, the foot self-care behaviour, foot care efficacy expectation, foot care outcome expectation, knowledge on foot care and quality of life will be improved with respect to the program delivered. Future expectation, the Self-efficacy theory can be incorporated in diabetes education to enhance foot self-care behaviour and improve physical and psychosocial outcome of elderly with diabetes.

Chapter 1

INTRODUCTION

1.1 Introduction

Self-efficacy, psychosocial aspects and quality of life are essential factors in promoting foot self-care behaviour of elderly with diabetes. This chapter will explain about the background information of elderly with diabetes and its related problem statements, the significance and objectives of the study as well as its definition of terms.

1.2 Background

Diabetes is the most common of non-communicable disease affecting the elderly. Diabetes Mellitus is the hyperglycaemic state that results from defects in insulin secretion, insulin action or both constitute the group of metabolic disease (Clinical Practice Guideline (CPG), 2009; Burke et al., 2011). There are three main types of diabetes; type 1 diabetes known as insulin-dependent diabetes; type 2 diabetes known as non-insulin-dependent diabetes and gestational diabetes (Burke et al., 2011).

Type 2 diabetes (commonly known as diabetes) accounts for 90% - 95% of all type of diabetes cases (ADA, 2014). It has been a consistent finding from previous studies that a large proportion of all patients found to have diabetes had not been previously diagnosed (International Diabetes Federation (IDF), 2013). There are non-modifiable risk factors of diabetes such as age, gender, ethnicity and genetics (IDF, 2013). Sedentary lifestyle, obesity, changes in dietary habits and reduction in physical activities are the modifiable factors that contribute to higher prevalence of diabetes (Burke et al., 2011).

Most individuals with diabetes are asymptomatic or may be related by symptoms of polydipsia, polyuria, blurring of vision, fatigue, paresthesias and skin infections (ADA, 2014). However, the elderly often describe symptoms such as fatigue, blurred vision, change in weight (gain or loss) and infections such as foot/leg wound, virginities, or urinary tract infection, numbness of extremities and vision changes (Feinglos & Bethel, 2008).

The CPG (2009) stated the diagnostic criteria for diabetes must be confirmed by measurement of venous plasma glucose. A value for diagnosis of diabetes when is a venous plasma glucose level (fasting) is ≥ 7.0 mmol/L and or venous plasma glucose level (random) ≥ 11.1 mmol/L (CPG, 2009). In the symptomatic individual, one abnormal glucose value is considered diagnostic (CPG, 2009). However, for the asymptomatic individual, 2 abnormal glucose values are required in diagnose of diabetes (CPG, 2009).

There are several treatments in managing diabetes. Most elderly with diabetes are usually taking an oral hypoglycaemic medication, like metformin but in case of severe and uncontrolled diabetes, they may require insulin therapy (Vischer et al., 2009). Metformin was the most frequently prescribed agent (82.9%), followed by Sulphonylureas (73.5%) but only 2.9% of patients were treated with insulin only or combination with oral hypoglycaemic agents (Ooi et al., 2011). Letchuman et al., (2010) stated among people with known diabetes, only 84.3% were found to be on modern medications; 77.1% were on oral, 3.1% on insulin alone and 4.1% were treated with both oral and insulin.

Complications of diabetes commonly are divided into two categories; acute and chronic diseases. Acute complications of diabetes diabetic such as diabetic ketoacidosis (DKA), hyperglycemia hyperosmolar state (HHS) and hypoglycemia are serious and life-threatening. The large number of associated with chronic complications mostly classified as macrovascular and microvascular diseases (Meiner & Lueckenotte, 2006). The major killer in patient suffering from the illness are macrovascular disease such as myocardial infarction, cerebrovascular and peripheral vascular disease and co-morbidity ((American College of Foot and Ankle Surgeons, 2006; Rizvi, 2007; Ooi et al., 2011; IDF, 2013). Meanwhile the most common microvascular complications are diabetic retinopathy, nephropathy and neuropathy (American College of Foot and Ankle Surgeons, 2006; Fowler, 2008; Burke et al., 2011; Ooi et al., 2011; Bhuvaneswar, Epstein, & Stern, 2007; IDF, 2013).

Diabetic neuropathy is often leads to foot problems and leg amputation. Diabetic foot problem is accountable for morbidity and permanent disability (Singh et al., 2013).The most common of diabetic foot abnormalities are calluses, fissures, deformities, and loss of the protective sensation of pain (Chin & Huang, 2013). These diabetic foot problems may lead to the development of ulcers. The cases of foot ulcers and amputations may be due to poor hygiene, and bare-foot walking associated to foot injury (Boulton, 2005). Foot ulcers commonly become infected, may develop gangrene and result in major lower limb amputation (below knee or above knee amputation) and leads to loss of life (Nather, 2007; CPG, 2009; Bhuvaneswar, Epstein, & Stern, 2007; IDF, 2013).

Pain, disfigurement, impaired physical functioning, permanent changes in lifestyle, low self-esteem and disruption quality of life were associated with psychological problems among diabetes patients (Leung, 2007; Cai-Xia et al., 2008; Shu-Fang et al., 2011; Saleh et al., 2014; Kargar et al., 2014). The common feeling amongst diabetes patients is that losing a limb makes one a ‘cripple’, and they would rather lose their lives than a limb (Ang & Lim, 2013). Elderly with diabetes reported having unhealthy life both physically and mentally as compared to without the disease (OR: 1.64; 95% CI: 1.20, 2.23) and this contribute to diminished quality of life (Brown et al., 2004). Episodes of admission in hospital are associated with emotional and financial strains on the patient (Ang & Lim, 2013).

Diabetes is a major public health problem as it causes of comorbidities, cognitive and functional disabilities, mortality as well as demands on health care facilities and social care resources (Brown et al., 2004; American College of Foot and Ankle Surgeons, 2006; Nugent, 2008; Ooi et al., 2011; Awc, Zaim, Helmy, & Ramdhan, 2014). The charges for treating patients with diabetic foot ulcer and amputation have a

significant impact on the cost and impose a large economic burden on the public health care system (Cook & Simonson, 2012; Ang & Lim, 2013). Elderly with diabetes have considerable functional impairment associated have reduced health status (Sinclair et al., 2008). Patients with complication of diabetes are hospitalized 1.5 – 3 times more than without the disease (Leonard et al., 2004). Length of stay in the hospital was 47% longer after a major amputation than a toe amputation and the average length of stay was 10 days with an in-hospital mortality proportion of 1.29% (Cook & Simonson, 2012).

1.3 Problem statement

1.3.1 Prevalence of diabetes (worldwide and Malaysia)

The incidence of diabetes is now exponentially increased and the disease is one of the most common chronic diseases globally. In 2013, there is approximately 382 million people have diabetes in the world (IDF, 2013). Diabetes is the fifth leading cause of death worldwide accounting for 4.6 million annually (Roglic & Unwin, 2009).

The number of people with diabetes in the world is expected to increase between 2000 and 2030 due to population growth, obesity, aging and urbanization (Shaw, Sicree, & Zimmet, 2010; Wild et al., 2004; Noh et al., 2007). The number of elderly constitutes more than 11.1% of the world's population and by 2035, is expected to rise to 1.5 billion – 17.6% of the population (IDF, 2013). The prevalence of diabetes among elderly is predicted to increase to 366 million in 2030 from 171 million in 2000 (Caughey et al., 2010). The IDF (2013) stated the global prevalence of diabetes in elderly to be 18.6%, more than 134.6 million people, accounting for over 35% of all cases of diabetes in adults and by 2035, that number is projected to increase beyond 252.8 million.

In many developed countries prevalence of diabetes has reached an epidemic proportion (IDF, 2013). The greatest rise in the prevalence of diabetes in the next twenty years in developing countries (Leung, 2007). According to The Malaysian National Health Morbidity Survey III 2006, by 2025, the greatest number of person with diabetes is expected to change to the South East Asia Region, with estimated prevalence of 13.5% and in number with some 145 million people (Letchuman et al., 2010). It is estimated there will be more than 82 million of the elderly with diabetes in developing countries in year 2030 (Wild et al., 2004).

Malaysia is at an epidemiological transition where non-communicable diseases (NCDs) are now dominating its burden at a par with those of developed countries (Ambigga et al., 2011). There were 1.9 million (10.11%) of diabetes cases in Malaysia; with (per year) total incident cases of people with diabetes is 1919.24, number of deaths due to diabetes is 24,049 and number of undiagnosed cases of diabetes is 1035.06 (IDF, 2013). The prevalence of diabetes in Malaysian is 22.6%, almost a double increase from 11.6% in 2006 (Wan Nazaimoon et al., 2013). The National Health Morbidity Survey (2011) reported 15.2% (2.6 million) of patients have diabetes, 7.2% are known to have diabetes and 8.0% are previously undiagnosed with diabetes (Ministry of Health Malaysia, 2011). The prevalence of diabetes mellitus reported within one year was 4.0% (Amal et al., 2011). The prevalence for Impaired Fasting Glucose

(IFG) was 4.2% and was significantly higher among the urbanites 4.5% compared to rural folks (3.8%) (Letchuman et al., 2010).

The National Health and Morbidity Survey (NHMS) (2011) has shown that the prevalence of diabetes in Malaysia has increased by 31.0% in 5 year-period, from 11.6% in 2006 to 15.2% in 2011 and the prevalence of diabetes in Malaysia is will be projected to 21.6% by the year 2020 (National Diabetes Registry, 2013). Subsequently, the World Health Organization (WHO) has predicted that in 2030, diabetics' patients in Malaysia will be reached to 2.48 million compared to 0.94 million in 2000 (Mafauzy, 2006). It is not surprising therefore that the prevalence of diabetes also increased from 11.6% in 2006, to 15.2% in 2011, which equates to approximately 2.6 million adults (Mustapha et al., 2014).

The rapid increase of the ageing population means a more estimated accelerated increase in the number of elderly with diabetes of alarming proportion (Ooi et al., 2011). The overall prevalence of diabetes among elderly in Malaysia was 34.4% (Kiau et al., 2014). There was a general increasing trend in diabetes prevalence with age; from 2.0% in the 18-19 years old age group to a prevalence ranging between 20.8 to 26.2% among the 60-64 years old (Letchuman et al., 2010). The average age of type 2 diabetes patients was 60 years old, 41.6% were men and 58.4% were women (National Diabetes Registry, 2013). The prevalence was higher in the urban at 12.2% compared to the rural areas at 10.6% (Letchuman et al., 2010). The risk for diabetes increases with age and approximately half of all cases occur among the elderly (Malaysian Diabetes Association, 2013). By living longer, the number of elderly with diabetes also increases.

Figure 1.1 illustrates the age groups in the population with the highest proportion of diabetes. The grey line is the distribution of diabetes prevalence by age for the world; the black line is the distribution for the region; and Malaysia distribution is plotted in the dark black line. Malaysia have more people with diabetes compared to the world average. Also, the prevalence of diabetes in Malaysia is occurring more among the elderly (28.7%) (National Diabetes Registry, 2013).

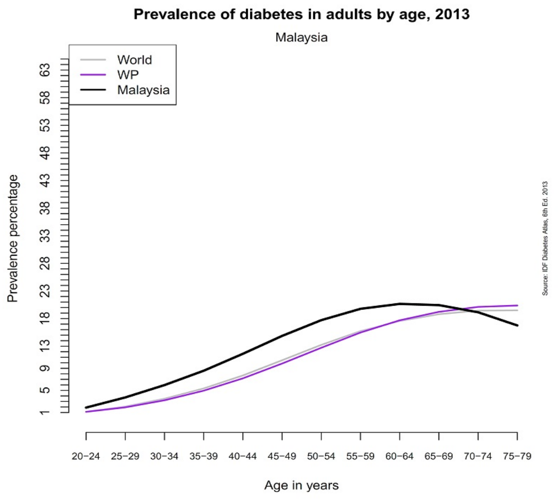

Figure 1.1 Prevalence of diabetes in Malaysia by age, (2013)
source IDF, (2013)

1.3.2 Prevalence of diabetes foot problems (worldwide and Malaysia)

Complication of diabetes can give great impact for the patients. The rates of diabetes complication were high with approximately 27.2% had macrovascular complications and 53.5% had microvascular complications in many countries (Litwak et al., 2013). Neuropathy affects 20%- 50% of people with diabetes (Boulton, 2005). The incidence of developing a foot ulcer among people with diabetes is 15–25% (Cook & Simonson, 2012). Approximately 25% of people with diabetes will have foot problem and is the leading cause of great morbidity and lower limb amputations (Singh, Armstrong, & Lipsky, 2005). The incidence of foot ulceration amongst people with diabetes in developed country is about 2% each year with 1% of them suffer a lower-limb amputation (Boulton, 2005). Subsequent amputations are also problematic, and as many as 68% of amputees will require further amputation within 5 years (Cook & Simonson, 2012). Patients with diabetic foot ulcer are having poor quality of life and has negative effects on the abilities to engage in leisure activities as well as in interactions with family and friends (Ribu et al., 2006).

The prevalence of diabetic foot problems has become a global burden. Despondently, diabetic foot ulcers have been less renowned among health care personnel and researcher (Jeffcoate & Harding, 2003). Diabetic foot is a chronic complication of diabetes which is not accorded the “glamour” status like coronary heart disease, cerebrovascular disease, nephropathy or retinopathy (Singh, Pai, & Yuhhui, 2013).

In Malaysia, elderly with diabetes had suboptimal glycaemic control, the micro and macrovascular complications were prevalent (Ooi et al., 2011). Microvascular, macrovascular and severe late complications were reported about 75%, 28.9% and 25.4% respectively (Mafauzy et al., 2011). Salmiah (2009) stated uncontrolled diabetes problem in this country due to rise in complication and the cost spend for treatment of these patients. The disease burden could overwhelm existing health care systems caused by hospitalization, medications and various disabilities (Hasimah et al., 2010). Mafauzy (2006) stated that diabetes not only lessen the productivity of individual, which later affects nation generation. One third of diabetes patients in Malaysia have poor quality of life (Mafauzy, Hussein, & Chan, 2011). Patients with diabetic foot problems who had significantly lower quality of life often experienced anxiety about when or whether their foot problems will be healed (Mazlina, Shamsul, & Jeffery, 2011).

National Orthopaedic Registry Malaysia (NORM) (2009), stated in Malaysia, 55.3% people who has family history of diabetes will develop diabetic foots problems and people with diabetic foot problems accounted to 35.2% and 21.9% to get chance of neuropathy and skin disorder respectively (Abdullah & Abdullah, 2010). It is estimated the highest prevalence of diabetes complications in Malaysia is neuropathy which accounted with 70% (National Diabetes Registry, 2013). The rates of diabetic complications for neuropathy symptoms and leg amputation were 45.9% and 3.8% respectively (Mafauzy et al., 2011). Patients with diabetic foot problems indicated severe limitations with mobility (Mazlina et al., 2011).

In Malaysia, the incidence and average annual incidence of diabetic foot ulceration is about 10% and 1% respectively in this 10-year-period (Faridah & Azmi, 2009). In 2012, 11.1% of patients suffering for diabetic foot ulcer and 11.0% for amputation (National Diabetes Registry, 2013). NORM (2009) accounted the highest prevalence of diabetes foots and hands reported among the elderly population (38.3%), female (51.8%), Malay (74.6%), urban (53.5%), primary/low education level (49.3%), housewife (35.8%), retired (13.8) and unemployed (11%) (Abdullah & Abdullah, 2010). The NORM (2009) also reported 29.7% cases related to diabetes foot problems has a previous operations performed, 61.8% readmission related too diabetic foot problem, 14 days duration stayed in ward and 37.6% of them were noncompliance to the disease treatment (Abdullah & Abdullah, 2010). The highest risk factors of foot problems in Malaysia were the diabetics are involved with lots of walking/ standing while doing activities at work (47.4%), wearing slippers (47.3%), nearly half of this population were barefoot at home (49.5%) (NORM, 2009) (Abdullah & Abdullah, 2010).

Table 1.1 showed the awareness about risk factors of people with diabetes to their foot-care. This report was collected for one year period (from January to December 2009) by the National Orthopedic Registry of Malaysia (NORM) and the Clinical Research Centre (CRC), Ministry of Health Malaysia. The survey involves people with diabetes who were attending in government hospital. The result showed people with diabetes has significant low in diabetes foot awareness; there were only 17.6% of them attends to diabetic foot clinic, 22.8% keep on diabetes booklet, 23.2% apply emollients on their feet, 26.5% wear appropriate shoes and only 27.3% of them have received formal education on foot-care.

Table 1.1 Risk factors (awareness) of diabetes foots and hands source National Orthopaedic Registry Malaysia (NORM) (2009) (Abdullah & Abdullah, 2010)

Awareness of risk factors	n	%
Aware of increased foot/hand problem	779	62.1
Aware: Inspect feet/hand	627	50.0
Aware: Wash feet	830	69.3
Aware: Apply emollients	278	23.2
Aware: Use appropriate shoes	318	26.5
Aware: Attend diabetes foot clinic	211	17.6
Aware: Education on diabetes foot-care (formal)	327	27.3
Aware: Education on diabetes foot-care (informal)	595	49.7
Aware: Keeping on diabetes care booklet	273	22.8

Table 1.2 describes on feet/ hand behaviour (daily, weekly or occasionally) of people with diabetes for one year, from NORM, (2009) (Abdullah & Abdullah, 2010). There were 44.9% of them inspect feet, 64.5% wash feet, 19.1% apply emollient, 23.9% use appropriate shoes and 15.9% attend diabetes foot clinic.

Table 1.2 Feet/ hand behaviour of people with diabetes within one-year-period source National Orthopaedic Registry Malaysia (NORM) (2009) (Abdullah & Abdullah, 2010)

Feet/ hand behaviour	n	%
Inspect feet/hand	563	44.9
Frequent inspect feet		
Daily	206	37.3
Weekly	63	11.4
Occasionally	284	51.4
Wash feet	733	64.5
Frequent wash feet		
Daily	501	69.8
Weekly	36	5
Occasionally	181	25.2
Apply emollients	299	19.1
Frequent apply emollients		
Daily	132	45.4
Weekly	20	6.9
Occasionally	139	47.8
Use appropriate shoes	286	23.9
Frequent use appropriate shoes		
Daily	134	48.9
Weekly	17	6.2
Occasionally	123	44.9
Attend diabetes foot clinic	190	15.9
Frequent attend diabetes foot clinic		
Daily	58	28.3
Weekly	122	59.5
Occasionally	0	0

As seen in Table 1.2, people with diabetes had poor behaviour in managing foot care. Therefore, it required among health care provider to develop effective strategies to monitor diabetes complication for improvement in quality of care (Mafauzy et al., 2011). The government need to reassess the various programs and strategies that have been developed and implemented for the last decade (Letchuman et al., 2010). People with diabetes need awareness and confidence in performing foot self-care behaviour.

1.3.3 Elderly population in Malaysia

There are increasing trends among the Malaysian population in this few decades. The population of Malaysia has doubled in the 18-year-period between 1975 - 2005 from 12.3 - 26.7 million and an increase of 8.3% to 28.96 million is expected between 2005 - 2010 (Amal et al., 2011). Between 1990 and 2020, the population of Malaysia is expected to increase from 18.4 million to 33.3 million - an increase of 80% (Mafauzy, 2000).

Malaysia's population is 28.3 million; the current population of Malaysians aged 65 years and above is around 1.3 million (4.7%) and the proportion of population aged 65 years and over increased to 5.1% as compared with 3.9% in 2000 (Department of Statistics Malaysia, 2010). The number of the elderly has increased over the last two decades to almost 1.4 million in 2000 and is expected to increase further to 3.4 million in 2020 (Department of Statistics Malaysia, 2010). Figure 1.2 showed the number of population by sex and age group, Malaysia, 2000 and 2010.

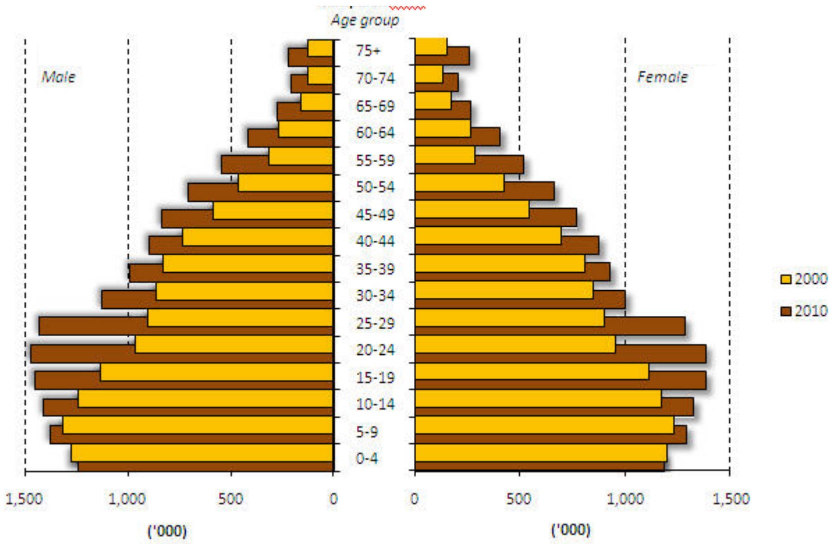

Figure 1.2 Number of population by sex and age group, Malaysia, 2000 and 2010 source (Department of Statistics Malaysia, 2010)

Ageing population becomes a main concern in this country. The life expectancy of male and female is at the age of 71.9 and 76.4 years respectively (Department of Statistics Malaysia, 2010). The number of our population increased due to urbanisation, improvement in nutrition and public health, also advances in medical and health care has contributed to an increased life expectancy and to the demographic changes (Poi, Forsyth & Chan, 2004).

The elderly needs adequate care and facilities in order to get a better quality of life and healthy ageing (Abbas & Saruwono, 2012). However, with recent country development, family members have become increasingly difficult to taking care their elders. Most of them are not available in house during day time because they have to work or having their own commitment. Lack of family members in the home can be a

major barrier to family support (Wan-Ibrahim & Zainab, 2014). As a result, the elders are being abandoned and sent to institutional care or elderly care centre to get a better life.

In Malaysia, there are eleven (two in East Malaysia) public institutionalized centre called Rumah Seri Kenangan (RSK) managed by Social Welfare Department (Sim & Hamid, 2010). The RSK is developed with the aim to give support, care, treatment and protection of elderly to get better quality of life. The entry requirement for them to stay in this instituition are; aged 60 years and above without infectious diseases, do not have close relatives, no permanent homes, able to take care himself, voluntarily to stay in the instituition and able to follow the regulations of entry requirements (Jabatan Kebajikan Masyarakat, 2013).

The services and facilities provided in the RSK included care and protection, medical treatment, guidance and counselling, occupational work and therapy, physiotherapy, recreational and spiritual care (Jabatan Kebajikan Masyarakat, 2013). There are staff from various background who take care of them such as social worker, medical assistance, community nurse, physiotherapist, occupational therapist, religious teacher and support staffs. People in community, non-govenment sector and voluteers, activist, students and friends are needed to give support (money, time and help) for this population. Public are encouraged to visit and participate with their activities such as open day and festival celebration so as this vulnerable group will not feel abandoned and lonely.

However, appropriate treatment and diabetes management is challenging in the institutionalized care (Andreassen et al., 2014). There were approximately 9% of elderly stayed in RSK had diabetes, 6% asthma, 8% ischaemic heart disease, 2% cerebrovascular disease, 4% osteoarthritis and 32% of them were on three or more medications (Visvanathan, Zaiton, Sherina, & Muhamad, 2005). Most of the elderly at RSK Kelantan had diabetes (Sinor, 2013). Study indicated an elderly at care centre are only have a moderate level of well-being (Wan-Ibrahim, Majid, & Zainab, 2014).

Elderly population in institutionalized care need to receive a proper health education to improve diabetes management and quality of life (Garcia & Brown, 2014). Therefore, for additional research addressing the relationship between types of diabetes management and the resident outcomes is needed. The elderly stayed need to voice their experiences regarding diabetes management in the institutionalized care.

Older Malaysian population suffers from multiple and complex health needs which require holistic and comprehensive long term care in the community (Ambigga et al., 2011). Since there are variations in health status, the stakeholder needs to take this issue into account when designing programs for the elderly. However, special programs for the elderly are still deficient. Malaysia need to improve its healthcare system and more needs to be done in preparation of the provision for elderly care (Hairi, Bulgiba, Mudla, & Said, 2011). The primary health care system in Malaysia strategies would necessarily involve inputs and participation at the community level (Mustapha et al., 2014). Active in health promotion interventions in elderly population can reduce vulnerability, enhancing quality of life, and reducing the use of health services (Markle-Reid et al., 2006).

1.4 Significance of study

This study finding may give awareness among elderly with diabetes in RSK to understand the significance of self-efficacy and foot self-care behaviour in the disease management. As a result, it can improve patients' compliance and confidence level in performing foot self-care behaviour. By reducing any potential foot complications, ultimately, it will improve quality of life among elderly with diabetes.

This study can help to improve knowledge on foot care, self-efficacy, quality of life and foot self-care behaviour among elderly with diabetes. In the future, this study may give place for a further, larger study which can reduce the burden of patients to care for their disease and it can reduce the health care cost and staff burden as well as decrease mortality rate and decrease morbidity rate. Thus, the study findings also can offer an important contribution to the government and other healthcare institutions. This information will assist diabetes educator to develop effective strategies for improving the current foot self-care behaviour diabetes education.

Significant variation in cultural belief and expectation has a major impact on how diabetes was managed by patients (Munshi & Lipsitz, 2007; Weinger, 2007). There were least publications about knowledge on foot care, self-efficacy, quality of life and foot self-care behaviour among elderly with diabetes in Malaysia especially in institutionalized care. Therefore, this research would like to contribute new findings and bridging the gaps related to this issue. In the future, this study finding may give place for a further and larger study which can reduce the disease burden and support diabetes educator to implement a therapeutic caring process for elderly with diabetes.

1.5 Objectives

1.5.1 General objective

The general objective of this study is to develop, implement and evaluate the effects of self-efficacy enhancing program on foot self-care behaviour of elderly with diabetes in Rumah Seri Kenangan, Peninsular Malaysia.

1.5.2 Specific objectives

1) To determine the:
   - demographic data of respondents
   - clinical characteristics of respondents
   - foot self-care behaviour of respondents
   - foot care efficacy expectation of respondents
   - foot care outcome expectation of respondents
   - knowledge on diabetes foot care of respondents
   - quality of life of respondents

2) To develop and to implement the self-efficacy enhancing program on foot self-care behaviour of elderly with diabetes

3) To determine the effects of self-efficacy enhancing program on quality of life, knowledge on diabetes foot care, foot care efficacy expectation, foot care outcome expectation and foot self-care behaviour of elderly with diabetes between group (intervention and control) at Time 1 (before the intervention education program), Time 2 (four weeks after the intervention education program) and Time 3 (12 weeks after the intervention education program)

4) To determine the effects of self-efficacy enhancing program on quality of life, knowledge on diabetes foot care, foot care efficacy expectation, foot care outcome expectation and foot self-care behaviour of elderly with diabetes within group (intervention and control) at Time 1 (before the intervention education program), Time 2 (four weeks after the intervention education program) and Time 3 (12 weeks after the intervention education program)

1.5.3 Research questions

Does this self-efficacy enhancing program improves the foot self-care behaviour, foot care efficacy expectation, foot care outcome expectation, knowledge on diabetes foot care and quality of life of elderly with diabetes?

1.6 Theoretical definition

The definition in this study as follows:

1.6.1 Foot self-care behaviour

In this study, foot self-care behaviour will be rate on the number of days (for one week) patients perform a certain behaviour such as examine the bottoms of feet and between the toes, wash and dry between toes, apply moisturizing lotion to feet, nail care, check the inside of the shoes and break in new shoes slowly (Chin & Huang, 2013).

1.6.2 Foot care self-efficacy (foot care efficacy expectation)

In this study, foot care self-efficacy describes how confident are elderly with diabetes in undertaking on foot self-care behaviour (Sloan, 1998).

1.6.3 Foot care outcome expectation

Foot care outcome expectation in this study can be defined on self-belief (level of confidence) that a foot behaviour will have the desired effect.

1.6.4 Knowledge on foot care

In this study, knowledge on foot care is information regarding diabetes foot complications, the risk factors and the foot care that should be practiced for elderly with diabetes.

1.6.5 Quality of life

In this study, quality of life of elderly with diabetes will be measured regarding the foot problems (if any) that may effect on activity daily living and well-being (Vileikyte, Peyrot, & Bundy, 2003).

1.6.6 Elderly

Elderly can be defined as a person who aged 60 years and above (Department of Statistics Malaysia, 2010).

1.6.7 Diabetes

In this study, the researcher will select elderly with diabetes if they are diagnosed as having diabetes by medical doctor according to the clinical criteria based on positive venous plasma glucose level (fasting ≥ 7.0 mmol/L and/ or random≥ 11.1 mmol/L) (CPG, 2009) to participate in this study.

1.7 Limitation of study

Research limitations are primarily related to the research design. However, most of the limitations will be minimized through strategies incorporated as part of the research process. Bias may take place when the respondents answer the questionnaire not in a conducive manner. For example, respondents may not be able to answer the questionnaire independently because of having hearing problems, blurring of vision and did not use their spectacles. Some of the respondents may be illiterate so as difficult to follow instruction during the intervention and data collection process. However, the researcher will interview the respondents who are not able to read or write.

The possibility of report bias may take place when some of the respondents who are involved with this study may report higher score in the questionnaire given because they may receive information from other sources (books, mass media and health provider in the institutionalized care center). In addition, some patients may refuse to participate because of personal reason.

Another issue is a possibility to get an ethical clearance from The Ministry of Women, Family and Community Development. Elderly are the vulnerable population, so the process of getting permission to conduct a study in the institutionalized care may take a longer time. A thorough review from the ethical committee member by the ministry may delay the process of data collection.

1.8 Summary

The prevalence of elderly with diabetes in recent years has increased. Diabetes foot complication gaves significant impact to the patients. However, majority of them does not comply to perform good diabetes foot behaviour. Apart from that, some of the elderly in Malaysia who stayed in institutionalized care reported has diabetes. As elderly become older, a special care need to be given to them in order to improve health status.

Chapter 1 has outlined the background and problem statements as well as the significance of this study, presenting its aims, objectives, research questions and operational definitions.

Chapter 2

LITERATURE REVIEW

2.1 Introduction

Literature review is an important chapter in research, as it helps the researcher to gain a better understanding of the research topic. It supports the researcher's aim in exploring the necessary aspects to be analysed and supports the researcher to be more objective. Therefore, in this chapter, the review focused in depth and will be supported with the current literatures related to self-care behaviour and self-efficacy of elderly with diabetes. At the end of it, the conceptual framework is established in order to guide and identifying the problems that is needed to be solved accordingly.

2.2 Self-care behaviour of diabetes

Diabetes is a chronic disease and requires long term in self-care (Potter & Perry, 2010). Management of diabetes includes assessment of signs and symptoms, medication and lifestyle issues (Kozier et al., 2004). Complication of diabetes is preventable by simple measures that can largely be taken by the patient himself (Singh et al., 2013). Diabetes is rarely managed successfully with medication alone, its management usually require major changes in behaviour (Maddux, 1995).

Diabetes is a self-managed disease because a patient provides at least 99% of their own care (Feinglos & Bethel, 2008). Self-care behaviour is an essential element of diabetes care (McCollum et al., 2005). Self-care at an individual level can be defined as a comprehensive, explicit and instructive of personal behavioural changes (Webber et al., 2013). It includes the ability, knowledge, skills and confidence to make daily decisions, select and make behaviour changes and cope with the emotional aspects of their disease within the context of their lives (Barlow et al., 2002). It is possible to implement intervention which promote the self-care behaviour among diabetes patients (Freitas et al., 2014). For example, Malaysian Diabetes Association (2013) suggested diabetes patients needs to perform diet control, compliance to medication, stress on foot and skin care, routine eye check-up, avoid consume alcohol and smoking and exercise regularly for an ideal weight. Health education and transmission of health behaviour can improve health status of elderly with diabetes (Marzeih et al., 2014). Patients who are implementing good diabetes self-care behaviour will have better quality of life (Huang & Hung, 2007; Saleh et al., 2014). Contributions of better quality of life of patients with diabetes are obtained through knowledge of diabetes, together with social support (Misra & Lager, 2009).

However, self-care behaviour among people with diabetes has been found to be low, especially when looking at long-term changes (Shrivastava, Shrivastava, & Ramasamy, 2013). People may know and understand the importance of high self-care behaviour but having knowledge does not always translate to behaviour change (Letchuman et al., 2010). Knowledge alone was insufficient to explain increased understanding of diabetes care into the increased confidence and motivation necessary to improve one's diabetes self-care behaviour (Nam, Chesla, Stotts, Kroon, & Janson, 2011). Patients with diabetes need to make extensive changes in self-care behaviour adjustments may be accompanied by frustration and distress (Brown et al., 2004; Weinger 2007; Shu-Fang et al., 2011). Some of physical and psychosocial factors (beliefs, behaviour, self-confident, depression, social support, coping style, and personality) can influence self-care behaviour of people with diabetes to manage the disease effectively (Johnston-Brooks et al., 2002; West & Goldberg, 2002; Wang & Shiu 2004; Leichter, 2005; Heisler et al., 2005; Wu et al., 2007; Funnell et al., 2007; Huang & Hung, 2007; Munshi & Lipsitz, 2007; Cai-Xia et al., 2008; Tan & Magarey, 2008; Bai et al., 2009; Lee et al., 2009; Gallegos et al., 2009; Daly et al., 2009; Lee et al., 2009; Ooi et al., 2011; Raingruber, 2014; Satoko et al., 2014). Therefore, support from the health management leads to greater compliance to recommended behavioural modification (Misra & Lager, 2009; Ana et al., 2012; Freitas et al., 2014; Kargar et al., 2014).

Diabetes is complex disease to manage and require rigorous efforts at the population level as well as at the individual level (Mustapha et al., 2014). However, self-care behaviour among people with diabetes have been ignored by health provider (Mosnier-Pudar et al., 2010). Therefore, the stakeholders have a responsibility to overcome this problem (Letchuman et al., 2010). The greatest professional responsibility of a diabetes educator is to build patients' confidence and competence in their ability to self-care their diabetes independently (Rodriguez, 2013). The education session for patients with diabetes need to be revised and educating skills among the diabetic educators is important to be discussed on top (Azimah et al., 2009). The challenges among the health care professionals is to seek creative ways to deliver diabetes education (Wilkinson, Whitehead, & Ritchie, 2014). Elderly must be empowered with knowledge, behaviour, confidence and skills to perform self-care behaviour (Ambigga et al., 2011).

2.2.1 Foot self-care behaviour

Foot self-care behaviour education programs can reduce the incidence of foot related to diabetes problems (American College of Foot and Ankle Surgeons, 2006; Singh, Pai & Yuhhui, 2013). The following foot self-care behaviour: drying between the toes after shower, maintaining the feet free of humidity, and performing daily inspection of the lower limbs, use of closed and comfortable shoes and nail must be trim correctly to prevent injury (Malaysian Diabetes Association, 2013). This suggest for better protection which can lead to the lesser of pain and physical sensitivities of the feet, hence, preventing the incidence of injury on feet (Freitas et al., 2014). Lee et al. (2009) stated patients who had received education about diabetic foot care were significantly more likely to examine their feet regularly.

However, foot self-care behaviour, which requires inspecting feet thoroughly to check for any abrasions, lesions and early infections may be thought of as a relatively solitary activity (Ciechanowski et al., 2004). Foot self-care behaviours in patients with diabetes are below practiced (Chin & Huang, 2013) and more than half of their respondents do not compliance to perform foot self-care behaviour (Saleh, Mumu, Ara, Hafez, & Ali, 2014). It may due to that elderly with diabetes are having various problem such as memory problems, impaired cognitive function, and deficient awareness regarding to administer foot self-care behaviour (Saleh et al., 2014).

Therefore, it is necessary to pay special attention to this issue when teaching or counselling on foot self-care behaviour among patients with diabetes (Singh et al., 2013). The availability of a trained nurse counsellor to engage and counsel patients may help in preventing unnecessary delays in patients with diabetic foot ulcer (Ang & Lim, 2013).

2.3 Health educational programs to improve foot self-care behaviour and foot problems among the elderly with diabetes: a systematic review

2.3.1 Introduction

The complications of diabetes among the elderly are a major health concern. As diabetes becomes a global issue, many public health concerns are interested to overcome the problem (Hu, 2011). Foot problems such as neuropathy, ulcer, and ultimately amputation are a great burden on the older people with diabetes. Diabetes education focused on decision-making and self-care were able to improve health outcome (Funnell et al., 2010). Diabetes foot education program can influence the behaviour of the elderly in practicing foot self-care and controlling the foot problems. However, the educational approaches used by the educators were difference. To the best of our knowledge, no published data exists on a systematic review regarding diabetes foot education program among the elderly population. Therefore, related articles were systematically reviewed to gather related intervention studies, as a guide that the educational programs can improve foot self-care and foot problems of elderly with diabetes. The aims of the review was to assess the health educational programs to improve foot self-care and foot problems among the elderly with diabetes.

2. 3.2 Methods

Previous research articles about educational programs for improving foot self-care and foot problems among the elderly with diabetes were thoroughly searched and reviewed. The primary desired outcome was the improvement in diabetes foot self-care. In this review the diabetes foot self-care of elderly is about their health behaviour in performing foot care towards themselves. The secondary outcome in this review was improvement in foot-related health, such as the absence of diabetes foot complications.

2.3.3 Data Sources and search strategy

The literature review was conducted with the following search engines and databases: EBSCOhost medical collections (MEDLINE, CINAHL, Psychology and Behavioral Sciences Collection) SAGE, Wiley Online Library, ScienceDirect, SringerLink, and Web of Science. Other databases were search to compare between sources of data. The medical subject heading was adapted from search methods used by Dorresteijn et al., (2010). Keywords included 'diabetes', combined with 'foot' and 'self-care' and multiple search terms. The references and citation from the articles were searched for other potentially eligible studies and to obtain for related information, per Table 2.1. The search was limited to the full text research article, English Language, and studies published from January 2000 to the recent year. The articles were limited to nursing, health care, medical journals, and health education journals.

Table 2.1 Search strategy used in databases

Dates 2000 to March 2015
1. Foot
2. Feet
3. Foot ulcer
4. Foot disability
5. Foot problem
6. Neuropathy
7. (or/1-6)
8. Care
9. Self-care
10. Self-care behaviour
11. Self-management
12. Self-practice
13. Self-care practice
14. (or/8-13)
15. Diabetes
16. Diabetes mellitus
17. (or/15-16)
18. (7 and 14 and 17)
19. Limit to English language, full text research article and average age 60 years or above

The inclusion criteria were

Type of study

- Randomized Control Trials and quasi-experimental intervention study
- Education program in relation of diabetes foot self-care
- Program involved with or without control group

Types of participants:

- Average aged 60 years or above

Types of intervention

- Educational programs which included teaching, coaching, discussion, demonstration and assessment
- The programs conducted by medical personnel (diabetic nurse educator, dietician, psychologist, occupational therapist, physician, and/or podiatrist)

Types of outcome measures

- Primary outcome: diabetes foot self-care
- (e.g. inspection, hygiene, appropriate socks and shoe, nail care, professional treatment)
- Secondary outcome: Foot problems
- (e.g. neuropathy, lesion, ulcer, amputation, foot disability, callus, tinea pedis)

The studies were excluded if:

- Studies related to diagnostic instruments, clinical and pharmacological trials, other design (such as qualitative study, non-experimental, systematic review and meta-analysis and case reports)
- Papers focusing on elderly with other chronic diseases such as arthritis, renal failure and hypertension
- Participants involved diabetic elderly with dementia, mental illness, or any cognitive problems
- Participants among adult or young population with diabetes
- Participants among health care providers
- Papers written in other languages (due to limited resources for translation process)

2.3.4 Search outcome

The searching process was conducted electronically according to the Preferred Reporting Items for Systematic reviews and Meta-Analyses (PRISMA) guidelines (2009) (Moher et al., 2009). Figure 2.1 describes the flow diagram for the process of study selection.

Initially, 616 articles were identified from six databases (Figure 2.1). The search process involved assessment at the title, followed to the abstract. If title and abstract were not related to the study objective, the articles were excluded from further review. At this stage, the most common articles were excluded from several reasons; review paper, dissertation and thesis, conference proceeding and abstract.

After process of screening and looking for eligible articles based on inclusion criteria, only 31 articles were examined. The assessments were made in terms of the article content which included introduction, methodology, result, discussion and conclusion. Only experimental designs comparing different strategies in health education to improve foot self-care and foot problems among the elderly with diabetes were included. All related research articles involving respondent at average aged of 60 years or above only were reviewed. A total of 17 articles were rejected due to the following reasons: protocol development, not experimental study, participants' average aged less than 60 years and analysis of finding combined overall diabetes self-care.

Any program related to diabetes foot self-care alone or combined with other behaviour changes (diet, exercise, blood glucose checking, and medication intake) were reviewed if the analyses in these results were conducted separately. The educational programs may include; teaching delivery method, one-to-one or by group approached, conducted either in health setting or at patient's home, with or without control group, follow-up session and evaluation as well as key findings. The findings

searched included improvement of diabetes foot self-care and foot problems. Figure 2.1 describes the flow diagram of process of search strategy.

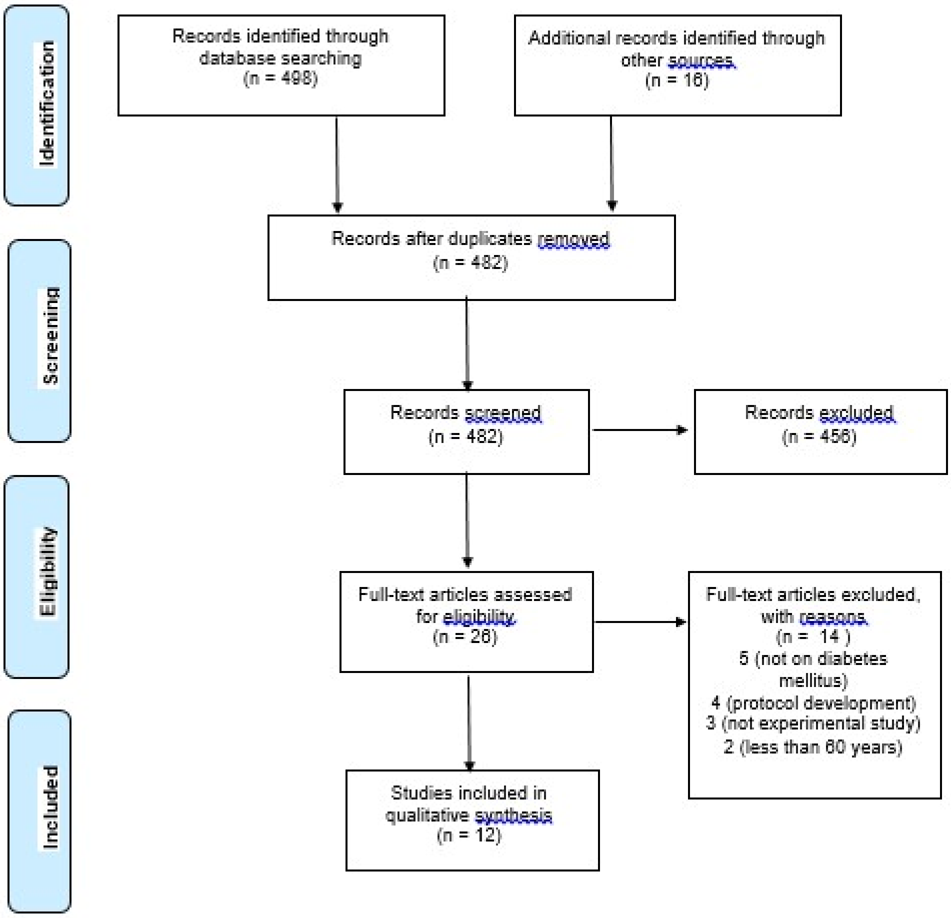

Figure 2.1 PRISMA 2009 Flow Diagram for the process of study selection

2.3.5 Data extraction process and quality assessment

The related articles were reviewed based on the following objectives: foot self-care combined with/without foot problems among diabetes population, application of theory (if any), the design, the program, follow-up and evaluation, outcome measures, and key findings (Table 2.2).

The Consolidated Standards of Reporting Trials (CONSORT) statement for assessing non-pharmacologic treatments checklist was used to assess the quality of articles (Boutron et al., 2008a; Boutron et al., 2008b). The articles were classified as good, moderate, or poor rate according to six components: selection bias, design, confounders, blinding, data collection methods, and withdrawals and dropouts (Thomas et al., 2004). The articles were rated as follows: good (at least four strong ratings); moderate (less than four strong ratings and one weak rating) and weak; (two or more weak ratings) (Thomas et al., 2004). However, the weak articles were not excluded, as the information provides new knowledge and important ideas. Table 2.2 showed the literature summary for the education programs to improve foot self-care behaviour and foot problems among the elderly with diabetes.

2.3.6 Findings and discussion

A total of 14 articles were included in the final synthesis (Table 2.2). Initially, older people at the age of 60 years or above were filtered, but not all the articles mentioned in detail the age of the participants. Following that, information about age of the participants was gathered from the result of each studies identified. The mean age of respondents at 60 years and above were then included and analysed.

The design used from the articles was experimentally based. Three articles were randomized control trials (Lincoln et al., 2008; Waxman et al., 2003; Borges & Ostwald, 2008) and eleven articles were of quasi-experimental design (Baba et al., 2014; Nadia et al., 2012; Fujiwara et .al., 2011; Chen et al., 2011; Calle-Pascual et al., 2002; Aikens et al., 2015; Williams et al., 2014; Tang et al., 2012; Ko et al., 2011; Deakin, et al., 2006; Dettori et al., 2005).

The studies were conducted in many countries. Five studies were conducted in the U.S. (Borges & Ostwald, 2008; Aikens et al., 2015; Williams et al., 2014; Tang et al., 2012; Dettori et al., 2005). Three studies were conducted in the U.K. (Lincoln et al., 2008; Deakin et al., 2006; Waxman et al., 2003). Whereas, one study was conducted in Taiwan (Chen et al., 2011), Japan (Fujiwara et al., 2011), Australia (Baba et al., 2014), Egypt (Nadia et al., 2012), Spain (Calle-Pascual et al., 2002), and Korea (Ko et al., 2011).

The main aim of the diabetes education program was focused on foot care. There were eight studies concentrated specially on diabetes foot self-care education programs (Lincoln et al., 2008; Nadia et al., 2012; Borges & Ostwald, 2008; Baba et al., 2014; Fujiwara et .al., 2011; Waxman et al., 2003; Chen et al., 2011; Calle-Pascual et al., 2002). Six studies were conducted on general measures of diabetes self-care education program which included foot care aspect (Aikens et al., 2015; Williams et al., 2014; Tang et al., 2012; Ko et al., 2011; Deakin et al., 2006; Dettori et al., 2005). General diabetes education includes all aspect of diabetes care such as diet control, medication adherence, exercise, blood glucose monitoring and smoking cessation. However, educators may provide less information on foot care. The diabetes educator who is interested in the diabetic foot may deliver a special training focused on the management of diabetic foot conditions (Mayfield et al., 2004).

All the education programs were delivered by trained personnel in the research field who has medical and health sciences backgrounds such as nurse, diabetic educator, dietician, psychologist, occupational therapist, physician, and/or podiatrist (Lincoln et al., 2008; Nadia et al., 2012; Borges & Ostwald, 2008; Baba et al., 2014; Fujiwara et .al., 2011; Waxman et al., 2003; Chen et al., 2011; Calle-Pascual et al., 2002; Aikens et al., 2015; Williams et al., 2014; Tang et al., 2012; Ko et al., 2011; Deakin et al., 2006; Dettori et al., 2005). The advantage of this strategy is to prevent observer bias and give quality of information to the older people. However, the person delivering the program may be from a different background, although most of the providers were medical and health sciences certified.

Application of theory may guide the researcher to improve clinical practice by providing a holistic and comprehensive education program. Researchers may apply various strategies to enhance patient compliance by targeting multiple approach, goal setting, problem solving, self-monitoring and family support. In this review only two studies used theory in their research. There were Self-efficacy (Borges & Ostwald, 2008) and Social Cognitive Theory (Williams et al., 2014). Meanwhile, other studies did not state the model used in their research (Lincoln et al., 2008; Waxman et al., 2003; Baba et al., 2014; Nadia et al., 2012; Fujiwara et .al., 2011; Chen et al., 2011; Calle-Pascual et al., 2002; Aikens et al., 2015; Tang et al., 2012; Ko et al., 2011; Deakin et al., 2006; Dettori et al., 2005).

The methods of education delivered by the researchers were varied. There were thirteen studies that provided the information through traditional methods (teaching, demonstration, discussion) (Borges & Ostwald, 2008; Lincoln et al., 2008; Waxman et al., 2003; Baba et al., 2014; Nadia et al., 2012; Fujiwara et .al., 2011; Chen et al., 2011; Calle-Pascual et al., 2002; Williams et al., 2014; Tang et al., 2012; Ko et al., 2011; Deakin et al., 2006; Dettori et al., 2005). There was one study from the U.S. which had implemented technologies in telecommunication; mobile health interactive voice response (Aikens et al., 2015).

Nine studies were conducted in health setting such as in diabetic clinics, non-emergency departments, community health clinics, and podiatry clinics (Borges & Ostwald, 2008; Waxman et al., 2003; Baba et al., 2014; Nadia et al., 2012; Fujiwara et .al., 2011; Calle-Pascual et al., 2002; Williams et al., 2014; Tang et al., 2012; Deakin et al., 2006). Two studies were conducted in participants' homes (Lincoln et al., 2008; Ko et al., 2011). There were also studies conducted both in health setting and participant's home (Chen et al., 2011; Dettori et al., 2005) by means after the participants received the education at health setting, there were subsequent followed up visit at their home. Only one study was conducted in a health setting followed by contacting the participants via telecommunication (Aikens et al., 2015).

There were six studies conducted in a group (Borges & Ostwald, 2008; Waxman et al., 2003; Baba et al., 2014; Nadia et al., 2012; Williams et al., 2014; Tang et al., 2012). Four studies were conducted in one-to-one approach (Lincoln et al., 2008; Aikens et al., 2015; Ko et al., 2011; Deakin et al., 2006). Two studies were conducted both (group and one-to-one) (Chen et al., 2011; Dettori et al., 2005). Two studies did not mention whether the studies were conducted in a group or one-to-one (Fujiwara et al., 2011; Calle-Pascual et al., 2002).

The researchers had tried new strategies according to their objective and design that suits to the target population. It can be seen that these studies conducted in health setting, at patient's home and/ or by telecommunication. Teaching session was delivered in traditional methods or by telecommunication. Furthermore, the sessions were conducted in a group session or one to one. However, at the end of education program, there was improvement in foot self-care and foot problems among elderly with diabetes. The advantages of new design is it may help the diabetic elderly as well as the health provider to implement the most effective way in promoting foot self-care and foot problems. However, the disadvantages are when the new methods are costly or not feasible to be conducted by the educator or followed by the elderly.

Referring to the control group, there were two studies that received usual care for their control group (Borges & Ostwald, 2008; Waxman et al., 2003). The control group in Lincoln's study received the same leaflet regarding foot care as intervention group but not any teaching instruction (Lincoln et al., 2008). However, Baba's study did mention that group A received written education while group B received an interactive educator-led session (Baba et al., 2014). One study divided the sample into two groups (Low risk and high risk) but both group had received same education program (Calle-Pascual et al., 2002). In Nadia's study, nothing was not mentioned about any care received by the control group (Nadia et al., 2012). Eight studies only involved one group pre-test and post-test (Fujiwara et .al., 2011; Aikens et al., 2015; Chen et al., 2011; Williams et al., 2014; Tang et al., 2012; Ko et al., 2011; Deakin et al., 2006; Dettori et al., 2005).

Time of follow-up and evaluation were varied across the studies. Seven studies were measured at the baseline (Time 1) and evaluation (Time 2) (Baba et al., 2014; Fujiwara et .al., 2011; Waxman et al., 2003; Borges & Ostwald, 2008; Chen et al., 2011; Ko et al., 2011; Dettori et al., 2005). Another six studies were measured at baseline (Time 1), follow-up (Time 2) and evaluation (Time 3) (Aikens et al., 2015; Lincoln et al., 2008; Calle-Pascual et al., 2002; Williams et al., 2014; Tang et al., 2012; Deakin et al., 2006). One study was measured at baseline (Time 1), immediately after the program (Time 2), follow-up (Time 3), and evaluation (Time 4) (Nadia et al., 2012).

There were also a significant variation in the interval time of follow-up and evaluation among the studies. The researchers were evaluated the program differently between at baseline (Time 1) and after (Time 2). The program was evaluated at 1 month (Borges & Ostwald, 2008), at 3 months (Baba et al., 2014; at 6 months (Waxman et al., 2003; Chen et al., 2011), at 8 months (Ko et al., 2011), at 2 years (Fujiwara et al., 2011) and at 2 – 3 years (Dettori et al., 2005). Nadia's study were measured at baseline (Time 1), immediately post (Time 2), 3 months (Time 3), 6 months (Time 4) (Nadia et al., 2012). Other studies were measured at: baseline (Time 1), 3 months (Time 2), 6 months (Time 3) (Aikens et al., 2015); baseline (Time 1), 3 months (Time 2), 12 months (Time 3) (Williams et al., 2014); baseline (Time 1), 4 months (Time 2), 14 months (Time 3) (Deakin et al., 2006); baseline (Time 1), 6 months (Time 2), 12 months (Time 3) (Lincoln et al., 2008); baseline (Time 1), 6 months (Time 2), 30 months (Time 3) (Tang et al., 2012); baseline (Time 1), 6 months (Time 2), 3 to 6 years (Time 3) (Calle-Pascual et al., 2002).

In this review, some studies were involved more than one group in their program. Some of the control group were received usual care or received same leaflet as intervention group. Furthermore, some of studies were had different interval time of follow-up and evaluation depending on the study objective and strategies. This may influence the impact of the programs. Another issue was bias, as it would affect the methods and outcome due to different approaches especially when the programs involves multiple strategies such as discussion, counselling, motivation, support, video session and demonstration. The educator may be more motivated to deliver the program to the intervention group. However, in one group study, the researchers were only able to assess the effects between before and after the program was delivered.

Outcome measured in these articles were included foot self-care (e.g. inspection, hygiene, appropriate socks and shoe, nail care, professional treatment), self-efficacy, empowerment, problem solving, knowledge, acculturation, medication adherence, social support, emotional, anxiety and depression, quality of life, diabetes health care utilization, satisfaction with care. The secondary outcome included clinical findings or signs and symptoms of foot problems (e.g. assessed for peripheral neurologic assessment, vibration perception threshold, pain and disability, morbidity, ulcer, lesion, amputation, tinea pedis and callus). Other significant outcomes in relation with diabetes also were measured such as Self-Monitoring Blood Glucose (SMBG), Fasting Blood Sugar (FBS), Glycoslated haemoglobin (HbA1c), lipid profile, blood pressure, Body Mass Index (BMI), waist circumference, retinal examination and urinalysis.

The instruments used varied across these studies. Most of the researcher used a questionnaire as their research tool. The self-reported questionnaire may have validity issues, less precise and misclassification. Different instrument used to measure the same outcome may influence the outcome. For example, there were studies which measured the adherence of diabetes foot self-care with “Nottingham Assessment of Functional Footcare (NAFF)” by Lincoln et al., (2007) (Lincoln et al., 2008; Baba et al., 2014). While in other studies the similar tool was developed by the researchers itself (Chen et al., 2011; Nadia et al., 2012). Another example was for the clinical findings for incidence of foot problems. Chen’s study used the Michigan Neuropathy Screening Instrument developed by Moghtaderi et al. (2006) (Chen et al., 2011). However, in another study, the foot problems were identified from the subject’s medical records (Lincoln et al., 2008). It would be happened as different settings have different research protocols and practice guidelines.

Key findings showed improvement in both foot self-care scores and incidence of foot problems (neuropathy and ulcers) (Chen et al., 2011; Calle-Pascual et al., 2002). There was an improvement in the foot self-care scores only (Lincoln et al., 2008; Borges & Ostwald, 2008; Nadia et al., 2012; Aikens et al., 2015; Williams et al., 2014; Tang et al., 2012; Ko et al., 2011; Deakin et al., 2006; Dettori et al., 2005). Lastly, the incidence of foot problems improved in terms of lower foot score (Baba et al., 2014), lower foot disability score, treatment session and lesion (Waxman et al., 2003), as well as reduction in the severity of tinea pedis and callus (Fujiwara et .al., 2011).

In summary, all education programs implemented by the previous researchers were varied according to design, setting, approach, outcome measured and result. Foot assessment, verbal and written instruction and discussion were proven to improve the foot self-care and foot problems. Subsequent follow-ups and evaluations were given a significant effect. The main results showed an improvement in foot self-care score and foot problems (such as neuropathy, foot disability, lesion, ulcer, tinea pedis, callus grade) after implementation of the health education program.

The findings support the claim that the education program increased the foot self-care scores and reduced foot problems. However, there were certain methodological concerns in the reviewed articles in doubt, proving the need for further evaluation. In future, researchers and practitioners must conduct a vigorous education program focusing on diabetes foot self-care among the elderly population.

Table 2.2 Literature summary for the education programs to improve foot self-care behaviour and foot problems among the elderly with diabetes

**Author Design Country**	**Title Theory (if any)**	**Objective Sample**	**Education program method Intervention group (IG) Control group (CG)**	**Follow-up and outcome measure**	**Primary outcome (PO) Secondary outcome (SO)**	**Conclusion**	**Quality of method**
Borges & Ostwald (2008)<br><br>3-arm RCTs<br><br>The U.S	Improving foot self-care behaviors with Pies Sanos<br><br>Theory: Self-efficacy	To improve diabetes self-efficacy and foot self-care behaviors in adult patients with type 2 diabetes who lived in a predominantly Mexican American community<br><br>N= 55 (IG1), 55 (IG2) 57 (CG)	Approach: Group, health setting<br><br>IG1 (risk assessment group): Time: 5-min foot risk assessment using a monofilament<br>IG2 (intervention group): Time: 5-min foot risk assessment using a monofilament and a 15-min brief foot self-care intervention.<br>Content: The educational and behavioral strategies designed to increase self-efficacy for foot self-care and ultimately change foot self-care behaviors. Pictorial form, “Foot Care for People With Diabetes”<br>CG: usual care<br>Delivered by the researchers	Baseline - 1 month<br><br>Outcome:<br>Knowledge<br>Self-efficacy<br>Self-care<br>Acculturation<br>Foot self-care observation	PO:<br>There was a significant difference in foot self-care behaviors between groups ($p < 0.05$)<br><br>Improved foot self-care behaviors within the CG, ($p <0.05$) and the IG2 ($p <0.01$)<br><br>SO: not mentioned	The intervention improved diabetes foot self-care behaviours	Good
Lincoln, Radford, Game, & Jeffcoate, (2008)<br><br>RCT<br><br>The U.K	Education for secondary prevention of foot ulcers in people with diabetes: a randomised controlled trial<br><br>Theory: not mentioned	To determine the effect of a foot care education programme in the secondary prevention of foot ulcers<br><br>N=87 (IG), 85 (CG)	Approach: one-to-one, home-visit<br><br>IG:<br>Time: 1 hour<br>Content: causes of foot ulcers, promoting foot health, daily self-examination and contact the clinic. Illustration/picture and leaflets.<br>Phone call after 4 weeks<br>CG: usual care group with leaflets<br><br>Delivered by diabetes research nurse specialist or a research occupational therapist	Baseline - 6 months - 12 months.<br><br>Outcome:<br>Anxiety and depression<br>Quality of life<br>Foot care behaviours<br><br>Ulcer incidence<br>Incidence of amputation	PO:<br>Foot care behaviours at 12 months were better in the IG than in the CG ($p=0.03$)<br><br>SO:<br>No significant differences ($p> 0.05$) were observed between groups in ulcer and amputation incidence at either 6 months or 12 months.	The intervention was associated with improved foot care behaviour.	Good

Author Design Country	Title Theory (if any)	Objective Sample	Education program Intervention group (IG) Control group (CG)	Follow-up and outcome measure	Primary outcome (PO) Secondary outcome (SO)	Conclusion	Quality of method
Waxman et al., (2003)<br><br>RCT<br><br>The U. K	FOOTSTEP: a randomized controlled trial investigating the clinical and cost effectiveness of a patient self-management program for basic foot care in the elderly<br>Theory: not mentioned	To evaluate the clinical and cost-effectiveness of a self-management program as a means of managing non-urgent demands for podiatry services by the elderly without compromising foot-related disability<br><br>N=78 (IG), 75 (CG)	Approach: group, health setting<br><br>IG:<br>Time: 1 hour<br>Content: Hygiene, footwear and hosiery. Foot-care packs. video and handbook<br><br>Telephone help 24 hours/ day<br><br>CG: Subjects are advised to seek routine foot care at a minimum of every 3 months, or earlier if specific problems arise.<br><br>Delivered by podiatrist	Baseline - 6 months<br><br>Outcome:<br>Foot pain and disability<br>Foot morbidity<br>Knowledge<br>Empowerment	PO: not mentioned<br><br>SO:<br>IG had lower foot disability scores than the CG (-1, 95% C.I. -2, 0)<br>Treatment sessions (92 CG, 39 IG),<br>Lesions (96 CG, 28 IG)	The results of the FOOTSTEP project support a more patient-led approach to care, where the patient is empowered to determine their own treatment requirements	Good
Baba, Duff, Foley, Davis, & Davis, (2014)<br>Quasi-experimental<br><br>Australia	A comparison of two methods of foot health education: The Fremantle Diabetes Study Phase II<br><br>Theory: not mentioned	To compare the effectiveness of two different methods of education on foot health, behaviours and attitudes in patients with type 2 diabetes.<br><br>N=154<br><br>78 (G1), 76(G2)	Approach: G2 (group), G1 (none), health setting<br><br>(G1) Community-based patients were allocated to written education. A detailed information booklet entitled “My feet and diabetes” (foot care information, foot care activities, footwear and selection and foot care tools)<br>(G2) A group of 10–15 people. The interactive Foot-smart education program (foot facts, diabetes complications, how diabetes affects your feet, how to care for your feet, how to choose a shoe, how to check your feet and problems to look for. No written information. Time: 90 min.<br><br>Delivered by diabetes educator	Baseline - 3 months<br><br>Outcome:<br>Foot care behaviour<br>Foot care related worries in diabetes<br><br>Foot Score (severity of podiatric disorders)	PO: G2 had better understood how to prevent foot complications than G1 after education ($P = 0.031$).<br>Foot care behaviour showed no significant difference between groups ($p=0.13$)<br>SO: There was a greater change in Foot Score from baseline to 3 months in G1 vs G2 ($P < 0.001$)	Written information was more effective at improving foot health while interactive education improved confidence in undertaking preventive measures.	Good

Author Design Country	Title Theory (if any)	Objective Sample	Education program Intervention group (IG) Control group (CG)	Follow-up and outcome measure	Primary outcome (PO) Secondary outcome (SO)	Conclusion	Quality of method
Nadia M Saleh, Shebl, Hatata, & Refiei, (2012)<br><br>Quasi experiment al<br><br>Egypt	Impact of educational program about foot care on knowledge and self-care practice for diabetic older adult patients<br><br>Theory: not mentioned	To determine the impact of foot care educational program on knowledge and foot self-care practice for diabetic elderly patients<br><br>N=60 (IG), 100 (CG)	Approach: group, health setting<br><br>IG:<br>Time: 30 minutes/session<br><br>8 sessions = 2 sessions per week<br>(3 educational and 5 practical / training sessions) program was implemented over 4 weeks period.<br><br>Content: Diabetic foot, importance of foot care and foot self-examination, foot care, proper foot wear, importance and type of exercise.<br>Instructional media and booklet, foot care bags<br><br>CG: not mentioned<br>Delivered by the researcher	Baseline - immediately - 3 months - 6 months<br><br>Outcome:<br>Knowledge<br>Foot self-care practice	PO:<br>The feet self-care practice IG before (mean=12.47±5.68) and after (mean=43.17 ±3.89) program was improved. However, there was not statistically significant difference found between the groups ($p>0.05$)<br><br>SO: not mentioned	The educational program reported higher knowledge and practice score	Weak
Fujiwara et .al., (2011)<br><br>Quasi-experiment al<br><br>Japan	Beneficial effects of foot care nursing for people with diabetes mellitus: an uncontrolled before and after intervention study<br>Theory: not mentioned	To assess the effectiveness of a preventative foot care nursing programme for diabetic patients<br><br>N = 88	Approach: not mentioned (one-to-one or group), health setting<br><br>Low risk: once/ year<br>High risk: G1 (every 6 months), G2 (every 3 months), G3 (every 1-3 months)<br><br>Time: 30-60 minutes<br><br>Content: Inspection of the feet, hygiene, nail cutting, appropriate shoes, avoid possible minor injury developing into foot ulceration Education and demonstration.<br><br>Delivered by certified diabetic foot care nurse	Baseline - 2 years<br><br>Outcome:<br>Foot-ulcer<br>Callus	PO: not mentioned<br><br>SO:<br>Reduced the severity of tinea pedis ($p < 0.001$)<br>Improved callus grade ($P < 0.001$).	A nurse-based foot care programme is effective in preventing diabetic foot in diabetic patients	Moderate

Author<br>Design<br>Country	Title<br>Theory<br>(if any)	Objective<br>Sample	Education program<br>Intervention group (IG)<br>Control group (CG)	Follow-up and outcome measure	Primary outcome (PO)<br>Secondary outcome (SO)	Conclusion	Quality of method
Chen et al., (2011)<br><br>Quasi-experimental<br><br>Taiwan	Effectiveness of a health promotion programme for farmers and fishermen with type-2 diabetes in Taiwan<br><br>Theory: not mentioned	To examine the diabetes control and foot self-care capability in farmers and fishermen following introduction of a multi-stage, multi-disciplinary team and community-based small group health promotion programme<br><br>N=323	Approach: group, one-to-one, health setting and home<br><br>Content:<br>1) personal health assessment (foot ulcer, clinically significant neuropathy and vasculopathy, and poor diabetic control) and community-based small group health promotion education (diet control, medication compliance, foot care, physical activity); followed by 2) individually tailored home phone counselling was given 1–3 times per person for about 15–30 minutes<br><br>Delivered by diabetes educators and public health nurses, metabolic physicians and plastic surgeons.	Baseline - 6 months<br><br>Outcome:<br>Peripheral neurological assessment.<br>Peripheral vascular assessment<br>Foot self-care capability<br><br>HbA1c, BMI, Waist circumference (WC),	PO: Foot self-care behaviour improved significantly after the program ($p < 0.01$)<br><br>SO:<br>The neuropathy Screening Index and right side ankle brachial pressure index improved significantly ($p < 0.01$)	The health promotion programme benefits to many physiological indicators; peripheral neuronal and vascular functions	Good
Calle-Pascual et al., (2002)<br><br>Quasi-experimental<br><br>Spain	A preventative foot care programme for people with diabetes with different stages of neuropathy<br><br>Theory: not mentioned	To assess the efficacy of a preventative foot care programme, applied in a normal outpatient setting to decrease the incidence of foot ulcers in people with diabetes diagnosed as having neuropathy by neuropathy disability score (NDS), in relation to the severity of neuropathy based on the vibration perception threshold (VPT)<br>N = 308 (Low risk VPT = 124 (High risk VPT = 184)	Approach: not mentioned (one-to-one or group), health setting<br><br>Time: 120 minutes / week; 4 session for both group:<br><br>Content: assessment peripheral vascular disease, foot self-care, teaching on, shoes, socks, walking barefoot, hygiene, callus care, nail-cutting, temperature check, foot care products, foot and shoe inspection.<br>Monthly visit for 6 months<br><br>Delivered and treatment by podiatrist<br>Evaluated every six months (30 – 60 minutes) assessment by neurothesiometer	Baseline - 6 months - 3 to 6 years<br><br>Outcome:<br>Foot care behaviour<br><br>VPT value	PO:<br>All patients (71%) complied with the foot care behavior, compliance being 76% and 68% in low and high risk groups.<br><br>SO:<br>The low risk group developed nine ulcers in nine patients, and the high risk group 24 ulcers in 19 patients.	The programme reduces the incidence of foot ulceration in people with diabetes with neuropathy	Moderate

Author Design Country	Title Theory (if any)	Objective Sample	Education program Intervention group (IG) Control group (CG)	Follow-up and outcome measure	Primary outcome (PO) Secondary outcome (SO)	Conclusion	Quality of method
Aikens, Rosland, & Piette, (2015)<br><br>Quasi-experimental<br>The U. S	Improvements in illness self-management and psychological distress associated with telemonitoring support for adults with diabetes<br><br>Theory: not mentioned	To characterize changes in diabetes self-management and psychological distress associated with a mobile health (mHealth) interactive voice response (IVR) self-management support program.<br><br>N=301	Approach: one to one, health setting and by telecommunication<br><br>Time: 45 minutes<br><br>Subjects received weekly IVR calls assessing health status and self-care and providing tailored pre-recorded self-management support messages. The first wave receiving IVR calls weekly for 3 months and the second wave receiving IVR calls weekly for 6 months.<br><br>Content/ program: patients received suggestions on self-management, medical check-up, support, and patients' clinicians were notified automatically when patients reported significant problems.<br><br>Delivered by the research team	Baseline - 3 months - 6 months<br><br>Outcome:<br>Quality of life<br>Medication adherence<br>Self-management (including foot care)<br>Social Support<br>Depressive symptom<br>Diabetes-related distress<br><br>SMBG<br>Blood pressure	PO:<br>There were significant improvements in foot self-management; patients' IVR-reported frequency of weekly checking feet ($p < 0.001$).<br><br>SO: not mentioned	The short term and long term self-management (including foot) were improved	Good
Williams et al., (2014)<br><br>Quasi-experimental<br><br>The U. S	Enhancing diabetes self-care among Rural African Americans with diabetes: results of a two-year culturally tailored intervention<br><br>Theory: Social cognitive theory (SCT)	To test the feasibility of conducting a community-based randomized controlled trial evaluating a culturally tailored community-based group diabetes self-management education (DSME) program among rural African Americans<br><br>N= 25	Approach: group, health setting<br><br>Time: 2 hours x 8 weekly sessions<br><br>Content/ program: videotaped stories, identify specific areas of need. Information with simple/culturally appropriate materials and learning activities, set an individual goal, make changes, involving a family/friend as a supporter for achieving the goal, and problem solving to overcome barriers to goal achievement.<br><br>Delivered by a certified diabetes educator (CDE) and nurse practitioner/case manager	Baseline - 3 months - 12 months<br><br>Outcome:<br>Knowledge<br>Diabetes empowerment (including foot care)<br>Diabetes problem-solving skills, Quality of life, Diabetes health care utilization<br><br>HbA1c, lipid profile	PO: An improvement of foot care: from baseline to 3 months post intervention 4.2 to 4.9 ($p = 0.013$) and at 12-month follow-up ($p = 0.001$)<br><br>SO: not mentioned	This research will help to inform clinicians and health policymakers as to the types of interventions that are feasible in a larger rural population	Moderate

Author<br>Design<br>Country	Title<br>Theory<br>(if any)	Objective<br>Sample	Education program<br>Intervention group (IG)<br>Control group (CG)	Follow-up and outcome measure	Primary outcome (PO)<br>Secondary outcome (SO)	Conclusion	Quality of method
Tang, Funnell, Noorulla, Oh, & Brown, (2012)<br><br>Quasi-experimental<br>The U. S	Sustaining short-term improvements over the long-term: Results from a 2-year diabetes self-management support (DSMS) intervention<br><br>Theory: not mentioned	This study examined the long-term impact of a 24-month, empowerment-based diabetes self-management support (DSMS) intervention on sustaining health-gains achieved from previous diabetes self-management education (DSME).<br><br>N = 89	Approach: group, health setting<br><br>Time: 75-min x 88 weekly sessions (a period of 24-months)<br><br>Program: reflect on relevant self-management challenges or experiences, recognize emotions associated with those experiences, engage in group-based problem-solving, and ask questions about diabetes and its care, and set behavioral goals and make action plans to achieve those goals<br><br>Delivered by nurse certified diabetes educator and a clinical psychologist.	Baseline – 6 months – 30 months<br><br>Outcome:<br>Quality of life,<br>Self-care behaviour (including foot care)<br>Diabetes empowerment<br><br>HbA1C, lipid panel, blood pressures<br>BMI	PO:<br>Post 6-month DSME, subjects' demonstrated significant improvements for foot exams ($p < 0.01$).<br><br>SO: not mentioned	The empowerment-based DSMS model can sustain or improve diabetes-related health (including foot)	Good
Ko, Lee, Kim, Kang, & Kim, (2011)<br><br>Quasi-experimental<br><br>Korea	Effects of Visiting Nurses' Individually Tailored Education for Low-Income Adult Diabetic Patients in Korea<br><br>Theory: not mentioned	To study the effects of individually tailored education by visiting nurses on diabetes knowledge, diabetes self-management, and blood glucose levels of low-income diabetic adult patients in Korea.<br><br>N = 96	Approach: one-to-one, home<br><br>Time: 60 – 90 minutes<br><br>Program/subject: Eight visits (1st visit: assessment 2nd to 7th visit: Individually tailored educational programs with booklet, ''Diabetes, 6th visit: Foot care, 8th visit: evaluation<br><br>Delivered by public health nurse	Baseline – 8 months<br><br>Outcome:<br>Knowledge<br>Self-management (including foot care)<br><br>FBS	PO:<br>After education, foot self-care management ($p < 0.001$) significantly improved.<br><br>The number of subjects who had not kept their feet clean decreased, whereas those who did not cut toenails on a regular basis decreased from 40 (41.7%) to 5 (5.2%).<br><br>SO: not mentioned	Tailored education effectively improved the patients' knowledge of diabetes and self-management (including foot self-care)	Good

Author<br>Design<br>Country	Title<br>Theory<br>(if any)	Objective<br>Sample	Education program<br>Intervention group (IG)<br>Control group (CG)	Follow-up and outcome measure	Primary outcome (PO)<br>Secondary outcome (SO)	Conclusion	Quality of method
Deakin, Cade, Williams, & Greenwood, (2006)<br><br>Quasi-experimental<br><br>The U. K	Structured patient education: the Diabetes X-PERT Programme makes a difference<br><br>Theory: not mentioned	To develop a patient-centred, group-based self-management programme (X-PERT), based on theories of empowerment and discovery learning, and to assess the effectiveness of the programme on clinical, lifestyle and psychosocial outcomes<br><br>N= 157 (IG), 157 (CG)	Approach: group, health setting<br><br>Time: 2 hours/ group for 6 sessions<br><br>IG:<br>The programme aimed to develop skills and build confidence, to enable patients to make informed decisions regarding their diabetes self-care. Delivered by dietician diabetes educator<br><br>CG: usual care and received diabetes education and review with prearranged individual appointments with a dietician (30 min), practice nurse (15 min) and general practitioner (10 min).	Baseline - 4 months - 14 months<br><br>Outcome:<br>Self-care (including foot care)<br>Quality of life<br>Diabetes empowerment<br>Medication intake<br><br>HbA1c, lipid, blood pressure, BMI,	PO:<br>At 4 months there was a significant difference in the number of days each week that the X-PERT patients performing foot care self-management (difference 0.7 day; 95% CI 0.4, 1.1)<br>The differences with respect to foot care remained significant at 14 months (difference 0.6 day, 95% CI 0.2, 1.0, respectively)<br><br>SO: not mentioned	Participation in the X-PERT programme was shown at 14 months to have led to improved self-management (including foot) skills	Good
Dettori et al., (2005)<br><br>Quasi-experimental<br><br>The U. S	Improvements in care and reduced self-management barriers among rural patients with diabetes<br><br>Theory: not mentioned	To improve diabetes care by establishing patient registries in local primary care practices, implementing targeted quality improvement interventions, and improving access to diabetes education services<br><br>N=213	Approach: one-to-one and group, health and home setting<br><br>Assessment and teaching by the Park County Diabetes Project and the Montana Diabetes Control Program. Organizing foot-care clinics, education materials; newsletter that described current, diabetes-related activities, diabetes self-management<br><br>Follow-up by phone call every year<br><br>Delivered by clinical staff and diabetes educators	Baseline – 2 to 3 years<br><br>Outcome:<br>Knowledge, (SMBG)<br>Satisfaction with care<br>Diabetes self-management (including foot care)<br><br>HbA1c, blood pressure, serum lipid, urinalysis, foot and dilated retinal examinations, immunization	PO:<br>Annual foot examination (43% to 58%, p= 0.002)<br><br>The proportion of respondents reporting foot care education (69% to 89%, p < 0.001)<br><br>SO: not mentioned	The diabetes education can improve care (including foot care) and reduce barriers for rural patients with diabetes on a countywide level.	Moderate

2.4 Health behaviour theories

Self-care is essential for diabetes management, however, it is complex and difficult to sustain. It may lead to noncompliance because the patient might have different attitudes, characteristic, and believes. Health behaviour change interventions for people with diabetes were most effective for increasing self-efficacy (MOH New Zealand, 2011). It is essentially valuable for nurses to use theory based program because it improves clinical practice by providing holistic and comprehensive care.

Theory can be defined as a set of ideas, meanings and propositions that present a systematic view of events by specifying relations among variables in order to explain and predict the events (Glanz, Rimer, & Viswanath, 2008). Theories are important to use as a framework as its help to plan, implement and evaluate the successful of a health promotion program.

There are theories to understand the health behaviour in health education. Many models focus on individual health behaviour such as The Health Belief Model, Theory of Reasoned Action, The Transtheoretical Model and The Precaution Adoption Process. There are also models focus on interpersonal health behaviour such as Social Networks and Social Support, The Transactional Model of Stress and Coping and Self-efficacy.

For example, The Health Belief Model has been one of the most widely used in health behaviour research. The model covers many construct; perceived susceptibility, perceived severity, perceived benefits, perceived barriers, cues to action and self-efficacy and other variables such as demographic data and socio-cultural. Even though the model recognises constructs that lead to outcome behaviors, relationships between and among these constructs are not defined and the emotional aspect does not considered (Glanz et al., 2008).

The Transtheoretical Model suggests six stages of change: precontemplation, contemplation, preparation, action, maintenance, and termination (Glanz et al., 2008). However, the ranks between the stages can be subjective with no set criteria of how to determine a person's stage of change and there is no clear sense for how much time is needed for each stage, or how long a person can remain in a stage.

Self-efficacy Theory in interpersonal health behaviour explained how interpersonal interactions may influence individual cognitions, beliefs and behaviours (Glanz et al., 2008). Self-efficacy has made two contributions to explanations of health-related behaviour that were previously not included in the Health Belief Model. The first is the emphasis on the several sources of information for acquiring expectations, particularly on the informative and motivational role of reinforcement and on the role of observational learning through modelling (imitating) the behaviour of others and the second major contribution is the introduction of the concept of self-efficacy (efficacy expectation) as distinct from outcome expectation (Rosenstock, Strecher, & Becker, 1988).

2.4.1 Self-efficacy; definition and description

The American psychologist, Albert Bandura in 1977, first introduced the concept of self-efficacy developed from social cognitive theory, formerly known as social learning theory (Bandura, 1994). By means that a central ideology of social cognitive theory is the concept of self-efficacy.

Self-efficacy defined as individuals' believes about their capabilities to produce designated levels of performance that activity influence over events that affect their lives (Bandura, 1994). Self-efficacy theory is a behaviour specific and dynamic, because it focuses on beliefs about individual abilities in a particular activities (Bandura, 1994; 1997; Wu et al., 2007). The patient's self-efficacy may affect behavioural choices and subsequent outcomes (Adam & Folds, 2014). This description shows that people's self-efficacy is not of a general nature, but related to a specific situations and easy to influence characteristic that is strictly situation-and task-related (Lenz & Shortridge-Baggett, 2002). For example; individuals can judge themselves as being very competent in a specific area and less competent in another area. It means that this concept emphasizes not individuals' skills, but their judgment of what they believe they can do. Judgment about the specific task built on past experiences will vary according to multiple factors such as strength and beliefs.

The basic premise underlying self-efficacy theory is that the expectations of personal mastery (self-efficacy or efficacy expectation) and success (outcome expectation) determine whether an individual will engage in particular behaviour (Figure 2.2). Self-efficacy (efficacy expectation) concerns the confidence in one's capability to produce the behaviour. An outcome expectation is a person's belief about the outcomes result from a given behaviour. These outcomes can take the form of physical, social and self-evaluative effects (Lenz & Shortridge-Baggett, 2002). The distinction between outcome and efficacy expectations is important because both are required for behaviour.

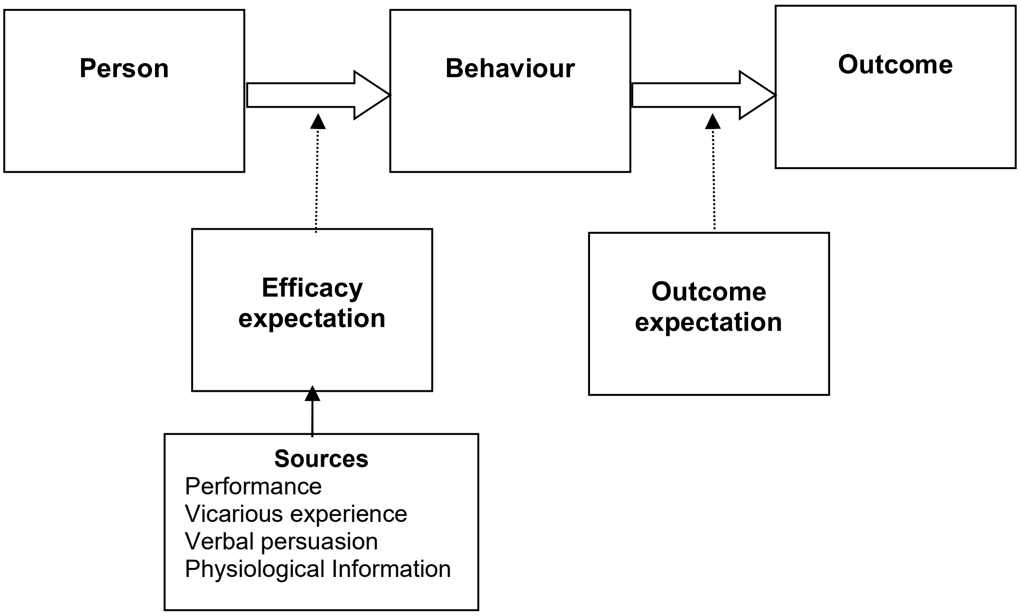

Figure 2.2 The relationship between efficacy expectation and outcome expectation source Bandura, (1977); Shortridge-Bagett & van der Bijl, (1996)

2.4.2 Source of self-efficacy (Lenz & Shortridge-Baggett, 2002)

Self-efficacy are influenced by four main important sources of information which are performance accomplishments: practicing and earlier experiences, vicarious experience: observation of others, verbal persuasion and physiological information: self-evaluation of physiological and emotional states.

Performance accomplishments: practicing and earlier experiences

Practicing is the most important source of self-efficacy because it based on a person's experience. Experiences of success (the feeling of mastery) enhance self-efficacy, while regular failure decreases self-efficacy, especially when the failure takes place early in learning process. Once a person has developed a strong self-efficacy, one failure does not have much influences.

Vicarious experience: observation of others

Seeing others perform successfully also is an important source of self-efficacy. Other persons can serve as example (role models) and supply information about the degree of difficulty of a specific kind of behaviour. The people serving as role models, however should show similarity to the observer in those characteristics which are relevant to the issue.

Verbal persuasion

Verbal persuasion is the most often used source of self-efficacy, because it is easy to use. By giving instructions, suggestion and advice, health care providers try to convince persons that they can succeed in a difficult task. For critical importance are the credibility, expertise, trustworthiness and prestige of the person doing the verbal persuasion.

Physiological information: self-evaluation of physiological and emotional states

Information on the human body can also influence a person's estimation of the capability to show a specific behaviour. In judging their own capacities persons use information about their physiological and emotional situations. They experience tension, anxiety and depression as signs of personal deficiency. In activities that require strength and perseverance, they interpret fatigue, pain, hypoglycaemia as indicators of low physical efficacy. Stress can have a negative influence on self-efficacy.

2.4.3 Methods for increasing self-efficacy

Self-efficacy can be increased in several ways, including; by providing clear instructions, providing the opportunity for skill development or training, and modelling the desired behavior (MOH New Zealand, 2011). There are four components to increase Self-efficacy level; mastery experience, modelling, improving physical and emotional states and verbal persuasion (Bandura, 1997) (Table 2.3).

Table 2.3 Methods for increasing self-efficacy
source Bandura, (1997)

Mastery experience	Enabling the person to succeed in attainable but increasing challenging performances of desired behaviours. The experience of performance mastery is the strongest influence on self-efficacy belief.
Modelling	Showing the person that others like themselves can do it. This should include detailed demonstrations of the small steps taken in the attainment of a complex objective (social modelling) or by giving them a brochure as a symbolic modelling
Improving physical and emotional states	Making sure person are well-rested and relaxed before attempting a new behaviour. This can include efforts to reduce stress and depression while building positive emotions- as “fear” is re-labelled as “excitement.”
Verbal persuasion	Telling the person that he or she can do it. Strong encouragement can boost confidence enough to induce the first efforts toward behaviour change.

2.5 Previous studies related to self-efficacy and self-care behaviour in diabetes management

The self-care behaviour for diabetes provide useful context in which to examine the role of self-efficacy beliefs in adherence to prevention behaviours (Maddux, 1995; Sarkar, Fisher, & Schillinger, 2006; Rodriguez, 2013). This concept was introduced from Social Cognitive Theory (SCT) in health promotion and patient education program (Bandura, 1977; Bandura, 1989; Bandura, 2004) and has been increasingly applied, both as a model of health behaviour and as a framework for developing effective health intervention in various population.

Self-efficacy is a major predictor of successful self-care behaviour in diabetes management because it will influences how people think, motivate themselves and act in order to modifying health behaviour in the desired direction (Bandura, 1997; Lenz & Shortridge-Baggett, 2002). The Self-efficacy model is suited to create educational programs to improve self-care behaviour of patients with diabetes (van der Bijl et al, 2001; McDowell et al, 2005). Wu et al. (2007) stated by enhancing self-efficacy towards self-managing the disease can be an effective way to improve disease control and to understand adherence to self-care behaviour.

Previous research findings have focused on knowledge of patients with diabetes to control the disease, but Hurley and Shea (1992) stated having knowledge does not necessarily change patients' behaviour. The evolution of research was then extended to view the other aspects that can change the behaviour. The self-efficacy concept related to patients who feel more confident if they are able to succeed at the task of self-care behaviour (Wolf, 2006). Thus, self-efficacy and self-care behaviour was identified as a key psychosocial variable related to diabetes self-management. Greater self-efficacy was a significant predictor of better self-care behavior (Johnston-Brooks et al., 2002). Furthermore, by enhancing self-efficacy in elderly with diabetes may increase the self-care behaviour by behavioural change strategies (Shi, Ostwald & Wang, 2010). Literature reviews showed by integrating self-efficacy theory and self-care behaviour in patients with diabetes appears to enhance patients' positive outcome.

In the past, Hurley and Shea (1992) conducted study on self-efficacy: strategy for enhancing diabetes self-care in the United States. This study found that patients with higher levels of self-efficacy were better able to manage their diabetes self-care behaviour. However, there is need for further research to be conducted in different places due to the homogeneity of the subjects. This cross sectional study focused on individuals who used insulin and the small sample of respondents involved between the high socioeconomic status only and lack of multi-ethnicity. This finding might also be invalid now as it was done a long time ago.

Wang and Shiu (2004) conducted a study on diabetes self-efficacy and self-care behaviour of Chinese patients living in Shanghai. The results shows patients with higher levels of self-efficacy reported that they were better able to manage their diabetes self-care. Limitation found in the study was when the researchers employed a small sample size in one clinic only and the result was difficult to generalize to other parts of the population. However, this finding lends support to the importance of incorporating the concept of self-efficacy in the design and implementation of diabetes nursing interventions.

Subsequently, Strurt et al., (2006) conducted study on complex intervention development for diabetes self-management in United Kingdom. The aim of this randomized controlled trial is to present the development and evaluation of the Self-Efficacy Goal Achievement (SEGA) nursing intervention for diabetes. It is a robust study showed an increase in patient self-efficacy and life satisfaction. However, the diabetes self-management is general rather than focusing specifically on foot care aspects.

Furthermore, Wu et al. (2007) conducted a cross sectional study on self-efficacy, outcome expectations and self-care behaviour in people with diabetes in Taiwan. The findings supported the use of the self-efficacy model as a framework for understanding adherence to self-care behaviour. The result was difficult to generalize because it employed a small sample from one clinic and not focusing on foot self-care behaviour itself.

A cross sectional study on foot-care self-efficacy and foot-care behaviour in people with peripheral neuropathy in Australia (Perrin et al., 2009). There is an association between foot-care self-efficacy and foot-care behaviour. However, the sample size was small (95 respondents) as results cannot be generalized. The foot

care behaviour scale used in this study has some decent content validity, it has not been comprehensively validated, and the scoring system is overly complicated.

Another study on foot care education and self-management behaviors in diverse veterans with diabetes by (Olson et al., 2009). This research builds on self-efficacy and self-management theory and the finding suggests the need for culturally-specific self-management education to address unique cultural preferences and barriers to care.

Shi et al., (2010) examined the effect of a hospital-based clinic intervention on glycaemic control self-efficacy and glycaemic control behaviour of patients with diabetes in China. The findings revealed that the experimental group showed statistically significant improvement in glycaemic control self-efficacy and glycaemic control behaviour immediately and four months after the intervention. However, the study does not mentioned about improvement specifically in foot self-care behaviour scores.

A descriptive study was done on relationship between foot-care self-efficacy beliefs and self-care behaviours in diabetic patients in Iran (Hamedan et al., 2012). Result shows foot self-efficacy is a predictor of good self-care behaviours. It was suggested that older diabetic patients should be advised to perform self-care behaviour and assessed for self-efficacy because they have many problems in self-care.

There is a local study on self-efficacy and self-care behaviour of Malaysian patients with type 2 diabetes (Sharoni & Wu, 2012). The finding suggested that self-efficacy can be used as a model to understand self-care behaviour. Limitation in this cross sectional survey appeared when this study is conducted only in one hospital and the convenience sampling may not represent the whole Malaysian with diabetes.

Chin, Huang, & Hsu, (2013) conducted a study on impact of action cues, self-efficacy and perceived barriers on daily foot exam practice in type 2 diabetes mellitus patients with peripheral neuropathy in Taiwan. This study suggests the health professionals to design interventions that aim to promote daily foot-exam practice. The cross sectional study design may inhibits the information about cause and effect relationship.

Fan, Sidani, Cooper-Brathwaite, & Metcalfe, (2013) conducted a study on improving foot self-care knowledge, self-efficacy, and behaviors in patients with type 2 diabetes at low risk for foot ulceration. The findings in this pilot study indicated that the foot self-care educational intervention was effective in improving foot self-care knowledge, self-efficacy and behaviors in adult patients with type 2 diabetes at low risk for foot ulceration. However, the study is not a randomized controlled trials design, not focus among elderly population and the conveniences sampling may leads to bias.

All of the previous researchers have established similar findings from their studies and proved the importance of self-efficacy and self-care behaviour in patients with diabetes in various areas. For patients with diabetes to carry out a high level of self-care behaviour, they must acquire a high self-efficacy in their ability for disease management (Wangberg, 2007). However, there are some limitations from the previous studies such as; study design does not explain on cause and effect

relationship, inadequate sample size, nonprobability sampling and study was conducted long time ago. Thus, this study proposed to contribute more information to existing theory in our local setting focusing on self-efficacy and foot self-care behaviour among elderly population in institutionalized care. By integrating the Self-efficacy model in foot self-care behaviour program may increase the confidence level and self-compliance in caring the feet among elderly with diabetes.

2.6 A new conceptual framework; the self-efficacy enhancing program on foot self-care behaviour of elderly with diabetes

The rational for developing the self-efficacy enhancing program is to improve self-care behaviour of elderly with diabetes in taking care of their feet. As the person become older, they need to stay healthy and being independently. This behaviour can improve the health outcome and reduce the complication rate related to diabetes foot problems. The self-efficacy enhancing program is to empower the elderly with diabetes to perform foot self-care behaviour effectively.

Previous literatures show there was a significant relationship between self-efficacy and self-care behaviour of patients with diabetes. Self-efficacy has been demonstrated as a main predictor in promoting self-care behaviour by behaviour change. In this study, the development of self-efficacy enhancing program on foot self-care behaviour of elderly with diabetes are designed via new conceptual framework based on Self-efficacy theory (Bandura, 1977). For example, a person to perform foot care (behaviour) for health reasons (prevent foot complication (outcome), he must believe both that foot care will benefit his foot health (foot care outcome expectation) and also that he is capable of practicing foot care (foot care efficacy expectation).

The main reason for the development of self-efficacy enhancing program is to improve foot self-care behaviour of elderly with diabetes and to prevent feet complications related to diabetes. In Malaysia, least researchers are engaged in theory based interventions in relation to diabetes foot self-care behaviour. Thus, by introducing new diabetes educational program may improve current traditional educational program given from health care provider.

The self-efficacy enhancing program on foot self-care behaviour of elderly with diabetes is an important part in this framework (Figure 2.3). Strategies to implement the self-efficacy enhancing program are described below are derived from the nursing process (Berman & Snyder, 2012).

Firstly, demographic factors (e.g. age, ethnicity, gender, education level, marital status, number of children and patterns of living) and clinical characteristics may influence the foot self-care behaviour. In this phase patients’ demographic data, clinical characteristics, independent level, cognitive level and depression will be gathered. The data will be collected through questionnaire form and blood tests.

Subsequently, both elderly with diabetes and health provider will make a specific judgment about current problem in relation to diabetes foot problems. This is involves actual and potential issues whether an elderly with diabetes is at risk to

develop any problem. During this phase also, elderly with diabetes will be assessed on current behaviour on foot self-care, readiness and preparedness for health improvement in their feet. This will be measured with the scaling question on foot self-care behaviour, foot care efficacy expectation, foot care outcome expectation, knowledge on foot care and quality of life.

A plan of action will be developed after both an elderly with diabetes and nurses have set the diagnosis. Self-efficacy goals and take a strategic action must come from general to specific. For example, foot self-care behaviour, inspection on feet might be a goal but it needs more detail about the steps and objectives on how they check on their own feet. Guide the elderly with diabetes to set clear, realistic and achievable targets for the expected good outcome according to time frame.

Implementation phase is where elderly with diabetes perform the goal of desired behaviour that has been established in planning phase. For example, health provider will educate, counsel and inspire the elderly with diabetes about foot self-care behaviour. The health provider will integrate the Self-efficacy theory to enhance of good foot self-care behaviour for the elderly with diabetes along the program. Activities in this session will involves on seminar presentation, diary, serial visits, telephone and/ or short message service (SMS) follow up. During this time, the elderly with diabetes will implement the foot self-care behaviour with self-confident independently. The health provider should be bear in mind that other variables such as foot care efficacy expectation, foot care outcome expectation, knowledge and quality of life are the factors may influence the successfulness of the foot self-care behaviour intervention program.

This evaluation is the last phase, where health provider and elderly with diabetes will determine the goals is achieved or not. The primary outcomes is the foot self-care behaviour will be improved, the foot self-care behaviour worse, or there is no significant difference of foot self-care behaviour before and after implementation phase. The secondary outcome are level of foot care efficacy expectation, foot care outcome expectation, knowledge on foot care and quality of life. During this phase the effectiveness of the program will be assessed through the questionnaire form of quality of life, knowledge on foot care, foot care efficacy expectation, foot care outcome expectation and foot self-care behaviour.

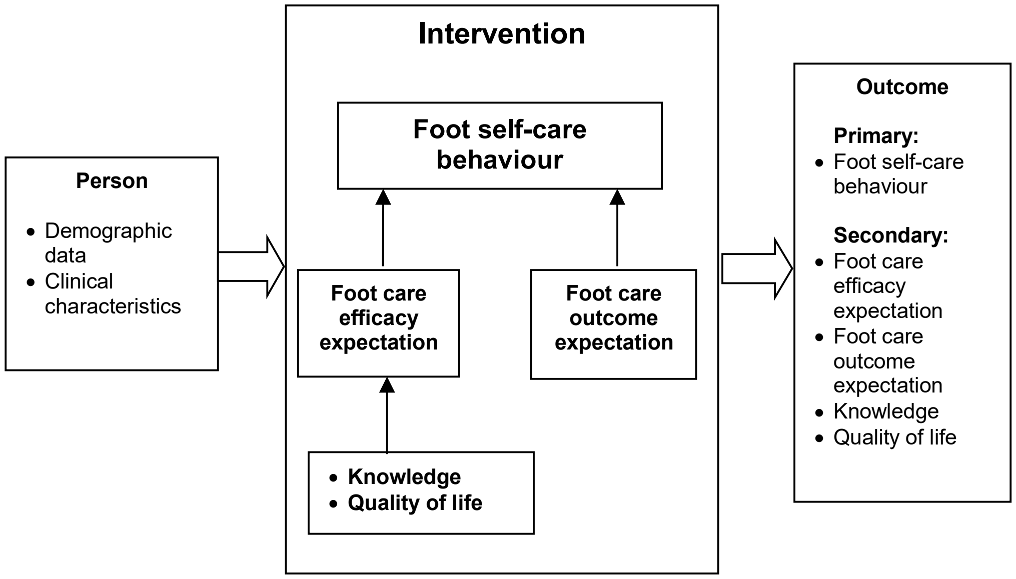

Figure 2.3 The conceptual framework for development and evaluation self-efficacy enhancing program on foot self-care behaviour of elderly with diabetes

2.7 Summary

Assisting elderly with diabetes to change their foot self-care behaviour is an important nursing role in achieving the highest possible of health outcome. There is increasing evidence that a vital component in health-related activities change is the perceived self-efficacy of the patients to behave differently. This chapter has highlighted the areas self-efficacy and foot self-care behaviour. The next chapter will discuss the methods used in this study.

Chapter 3

METHODOLOGY

3.1 Introduction

This chapter will discuss in detail on how the study will be conducted. It will include the study setting, design, population, sample, instrument used, data collection methods, data analysis, ethical consideration and pilot study.

3.2 Study setting

This study will be conducted in the institutional care for elderly named Rumah Seri Kenangan (RSK), Peninsular Malaysia. The RSK is under goverment organization, Social Welfare Department of Malaysia (JKMM), Ministry of Women, Family and Community Development. There are eleven RSKs in Malaysia, nine in Peninsular Malaysia and two in East Malaysia; RSK Bedong, Kedah, RSK Tanjong Rambutan, Perak, RSK Seremban, Negeri Sembilan, RSK Johor Bharu, Johor, RSK Kangar, Perlis, RSK Taiping, Perak, RSK Cheras, Selangor, RSK Cheng, Melaka, RSK Taman Kemumin, Kelantan, RSK Kuching and RSK Sibu. There are approximately 130 – 250 residents staying in each RSK. Figure 3.1 shows the location of RSK in Peninsular Malaysia.

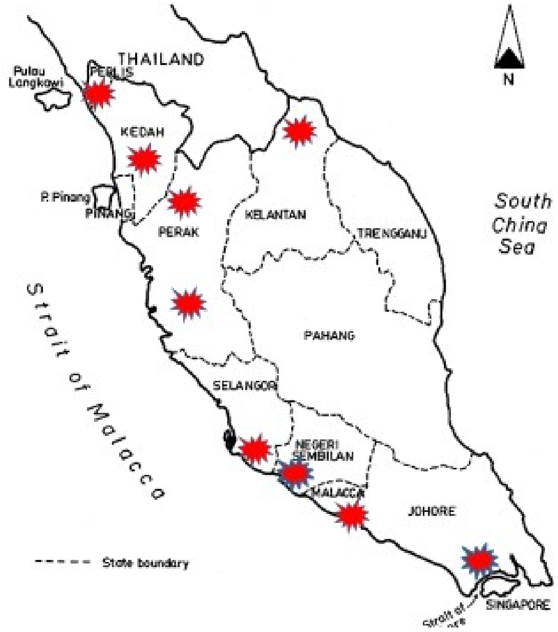

Figure 3.1 The location of RSK in Peninsular Malaysia

3.3 Study design

The design used for this research is a Randomized Control Trial (RCT) design with two groups; intervention and control, measurements will be completed before intervention and after intervention. In this single blind study, only the researcher is able to differentiate which group are under intervention or control group. The respondent will not be informed whether they are categorized under intervention or control group.

The intervention group will receive a self-efficacy enhancing program on foot self-care behaviour while the control group will receive a usual health care. The aim of the education program will that, after completed the program, the foot self-care behaviour, foot efficacy expectation, foot outcome expectation, knowledge on foot care and quality of life will be improved with respect to the program delivered. However, after the data collection process completed, the control group will be given the same program as the intervention group.

3.4 Population

3.4.1 Targeted population

There are approximately 1631 of residents staying in Peninsular RSK (Table 3.1). Currently the RSK Seremban was closed due to renovation. Majority of them are independent and able to perform their activity daily living (e.g. bathing, eating, grooming, etc.) independently. The targeted population in this study is, the respondents (elderly with diabetes) must be diagnosed with diabetes. The number of elderly diagnosed with diabetes who are totally independent in each RSK is about 40-50 residents.

Table 3.1 Number of RSK Inmates by Institution and Sex, 2013
source: Jabatan Kebajikan Masyarakat, Malaysia

Institution	Male	Female
RSK Bedong	193	99
RSK Cheng	103	84
RSK Cheras	97	78
RSK Pengkalan Chepa	73	62
RSK Johor Bahru	176	102
RSK Kangar	72	74
RSK Seremban	0	0
RSK Taiping	94	79
RSK Tanjung Rambutan	164	81
**Total**	**972**	**659**

3.5 Sample

3.5.1 Sampling method

A probability cluster sampling with specific eligibility criteria will be used in this study. There are nine RSK in the Peninsular Malaysia. However, for the time being, RSK Negeri Sembilan was closed due to renovation process, so there are total eight of RSK in Peninsular Malaysia. RSK Cheras will be excluded for pilot testing and RSK Pengkalan Chepa located in West Peninsular Malaysia. Hence, six RSK will be allocated randomly using random sequence number generator where each group (RSK) is having equal chance to be neither in the intervention group nor control group. Here, there are three RSK will be taken in intervention group and another three RSK will be placed under control group. Figure 3.2 showed how the sample will be selected and grouped in this study.

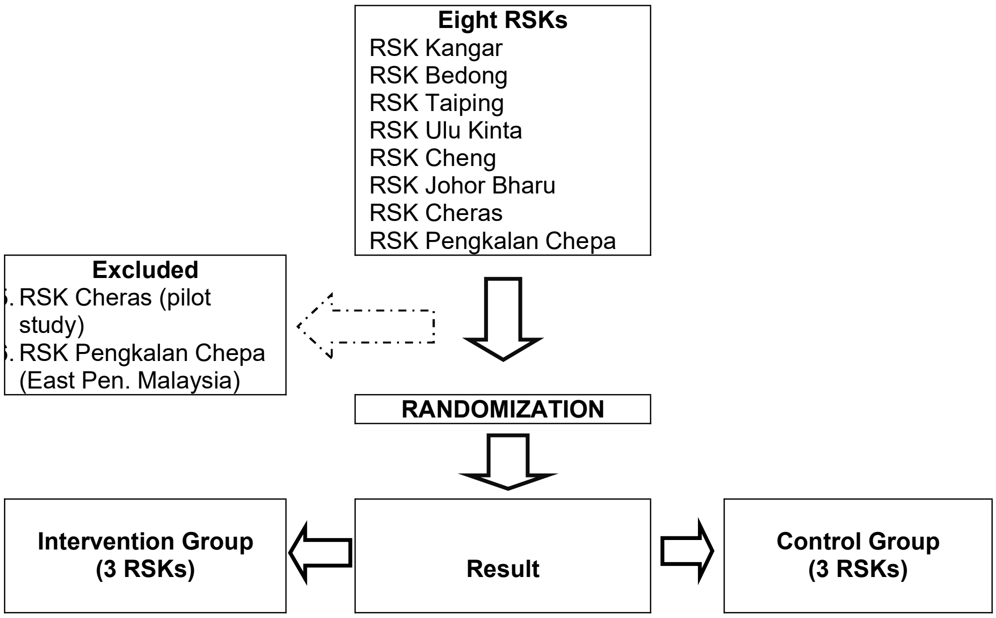

Figure 3.2 Sampling selection process

3.5.2 Sample size calculation

The sample size estimation, which is carried out using hypothesis testing two population means (Aday & Cornelius, 2006). The data is taken from a previous study conducted by Chen et al. (2011). The baseline score was; mean=32.32 with standard deviation (SD) 6.76, post intervention score has increased to 36.22 with SD 6.95. The hypothesis of no difference is to be tested at the 5% (α) 0.05 level of significance and power (1-β) = 0.80 (80%) (2-tailed). The hypothesis testing two population means sample size estimation as follow:

$H_o : \mu_1 = \mu_2$

$H_1 : \mu_1 \neq \mu_2$

$$n = \frac{2\sigma^2 [Z_{1-\alpha/2}+Z_{1-\beta}]^2}{(\mu_1 - \mu_2)^2}$$

μ_1 = estimated mean (larger)

μ_2 = estimated mean (smaller)

$$n = \frac{2(6.76)^2[1.96 + 0.842]^2}{(36.22-32.32)^2}$$

$$n = \frac{(91.40)\ (7.85)}{15.21}$$

n = 47

The above sample size formula is valid only for the simple random or systematic random sampling method. This study will employ a cluster sampling design, so it is requires a larger sample size to achieve the same precision and power (Aday & Cornelius, (2006).

Therefore, the calculated sample size using the above formula need to be multiplied by the design effect (DEFF). According Aday & Cornelius, (2006), the DEFF = 1 + (*b* – 1) *roh*, where *b* = cluster size and *roh* = rate of homogeneity (intracluster correlation). Assuming a cluster size of 6 and intracluster correlation of 0.05 (Davies et al., 2008), so the DEFF is 1.25. This means that such cluster sampling requires double the sample size of above, n = 47 x 1.25 = 59 of respondents is needed for each group.

Hence, a total of 71 are considered adequate to recruit for a dropout rate and potential of attrition (20%). Thus, total 142 eligible criteria of the respondents is needed to be approached in order to retain 118 respondents (59 respondents per group) at the end of the study.

3.5.3 Inclusion and exclusion criteria

The inclusion criteria for participation in the education program are:

1) The respondents aged 60 years or more

2) The respondents have been diagnosed with diabetes (old or new)

3) The respondents presented with or without diabetic foot problems

4) The respondents must be Malaysian.

5) The respondents who are able communicate sufficiently well (Malay) to understand the education program.

6) The respondents who are able to perform activity daily living independently (e.g., bathing, feeding, grooming etc.). The information will be gathered from the the screening process.

7) The respondents who have no major complications which would interfere with foot self-care behaviour (such as blind and stroke).

The specific exclusion criteria are:

1) Respondents who are currently has been diagnosed with mental health problem such as Schizophrenia, Bipolar Disorder and Alzheimer disease. The information will be gathered from the RSKs' health record unit.

2) Respondents who are having impaired in cognitive level. The information will be gathered from the screening process.

3) Respondents who are having depression. The information will be gathered from the screening process.

3.6 Instruments

3.6.1 Questionnaire

The questionnaire will be prepared in English and Malay language. Respondents need to answer the questionnaire independently if they were able to read and write. Interview will be conducted only for those illiterate respondents. The information that will gathered to answer objectives of the study the respondents are as follow;

Section A is demographic data. The information required are about age, ethnicity, gender, education level, marital status, number of children, frequency of being visited by children/ relatives and frineds and duration of stay in RSK.

Section B is clinical characteristics. The data will gather about FBS level, duration of diabetes, treatment of diabetes, other disease (except diabetes), smoking status, hospitalization due to diabetes problem and previous diabetes education received.

Section C measure on foot self-care behaviour. The Diabetes Foot Self-Care Behaviour Scale (DFSBS) (Chin & Huang, 2013) will be adapted in this study. The DFSBS contains 16 items. The scale had two parts: In the first part, the responses will be rated on the number of days patients performed a certain behaviour over the course of one week (0 for never, 7 for every day they performed the activity). In the second part, the responses will rated by the frequency with which patients performed a certain behaviour in general, from never (0) to always (5). The responses will be rated as a 5-point Likert scale [never/ 0 day per week (1), rarely/ 1-2 days per week (2), sometimes/ 3-4 days per week (3), often/ 5-6 days per week (4) and always/ 7 days per week (5)] (Chin & Huang, 2013). A higher score indicated good foot self-care behaviour.

Section D is measure on foot care self-efficacy (foot care efficacy expectation). The Foot Care Confidence Scale (FCCS) (Sloan, 1998) will be adapted to this study. The instrument is a public domain. There are 12 items measuring on self-confidence in managing foot care. TThe scale given into five scores; strongly not confident (score 1) to strongly confident (5). Higher scores shows high in self-confidence in managing foot care behaviour.

Section E is measure on foot care outcome expectation (FCOE). Content of the question that will be constructed must suit with education package, focus on diabetes foot complications and risk factors and the foot care for elderly with diabetes. The questionnaire will be developed based from previous literatures (Resnick, Zimmerman, Orwig, Furstenberg, & Magaziner, 2000; Lenz & Shortridge-Baggett, 2002; Chlebowy & Garvin, 2006; Wu et al., 2008; Kakudate et al., 2011; Kedem, Evans, & Chapman-Novakofski, 2014). There have six items and the scale consists of five scoring; strongly disagree (1) to strongly agree (5). Higher scores shows high in self-confidence that a foot behaviour will have the desired effect.

Section F is measure on knowledge on foot care (KFC). Content of the questions that will be constructed must suit with education package, focus on diabetes foot complications, risk factors and the foot care for elderly with diabetes in Malaysian setting. The questionnaire will be developed based from previous literatures (Pollock et al., 2004; Desalu et al., 2011; Eigenmann et al., 2011; Morey-Vargas & Smith, 2015;

American Diabetes Association, International Diabetes Federation, Clinical Practice Guideline, National Diabetes Institute, Malaysian Diabetes Association). In this section, the elderly needs to answer 11 questions with three answer choices (true, false, don't know). Each correct answer will be given 1 point. The cut-off point of this questionnaire are good (score ≥ 70%), satisfactory (score 50-69%) and poor (score <50) (Desalu et al., 2011). On the other hand, the score ranged from 0 to 11. A higher score indicated a good level of knowledge of foot care.

Section G is measure on quality of life. Neuropathy and Foot Ulcer Specific Quality of Life (FS-QOL) (Vileikyte et al., 2003) will be adapted in this study. These 27 items asked about the effect of foot issues may have on daily life and well-being. Patient need to recall (in the past 4 weeks) about their feeling, experienced the foot issue symptoms how foot issues affect their daily activities, relationships and feelings. This is Likert scale measures in several categories; (all the time, sometime, never), (very much, somewhat, not at all) and (agree, neither agree or disagree, disagree). The instrument measured should therefore include items 1-13 (symptoms) and 14-27 (psychosocial functioning). Thirteen items assess specific somatic experiences in three domains: i.e., Pain (items 1-7), Lost/reduced feeling (items 8-10); and Diffuse sensory-motor symptoms (items 11-13). Specific functional, social and emotional experiences are assessed in three domains with an additional 14 items: Restrictions in activities of daily living (items 14 – 16), and Disruptions in social relationships (items 17-20), and Emotional distress (items 21- 27). The frequency of these experiences, somatic, social and effective, are reported on 3 point scales (never, sometime, all the time). A participant's score for a domain is the mean of the items in that scale with higher scores representing more severe symptoms or greater disruption in functioning.

For each of these 27, specific items, patients are asked to judge the degree to which the somatic experience, restriction of activities, social function and emotional states have been a bother and/or important to them (Vileikyte et al., 2003). The bother /importance items were scored as 1=none; 2= some; 3= very. Weighted scores were calculated by multiplying the scale score by the corresponding bother/importance score (Vileikyte et al., 2003). Multiplying the frequency of experience by its bother and importance provides a more detailed picture of the degree to which the specific experience impacts satisfaction or quality of life (Vileikyte et al., 2003).

3.6.2 Quality control

3.6.2.1 Validity and Reliability of the instruments

Measurement of the validity and reliability of the instruments and the intervention program are major concern in any research. The measurements of these instrument and intervention program represented the concept or aim of research questions; otherwise any conclusions drawn from this study was invalid.

Three instruments used in this study was adapted from previous study. The Neuropathy and Foot Ulcer Specific Quality of Life (FS-QOL) (Vileikyte et al., 2003), The Foot Care Confidence Scale (FCCS) (Sloan, 1998) and The Diabetes Foot Self-

Care Behaviour Scale (DFSBS) (Chin & Huang, 2013) has been tested for the instrument validity previously. Table 3.2 shows the validity and reliability of the instruments adapted in this study.

Table 3.2 The description about adapted instrument used in this study

Instrument	Validity and reliability	Approval
The Neuropathy and Foot Ulcer Specific Quality of Life (FS-QOL) (Vileikyte et al., 2003)	The instrument revealed three physical symptom measures and two psychosocial functioning measures with good reliability (alpha 0.86–0.95)	Permission has been granted from the original researcher via email prior to this study.
The Foot Care Confidence Scale (FCCS) (Sloan, 1998)	The FCCS had a Cronbach's alpha of 0.92 and the instrument is a practical to be used in many settings (Sloan, 1998).	The instrument is a public domain
The Diabetes Foot Self-Care Behaviour Scale (DFSBS) (Chin & Huang, 2013)	Internal consistency was acceptable (Cronbach's alpha = 0.73), and interclass correlation coefficient for test retest reliability over a 2-week period was 0.92	Permission has been granted from the original researcher via email prior to this study.

In this study, the researcher will prepare the questionnaire in two languages; English and Malay. The English questionnaire will be translated into the Malay version. The English version of the questionnaire will be sent to Institute Terjemahan dan Buku Malaysia (ITBM) for translation process. Basically, the first stage involved the forward translation by bilingual translator independently translating the instrument from English into Malay. After that, another translator who had not seen the original questionnaire translated the new translation back into the English language. This technique called semantic translation and the translators are from person who has medical and health sciences background.

Validity and reliability for the development intervention program and instrument of knowledge on foot care scale and foot care outcome expectation scale will be tested and piloted prior to main study. The validity of the intervention program and instruments will be tested using face and content analysis. The materials will be given to a panel of experts; consisting of supervisors, endocrinologist, diabetic nurse educator, foot care nurse and elderly with diabetes to ensure the questions are appropriate and relevant in our current practice, setting, culture and population.

Content of the intervention program and the instruments will be judge by expert panels. The purpose of it is to verify that all the items and concepts measured are representative, well defined and reviewed with an appropriate weighing to the elderly population. Content Validity Ratio (CVR) = (2ng / N) – 1, will be used for validity conformity. Where ng is the number of Subject Matter Experts (SMEs) who think the item is good and N is the total number of SMEs. In this approach, a panel of SMEs is asked to indicate whether or not a measurement item in a set of items is "essential" to the operationalization of a theoretical construct (Johnston & Wilkinson, 2009). "Essential" items or assessment tasks are ones that best represent the goal and are desired. The value lies between -1.00 to +1.00, where a CVR = 0.00 means that 50%

of the SMEs in the panel size of N believe that the portfolio task is essential thereby valid (Johnston & Wilkinson, 2009).

The reliability will be tested with internal consistency. Reliability of result was tested using Cronbach's alpha test after the pilot study is conducted. The reliability coefficient (alpha) can range from 0 to 1, with 0 representing an instrument with full of error and 1 representing total absence of error. A reliability coefficient (alpha) of 0.70 or higher is considered acceptable reliability (Aday & Cornelius, 2006).

Factor analysis will be performed to examine interrelationships among the large number of variables and disentangles those relationships to identify clusters of variables that are most closely linked together (factors) (Grove, Burns & Gray, 2012). The responses to the instrument items will be subjected to principal component analysis to identify underlying dimensions or factors. A threshold of 0.60 will be used to determine which statements are considered to be part of a factor. Internal consistency for each factor, as measured by Cronbach's alpha is acceptable as the alpha values for all the factors are above 0.80.

3.6.2.2 Pilot Study

Pilot study is a miniature of the proposed study. Pilot study will be conducted to identify and investigate the feasibility of the suggested study and to detect any possible errors in the data collection instruments such as any ambiguous words and instructions, inadequate time and to confirm whether the variables defined by the operational definitions were actually measurable and observable (Brink, 2006; Polit & Beck, 2006).

This pilot study will be conducted on XXX to XXX involves 10% (XXX) of the population targeted. Respondents involved in pilot study will be excluded from the real study. This will be done to check the understanding of questionnaire given to the respondents. A part from that, the pilot study is to identify the feasibility of data collection procedures, completed time and the administration of the questionnaire for clarity and respondent's willingness to complete it. The results from this pilot study will provide some indication of anticipated problem that may arise in the research study.

Approximately at about 30 minutes is needed for respondents to complete the questionnaire. Any changes to the items of instrument will be testing for reliability using Cronbach's alpha test to detect the internal consistency or reliability acceptable results.

All the participating patients will be interviewed at the end of the pilot program. They will be asked for their opinion on the practicability and expected effectiveness of the education program. Practicability is defined here as the degree to which the various parts of the program can be carried out and fitted into the present way of working. The expected effectiveness will be described as the extent to which the participants expected the program to contribute to improving foot self-care behaviour for diabetes management.

3.7 Data collection

The research project comprised three phases. In the first step, the education program and the instruments (knowledge on foot care and outcome expectation) will be developed by making use of the principles of Bandura's Social Cognitive Theory (SCT) with the emphasis on taking action to promote self-efficacy (van de Laar & van der Bijl 2001). The program materials; a questionnaire and the education program (to be given to the respondents) a diary/ folder (for the health staff in the RSK) and teaching folder and written instructions (for researcher) will be developed in this phase.

In the second step, the education program will be assessed in a pilot study, followed by implementation of main study. Lastly, evaluation process of the program will be assessed.

3.7.1 Step 1: Development of the materials for the intervention program

The several steps are undertaken for the development of the education program. The information will be gathered from local professional body/organization such as Malaysian Diabetes Association, National Diabetes Registry and National Diabetes Institute. Interviews with diabetic nurse educators, foot care nurse, and endocrinologist from hospitals/ clinics/ organisations to get information about foot diabetes education. Elderly with diabetes also will be interviewed to on the most common problem situations they experience in following diabetes foot self-care behaviour recommendations. Development the material of the program will be established such as follows:

- The questionnaire measuring on knowledge of foot care and foot care outcome expectation.
- The intervention module; teaching materials, pamphlet and instructions for the researcher.
- A checklist for respondent and health provider in the RSK. The checklist/ reminder will be used to monitor the foot self-care behaviour of the respondents.

The theoretical background of the education program is based from Albert Bandura's Self-efficacy theory. There a few components for enhancing the self-efficacy level such as mastery experience, modelling, supporting psychological states and verbal persuasion (Bandura, 1997). In this study, self-efficacy enhancing activities will be applied together with knowledge transfer and the researcher's approach. Knowledge transfer will be conducted during seminar, demonstration and follow up session to increase their knowledge and awareness. Self-efficacy enhancing activities will be stressed during and after the education session. A good researcher-respondents' relationship need to be established so that the respondents will trust and follow to the researcher's instruction. Respondents will be encouraged to establish a realistic goals on new targets (tailored action plan) in performing foot self-care behaviour. They need to start work in a small realistic steps so that they will be more focus, confidence and become motivated to perform the desired behaviour (mastery experience). Respondents will be given a daily reminder/ checklist and a hand-out

(symbolic modelling) so that they will perform the foot self-care behaviour daily. In order to maintain their confidence level, a positive feedback and strong encouragement need to be given continuously (verbal persuasion). During follow up, the researcher will discuss with respondents, sharing experience each other (social modelling) and get feedback on goals, examination of obstacles and problem situations (self-appraisal). The respondents will be advised to be more responsible in performing of foot self-care behaviour regularly and participate actively to the recommended lifestyle adjustments. Table 3.3 showed the integration of self-efficacy enhancing activities in the intervention phase.

Table 3.3 The integration of self-efficacy enhancing activities in the intervention phase

Tasks	Self-efficacy enhancing activities
Knowledge transfer	• Oral information by care the researcher<br>• Written/ oral information in a slide and pamphlet
Self-efficacy enhancement activities	• Establishing goals together with the respondent<br>• Agreement on new targets<br>• Working with small realistic steps<br>• Building up a relationship of trust with the respondent with attention being paid to difficult situations and obstacles<br>• The respondent starts to try the behaviour independently<br>• Positive feedback and encouragement<br>• The respondent keeps a daily reminder/ checklist<br>• Sharing experience each other<br>• Referring to hand-out for skills improvement<br>• Feedback on goals during consultation, examination of obstacles and problem situations
The researcher's approach	• Leave responsibility to the patient<br>• Encourage an active role for the patient<br>• Recommendations on lifestyle adjustments for the patient

Subsequently, submission of the draft (questionnaire, education program, teaching materials and instructions and folder) for assessment to 6 content experts (people with practical experience (clinical areas) and theoretical knowledge (academician) in the field related to this study will be conducted. Besides, a pilot study (10% from total sample required) will be conducted to assess the validity and reliability as well as practicability and feasibility of the program.

After feedback from the expert persons and preliminary data obtained, the development of second draft of the materials (questionnaire, education program a folder, teaching folder and instructions) on the basis of a careful weighing of the comments of the content experts will be conducted.

3.7.2 Step 2: The intervention; the education program

All the independent elderly who stayed in the RSK will be invited to participate in this study. The program will be carried for 12 weeks and consisted of a total of 4 visits: 1) to get preliminary data/ assessment, 2) the education program 3) Follow up and 4) evaluation. Figure 3.3 illustrated the flow chart of recruitment and the intervention program. Refer to Table 3.4 for the whole activities of the intervention program.

1) Screening, assessment, consent approval and baseline data

If the respondents agree to take part in this study they must take part in screening session. All of them will be screen for glucose level (Fasting Blood Sugar (FBS).

Also, the respondents will be assessed for level of independence in activities of daily living. Respondents will be assessed with The Katz Index of Independence in Activities of Daily Living (ADL) (Katz et al., 1970; Shelkey, Mason, & Wallace, 2012). The instrument used to assess functional status as a measurement of the client's ability to perform activities of daily living independently (Meiner & Lueckenotte, 2006; Shelkey, Mason, & Wallace, 2012). A score of 6 indicates full function, 4 indicates moderate impairment, and 2 or less indicates severe functional impairment (Shelkey, Mason, & Wallace, 2012).

Subsequently, the respondents will be assessed for cognitive level and depression level. The reason of screening is therefore, to ensure that they are absent from cognitive impairment and depression. All respondent will be assessed for cognitive level with the Malay version of Elderly Cognitive Assessment Questionnaire (ECAQ) (MOH, 2007). The Malay ECAQ refers to 10 items divided into three parts which are memory (3 questions), orientation (six questions) and recall (1 question) (MOH, 2007). Maximum score is 10; ≥7 is normal, 5-6 borderline and ≤4 dementia (MOH, 2007). The Malay Geriatric Depression Scale (GDS) four items will be used to examine the depression level (MOH, 2007). At the cut-off point of ≥2 in detecting depression (MOH, 2007).

The respondents also will be excluded if they have been diagnosed with mental illness. The information about the disease will be gathered from health staff in the RSK. Otherwise, without this screening the performance of elderly (with mental health, dependence in ADL, cognitive impairment and depression) in foot self-care behaviour may influence the effectiveness of the program. These strategies are to minimise bias that may influence the effectiveness of the program and result findings.

The respondents are also will be informed on the study purpose verbally with the subject information sheet and the respondents also will be asked to give (signature or thumb print) an informed consent.

Baseline data (a set of questionnaire; demographic data, clinical characteristics, foot self-care behaviour (DFSBS), foot care efficacy expectation (FCCS), foot care outcome expectation (FCOE) knowledge on foot care (KFC) and quality of life (FS-QOL), need to be filled in by the respondents or by the researcher (if respondent are having difficulty to read and write).

2) The education program

Referring to Table 3.4, during first meeting, the intervention group will be given with the self-efficacy enhancing program on foot self-care behaviour. The intervention is in a group seminar of education regarding knowledge on foot care to the respondents. The programs encompasses of several activities; 20 minutes of seminar presentation. The intervention or education content covered the following topics: awareness of risk factors and its complications, foot self-care behaviour including daily washing, inspecting foot for problems, moisturizing, wearing proper shoes and socks; toenail and feet care; and when to seek help from a healthcare professional. The demonstration is all about techniques of inspection and washes the feet as well as nail care. At the end of session, the researcher will encourage active role and leave the respondents to perform foot self-care behaviour independently. The intervention group will be participated with the series of weekly follow-up via appointments/ visits and/ or telephone call for up to 12 weeks. Meanwhile the control group are only will receive a usual health care.

In order to ensure compliance status of the respondents in intervention group, the respondents and the health care provider will be given a pamphlet regarding foot self-care behaviour for them to get more information on the topic itself. They are also will receive a folder/ chart to measure on the respondents' foot self-care behaviour daily. The health provider will be advised to remind the respondents about the foot self-care behaviour every day. A telephone call to the health provider will be done weekly till the end of intervention program. This is to measure the progress of self-care behaviour of the respondents.

3) Follow up

A follow up session will be conducted at second meeting (four weeks after first meeting). In the intervention group, the respondents will be gathered in a group for a sharing and question and answer (Q & A) session. For respondents who manage to perform foot self-care behaviour effectively will be asked to be a mentor and share their skills and experiences with others. The objective of this session is to sustain the self-efficacy level of elderly with diabetes to continue performs foot self-care behaviour regularly. A specific guidance and motivation will be given individually if there is any respondent who do not able to perform foot self-care behaviour effectively. Whereas, the control group will only receive a regular visit from the researcher. Both intervention and control group will be assessed (second measurement) with the questionnaire (DFSBS, FCCS, FCOE, KFC, FS-QOL).

4) Evaluation (post-test)

After two sessions (8 weeks) of the program, both the intervention and control group will be asked again to answer the questionnaire (KFC, FCCS, FCOE, DFSBS, FS-QOL) for evaluation of the effectiveness on the program. The foot self-care behaviour is a set of self-care activities that elderly with diabetes undertake to fulfil personal goals with regard to their disease. A time period of evaluation is referring to previous study by A time period of follow up and booster session is referring to previous studies by (Baba et al., 2014; Fan et al., 2013). Figure 3.3 illustrates the process of recruitment, intervention and the evaluation program. Table 3.4 describes the whole activities of the intervention program.

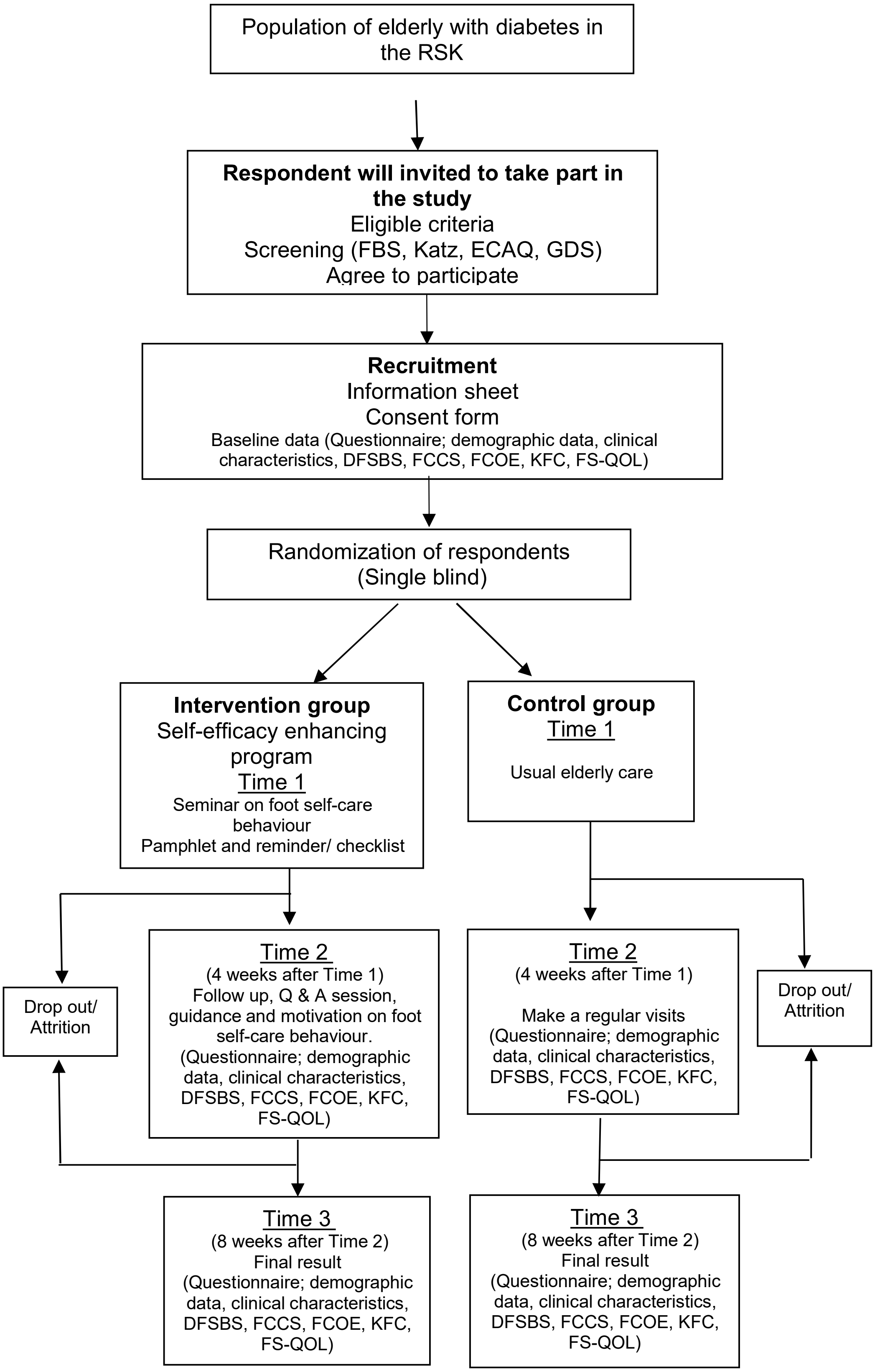

Figure 3.3 Flow chart of recruitment and the intervention program

Table 3.4 The whole activities of the intervention program

Time	Activities		Mode of program	Duration
Preliminary data/ assessment	*Screening*<br>Blood sugar level testing (FBS)<br>Independence level (Katz Index)<br>Cognitive level (ECAQ)<br>Depression level (GDS)		Taking blood<br>Questionnaire	20 minutes
	Consent form<br>The respondents need to fill in the questionnaire; demographic data, clinical characteristics, DFSBS, FCCS, FCOE, KFC, FS-QOL<br>Respondent will be randomly allocated		Consent form<br>Questionnaire	20 minutes
Time	Activities		Mode of program	Duration
	Intervention group	Control group		
First meeting<br>Time 1<br>(week 1)	The education program<br>For the elderly:<br>A group seminar regarding knowledge on foot care:<br>Awareness of risk factors and its complications, foot self-care behaviour: daily washing, inspecting foot for problems, moisturizing, wearing proper shoes and socks, toenail and feet care, when to seek help from a healthcare professional.<br>Note:<br>The health provider and the respondents will receive a pamphlet as a guidance and a checklist as a reminder in performing foot self-care behaviour daily	Usual health care	Power point presentation	20 minutes
Week<br>1, 2, 3	Follow up to the health provider	None	Phone call	5 minutes
Week 4	Visit, question and answer session, continuously give guidance and motivation on foot self-care behaviour. The respondents need to fill in the questionnaire; DFSBS, FCCS, FCOE, KFC, FS-QOL	Regular visits. The respondents need to fill in the questionnaire; DFSBS, FCCS, FCOE, KFC, FS-QOL	Discussion<br>Questionnaire	20 minutes
Week 5, 6, 7, 8, 9, 10, 11	Follow up to the health provider	None	Phone call	5 minutes
Week 12	Evaluation<br>Last visit. Keep reminding to maintain the foot self-care behaviour among the elderly<br>The respondents need to fill in the questionnaire; DFSBS, FCCS, FCOE, KFC, FS-QOL	Evaluation<br>Last visit. Keep reminding to maintain the health care among the elderly<br>The respondents need to fill in the questionnaire; DFSBS, FCCS, FCOE, KFC, FS-QOL	Discussion<br>Questionnaire	20 minutes

3.8 Ethical considerations

Ethical approval of conducted research is very important as it deals with human and one's privacy. The necessary ethical approval will be obtained before the research project from The Ethics Committee for Research Involving Human Subjects Universiti Putra Malaysia (JKEUPM) (Reference number: XXX) and Department of Social Welfare/ Jabatan Kebajikan Masyarakat Malaysia (JKMM) as well as Ministry of Women, Family and Community Development (MWFCD) ethical committee (Reference number: JKMM XXX) will be attempted. If required, Good Clinical Practice (GCP) certificate will be attached in the ethic application form.

A formal letter from the Head of Community Department, Faculty of Medical and Health Sciences, UPM to the director of the JKMM seeking permission will be given prior of data collection (Reference number: attachment XXX). Subsequently, copy of all letter will be courtesy copy (cc) to the director of each RSK in peninsular Malaysia so as all the staff will be aware about the study.

The respondents will be explained about the topic of study. In order to assist the respondents in understanding the reason for the study, both information sheet and consent form will be written in two languages; Malay and English. An information sheet describing the study in the language of respondents' choice will be given to them to read or will be read to them by the researcher prior to written consent taking. All respondents who want to involve in this study must be voluntary and they are free to turn down to be included in this study without comment or penalty.

Full confidentiality and anonymity of the data will be maintained. All the information will be kept in a private envelope and soft data will be entered into a secured computerized database.

3.9 Data analysis/management

The data collected will be analysed using the descriptive and inferential statistics. Statistical Packages for the Social Sciences version 22 for Windows will be used. Data screening will be performed to check the accuracy of data entry and to make sure that there is no missing value. Statistical test used are descriptive and inferential test. The level of significance will be set at $p < 0.05$ (two-tailed) for all inferential analysis. The statistical report and conclusion will be written using APA style format.

Firstly, factor analysis will be used as a data reduction techniques to reduce a large number of variables to smaller set underlying factors that can summarizes the essential information contained in the variable and this procedure called as factor analysis (Coakes & Steed, 2003). The responses to the items of FCCS, and FCOE will be subjected to principal component analysis to identify underlying dimensions or factors. A threshold of 0.60 will be used to determine which statements are considered to be part of a factor. Internal consistency or reliability acceptable results for each factor and items that will be tested by Cronbach's alpha is acceptable as the alpha values for all the factors are above 0.80.

The demographic data and clinical characteristics will be analysed with descriptive statistics. The mean and standard deviation (SD) for continuous variables and the count (n) and percentages (%) for the dichotomous or nominal data (categorical data) will be calculated.

Meanwhile, to answer the hypothesis testing, an inferential statistics such as chi-square, mixed design analysis of variance (ANOVA) and repeated measure analysis of variance (RM-ANOVA) (if all statistical assumption for parametric tests are meets) will be used to compare the effectiveness before and after the intervention program. Table 3.5 showed the type of analysis that will used for each research objectives.

Table 3.5 Type of analysis based on research objectives.

Objective	Type of analysis
Objective 1<br>To determine the:	
• demographic data of respondents	Descriptive (mean, SD) and (n, %)
• clinical characteristics of respondents	Descriptive (mean, SD) and (n, %)
• foot self-care behaviour of respondents	Descriptive (mean, SD)
• foot care efficacy expectation of respondents	Factor analysis, Descriptive (mean, SD)
• foot care outcome expectation of respondents	Factor analysis, Descriptive (mean, SD)
• knowledge on diabetes foot care of respondents	Descriptive (mean, SD) and (n, %)
• quality of life of respondents	Descriptive (mean, SD)
Objective 2<br>To determine the effects of self-efficacy enhancing program on quality of life, knowledge on diabetes foot care, foot care efficacy expectation, foot care outcome expectation and foot self-care behaviour of elderly with diabetes between group (intervention and control) at Time 1 (before the intervention education program), Time 2 (four weeks after the intervention education program) and Time 3 (12 weeks after the intervention education program)	Mixed between-within subjects ANOVA to examine the differences between two independent groups (intervention and control) while subjecting participants to repeated measures between T1, T2 and T3.
Objective 3<br>To determine the effects of self-efficacy enhancing program on quality of life, knowledge on diabetes foot care, foot care efficacy expectation, foot care outcome expectation and foot self-care behaviour of elderly with diabetes within group (intervention and control) at Time 1 (before the intervention education program), Time 2 (four weeks after the intervention education program) and Time 3 (12 weeks after the intervention education program)	RM-ANOVA to examine the differences in the outcomes between T1, T2 and T3. F tests with paired t tests to identify when changes in outcomes will take place.

3.10 Expected outcome

This study is to be expected at the end of study, there will be an improvement in the level of foot self-care behaviour. The secondary outcome would be an improvement in efficacy expectation, outcome expectation, knowledge on foot care and quality of life of elderly with diabetes in Rumah Seri Kenangan. The information from the findings of this study may contribute as baseline information for the ministry concern. It is hope that in future, the programs among elderly especially in institutional care can helps to increase their health status towards healthy aging.

3.11 Summary

In summary, this chapter has provided in detail of the research design, setting, population, sampling, sample size, construction of the intervention program and the questionnaire as a research tool, validity and reliability, data collection process, ethical approval, statistical analysis, pilot study and expected outcome. The next chapter will provide the data analysis of study.

References

Abdullah, M. A. H., & Abdullah, A. T. (2010). National Orthopaedic Registry Malaysia (NORM). *National Orthopaedic Registry Malaysia (NORM) 2009*. Retrieved from http://www.acrm.org.my/norm

Ahmad, B., Ramadas, A., Kia Fatt, Q., & Md Zain, A. Z. (2014). A pilot study: the development of a culturally tailored Malaysian Diabetes Education Module (MY-DEMO) based on the Health Belief Model. *BMC Endocrine Disorders*, *14*(1), 31. doi:10.1186/1472-6823-14-31

Aikens, J. E., Rosland, A. M., & Piette, J. D. (2015). Improvements in illness self-management and psychological distress associated with telemonitoring support for adults with diabetes. *Primary Care Diabetes*, *9*(2), 127–134. doi:10.1016/j.pcd.2014.06.003

Amal, N. M., Paramesarvathy, R., Tee, G. H., Ph, M., Gurpreet, K., Epid, M. P. H., & Karuthan, C. (2011). Prevalence of Chronic Illness and Health Seeking Behaviour in Malaysian Population : Results from the Third National Health Morbidity Survey (NHMS III) 2006. *Medical Journal of Malaysia*, *66*(1), 36–41.

Ambigga, K. S., Ramli, A. S., Suthahar, A., Tauhid, N., Clearihan, L., & Browning, C. (2011). Bridging the gap in ageing: Translating policies into practice in Malaysian Primary Care. *Asia Pacific Family Medicine*, *10*(1), 2. doi:10.1186/1447-056X-10-2

American College of Foot and Ankle Surgeons. (2006). A Supplement to : of DIABETIC DISORDERS A CLINICAL. *The Journal of Foot & Ankle Surgery*, *45*(5).

Ang, C. ., & Lim, Y. . (2013). Recurrent Admissions for Diabetic Foot Complications. *Malaysian Orthopaedic Journal*, *7*(3), 21–26.

Awc, L., Zaim, M., Helmy, H. H., & RAmdhan, I. (2014). Economic Impact of Managing Acute Diabetic Foot Infection in a Tertiary Hospital in Malaysia. *Malaysian Orthopaedic Journal*, *8*(1), 46–49.

Baba, M., Duff, J., Foley, L., Davis, W. a., & Davis, T. M. E. (2014). A comparison of two methods of foot health education: The Fremantle Diabetes Study Phase II. *Primary Care Diabetes*, 1–8. doi:10.1016/j.pcd.2014.05.004

Bandura, A. (1977). Self-efficacy: Toward a unifying theory of behavioral change. *Psychological Review*, *84*(2), 191–215.

Bandura, A. (1989). Social cognitive theory. *Annals of Child Development*, *6*, 1–85.

Bandura, A. (2004). Health promotion by social cognitive means. *Health Education & Behavior : The Official Publication of the Society for Public Health Education*, *31*(2), 143–164. doi:10.1177/1090198104263660

Borges, W. J., & Ostwald, S. K. (2008). Behaviors With Pies Sanos. *Western Journal of Nursing Research*, *30*(3), 325–341.

Boulton, A. (2005). The diabetic foot: epidemiology, risk factors and the status of care. *Diabetes Voice*, *50*(November), 5–7.

Brown, D. W., Balluz, L. S., Giles, W. H., Beckles, G. L., Moriarty, D. G., Ford, E. S., & Mokdad, A. H. (2004). Diabetes mellitus and health-related quality of life among older adults. Findings from the behavioral risk factor surveillance system (BRFSS). *Diabetes Research and Clinical Practice*, *65*(2), 105–115. doi:10.1016/j.diabres.2003.11.014

Calle-Pascual, A. L., Durán, A., Benedí, A., Calvo, M. I., Charro, A., Diaz, J. a., … Cabezas-Cerrato, J. (2002). A preventative foot care programme for people with diabetes with different stages of neuropathy. *Diabetes Research and Clinical Practice*, *57*, 111–117. doi:10.1016/S0168-8227(02)00024-4

Chen, M. Y., Huang, W. C., Peng, Y. S., Guo, J. S., Chen, C. P., Jong, M. C., & Lin, H. C. (2011). Effectiveness of a health promotion programme for farmers and fishermen with type-2 diabetes in Taiwan. *Journal of Advanced Nursing*, *67*, 2060–2067. doi:10.1111/j.1365-2648.2011.05678.x

Chin, Y.-F., & Huang, T.-T. (2013). Development and Validation of a Diabetes Foot Self-Care Behavior Scale. *The Journal of Nursing Research*, *21*(1), 19–25.

Chlebowy, D. O., & Garvin, B. J. (2006). Social support, self-efficacy, and outcome expectations: impact on self-care behaviors and glycemic control in Caucasian and African American adults with type 2 diabetes. *The Diabetes Educator*, *32*(5), 777–786. doi:10.1177/0145721706291760

Clinical Practice Guideline. (2004). Clinical Practice Guidelines 2004. *Ministry of Health Malaysia*.

Clinical Practice Guideline. (2009). *Clinical Practice Guidelines 2009, Management of type 2 Diabetes Mellitus (4th Edition)*. *Ministry of Health Malaysia*.

Cook, J. J., & Simonson, D. C. (2012). Epidemiology and Health Care Cost of Diabetic Foot Problems. *The Diabetic Foot*, 17–32. doi:10.1007/978-1-61779-791-0

Deakin, T. a., Cade, J. E., Williams, R., & Greenwood, D. C. (2006). Structured patient education: The Diabetes X-PERT Programme makes a difference. *Diabetic Medicine*, *23*, 944–954. doi:10.1111/j.1464-5491.2006.01906.x

Department of Statistics Malaysia. (2010). Caring society. *Department of Statistics Malaysia*, 87–109. Retrieved from http://www.statistics.gov.my/portal/download_Stats_Malaysia/files/MMS/2010/BI/08_Caring_Society.pdf

Desalu, O. O., Salawu, F. K., Jimoh, A. K., Adekoya, A. O., & Busari, O. A. (2011). Diabetic foot care: self-reported knowledge and practice among patients attending three tertiary hospital in Nigeria. *Ghana Medical Journal*, *45*(2), 60–65.

Dettori, N., Flook, B. N., Pessl, E., Quesenberry, K., Loh, J., Harris, C., … Harwell, T. S. (2005). Improvements in care and reduced self-management barriers among rural patients with diabetes. *Journal of Rural Health*, *21*(2), 172–177. doi:10.1111/j.1748-0361.2005.tb00078.x

Eigenmann, C., Skinner, T., & Colagiuri, R. (2011). Development and validation of a diabetes knowledge questionnaire. *Practical Diabetes International*, *28*(4), 166–170d. doi:10.1002/pdi.1586

Fan, L., Sidani, S., Cooper-Brathwaite, A., & Metcalfe, K. (2013). Improving Foot Self-Care Knowledge, Self-Efficacy, and Behaviors in Patients With type 2 Diabetes at Low Risk for Foot Ulceration: A Pilot Study. *Clinical Nursing Research*. doi:10.1177/1054773813491282

Faridah, K., & Azmi, M. . (2009). Retrospective study of predictors for foot ulceration among diabetic patients attending Kuala Langat Health centre from 1999 to 2008. *Journal of Community Health*, *15*(2), 43–59.

Fowler, M. J. (2008). Microvascular and Macrovascular Complications of Diabetes. *Clinical Diabetes*, *26*(2), 77–82.

Fujiwara, Y., Kishida, K., Terao, M., Takahara, M., Matsuhisa, M., Funahashi, T., … Shimizu, Y. (2011). Beneficial effects of foot care nursing for people with diabetes mellitus: An uncontrolled before and after intervention study. *Journal of Advanced Nursing*, *67*(June), 1952–1962. doi:10.1111/j.1365-2648.2011.05640.x

Garcia, T. J., & Brown, S. A. (2014). Diabetes management in the nursing home: a systematic review of the literature. *The Diabetes Educator*, *37*(2), 167–87. doi:10.1177/0145721710395330

Glanz, K., Rimer, B. K., & Viswanath, K. (2008). *Health Behavior and Health Education; theory, research and practice*. (C. T. Orleans, Ed.)*Health Behaviour and Health Education. Theory, Research, and Practice* (4th Editio.). Jossey-Bass, John Wiley & Sons, San Francisco. doi:10.1016/S0033-3506(49)81524-1

Hairi, N. N., Bulgiba, A., Mudla, I., & Said, M. A. (2011). Chronic diseases, depressive symptoms and functional limitation amongst older people in rural Malaysia, a middle income developing country. *Preventive Medicine*, *53*, 343–346. doi:10.1016/j.ypmed.2011.07.020

Hamedan, M. S., Hamedan, M. S., & Torki, Z. sadegh. (2012). Relationship between Foot-Care Self-Efficacy Beliefs and Self Care Behaviors in Diabetic Patients in Iran (2011). *Journal of Diabetes & Metabolism*, *03*(09). doi:10.4172/2155-6156.1000220

Hasimah, M. N., Nurhanani, M. ., & Ramli, M. (2010). Medical Complications Among Type 2 Diabetes Mellitus Patients at a General Hospital in East Coast. *The International Medical Journal*, *9*(1), 15–20.

Ibrahim, N. M., Shohaimi, S., Chong, H.-T., Rahman, A. H. A., Razali, R., Esther, E., & Basri, H. B. (2009). Validation study of the Mini-Mental State Examination in a Malay-speaking elderly population in Malaysia. *Dementia and Geriatric Cognitive Disorders*, *27*(3), 247–53. doi:10.1159/000203888

International Diabetes Federation. (2013). *IDF DIABETES ATLAS Sixth edition*. Retrieved from www.idf.org/diabetesatlas

Jeffcoate, W. J., & Harding, K. G. (2003). Diabetic foot ulcers. *The Lancet*, 1–7.

Johnston, P., & Wilkinson, K. (2009). Enhancing Validity of Critical Tasks Selected for College and University Program Portfolios. *National Forum of Teacher Educational Journal*, *19*(3), 1–6.

Kakudate, N., Morita, M., Fukuhara, S., Sugai, M., Nagayama, M., Isogai, E., … Chiba, I. (2011). Development of the outcome expectancy scale for self-care among periodontal disease patients. *Journal of Evaluation in Clinical Practice*, *17*, 1023–1029. doi:10.1111/j.1365-2753.2010.01425.x

Kargar Jahromi, M., Ramezanli, S., & Taheri, L. (2014). Effectiveness of Diabetes Self-Management Education on Quality of Life in Diabetic Elderly Females. *Global Journal of Health Science*, *7*(1), 10–15. doi:10.5539/gjhs.v7n1p10

Kedem, L. E., Evans, E. M., & Chapman-Novakofski, K. (2014). Psychometric Evaluation of Dietary Self-Efficacy and Outcome Expectation Scales in Female College Freshmen. *Behavior Modification*, *38*(6), 852–877. doi:10.1177/0145445514543467

Kiau, B. B., Kau, J., Nainu, B. M., Omar, M. A., Saleh, M., Keong, Y. W., & Hock, L. K. (2014). Prevalence, awareness, treatment and control of Hypertension among the elderly: the 2006 National Health and Morbidity Survey III in Malaysia. *Malaysian Family Physician*, *9*(3), 12–19. Retrieved from http://www.ncbi.nlm.nih.gov/pubmed/24145262

Ko, I. S., Lee, T. H., Kim, G. S., Kang, S. W., & Kim, M. J. (2011). Effects of Visiting Nurses' Individually Tailored Education for Low-Income Adult Diabetic Patients in Korea. *Public Health Nursing*, *28*(5), 429–437. doi:10.1111/j.1525-1446.2011.00941.x

Letchuman, G. R., Nazaimoon, W. M. W., Mohamad, W. B. W., Chandran, L. R., Tee, G. H., Jamaiyah, H., … Faudzi, Y. A. (2010). Prevalence of Diabetes in the Malaysian National Health Morbidity Survey III 2006. *Medical Journal of Malaysia*, *65*(3), 173–179.

Leung, P. C. (2007). Diabetic foot ulcers — a comprehensive review. *The Royal Colleges of Surgeons of Edinburgh and Ireland*, *5*(4), 219–231. doi:10.1016/S1479-666X(07)80007-2

Lincoln, N. B., Radford, K. a, Game, F. L., & Jeffcoate, W. J. (2008). Education for secondary prevention of foot ulcers in people with diabetes: a randomised controlled trial. *Diabetologia*, *51*(11), 1954–61. doi:10.1007/s00125-008-1110-0

Litwak, L., Goh, S.-Y., Hussein, Z., Malek, R., Prusty, V., & Khamseh, M. E. (2013). Prevalence of diabetes complications in people with type 2 diabetes mellitus and its association with baseline characteristics in the multinational A1chieve study. *Diabetology & Metabolic Syndrome*, *5*(1), 57. doi:10.1186/1758-5996-5-57

Mafauzy, M. (2000). The problems and Challenges of the aging population of Malaysia. *Malaysian Journal of Medical Sciences*, *7*(1), 7–9.

Mafauzy, M., Hussein, Z., & Chan, S. P. (2011). The Status of Diabetes Control in Malaysia : Results of DiabCare 2008. *Medical Journal of Malaysia*, *66*(3), 175–181.

Markle-Reid, M., Browne, G., Weir, R., Gafni, A., Roberts, J., & Henderson, S. R. (2006). The effectiveness and efficiency of home-based nursing health promotion

for older people: a review of the literature. *Medical Care Research and Review : MCRR*, *63*(5), 531–69. doi:10.1177/1077558706290941

Mazlina, M., Shamsul, A. S., & Jeffery, F. A. S. (2011). Health-related Quality of Life in Patients with Diabetic Foot Problems in Malaysia. *Medical Journal of Malaysia*, *66*(3), 234–238.

Ministry of Health Malaysia. (2010). *National Strategic Plan For Non-Communicable Disease*.

Ministry of Health Malaysia. (2011). National Health and Morbidity Survey 2011 (NHMS 2011).

Misliza, A., & Ayu, S. M. (2009). Sociaodemographic And Lifestyle Factors as The Risk of Diabetic Foot Ulcer in The University Of Malaya Medical Centre. *Journal of University Malaya Medical Centre*, *12*(1), 15–21.

MOH New Zealand. (2011). RapidE : Chronic Care A systematic review of the literature on health behaviour change for chronic care, (August).

Morey-Vargas, O. L., & Smith, S. a. (2015). BE SMART: Strategies for foot care and prevention of foot complications in patients with diabetes. *Prosthetics and Orthotics International*, *39*(1), 48–60. doi:10.1177/0309364614535622

Mustapha, F., Omar, Z., Mihat, O., Md Noh, K., Hassan, N., Abu Bakar, R., … Allotey, P. (2014). Addressing non-communicable diseases in Malaysia: an integrative process of systems and community. *BMC Public Health*, *14*(Suppl 2), 1–6. doi:10.1186/1471-2458-14-S2-S4

Nadia M Saleh, Shebl, A. M., Hatata, E. S. Z., & Refiei, M. R. (2012). Impact of Educational Program about Foot Care on Knowledge and Self Care Practice for Diabetic Older Adult Patients. *Journal of American Science*, *8*(12), 1444–1452.

Nam, S., Chesla, C., Stotts, N. a, Kroon, L., & Janson, S. L. (2011). Barriers to diabetes management: patient and provider factors. *Diabetes Research and Clinical Practice*, *93*(1), 1–9. doi:10.1016/j.diabres.2011.02.002

Nather, A. (2007). Team Approach for Diabetic Foot Problems. *Malaysian Orthopaedic Journal*, *1*(2), 3–6.

National Diabetes Registry. (2013). *National Diabetes Registry Report* (Vol. 1).

Olson, J. M., Hogan, M. T., Pogach, L. M., Rajan, M., Raugi, G. J., & Reiber, G. E. (2009). Foot care education and self management behaviors in diverse veterans with diabetes. *Patient Preference and Adherence*, *3*, 45–50. doi:10.2147/PPA.S4349

Ooi, C. C. P., Loke, S. C., Zaiton, A., & Zaitun, Y. (2011). Cross-sectional study of Older Adults with Type 2 Diabetes Mellitus in Two Rural Public Primary Healthcare Facilities in Malaysia. *Medical Journal of Malaysia*, *66*(2), 108–112.

Perrin, B. M., Swerissen, H., & Payne, C. (2009). The association between foot-care self efficacy beliefs and actual foot-care behaviour in people with peripheral neuropathy: a cross-sectional study. *Journal of Foot and Ankle Research*, *2*, 3. doi:10.1186/1757-1146-2-3

Pollock, R. D., Unwin, N. C., & Connolly, V. (2004). Knowledge and practice of foot care in people with diabetes. *Diabetes Research and Clinical Practice*, *64*(2), 117–22. doi:10.1016/j.diabres.2003.10.014

Raingruber, B. (2014). *Health Promotion Theories* (pp. 53–94). Jones & Bartlett Learning.

Rampal, L., Loong, Y., Azhar, M., & Sanjay, R. (2010). Enhancing Diabetic Care in the Community in Malaysia : Need for a Paradigm Shift. *Malaysian Journal of Medicine and Health Sciences*, *6*(January).

Resnick, B., Zimmerman, S. I., Orwig, D., Furstenberg, A.-L., & Magaziner, J. (2000). Outcome Expectations for Exercise Scale: Utility and Psychometrics. *Journal of Gerontology Social Sciences*, *55*(6), S352–S356. doi:10.1093/geronb/55.6.S352

Rodriguez, K. M. (2013). Intrinsic and extrinsic factors affecting patient engagement in diabetes self-management: perspectives of a certified diabetes educator. *Clinical Therapeutics*, *35*(2), 170–8. doi:10.1016/j.clinthera.2013.01.002

Rosenstock, I. M., Strecher, V. J., & Becker, M. H. (1988). Social Learning Theory and the Health Belief Model. *Health Education & Behavior*, *15*(2), 175–183. doi:10.1177/109019818801500203

Saleh, F., Mumu, S. J., Ara, F., Hafez, M. A., & Ali, L. (2014). Non-adherence to self-care practices & medication and health related quality of life among patients with type 2 diabetes: a cross-sectional study. *BMC Public Health*, *14*(1), 431. doi:10.1186/1471-2458-14-431

Sarkar, U., Fisher, L., & Schillinger, D. (2006). Is Self-Efficacy Associated With Diabetes Self-Management Across Race / Ethnicity. *Diabetes Care*, *29*(4), 823–829.

Shaw, J. E., Sicree, R. a, & Zimmet, P. Z. (2010). Global estimates of the prevalence of diabetes for 2010 and 2030. *Diabetes Research and Clinical Practice*, *87*(1), 4–14. doi:10.1016/j.diabres.2009.10.007

Shelkey, M., Mason, V., & Wallace, M. (2012). Katz Index of Independence in Activities of Daily Living (ADL). *Best Practices in Nursing Care to Older Adults*, (2). doi:10.1097/00004045-200105000-00020

Shrivastava, S. R., Shrivastava, P. S., & Ramasamy, J. (2013). Role of self-care in management of diabetes mellitus. *Journal of Diabetes and Metabolic Disorders*, *12*(1), 14. doi:10.1186/2251-6581-12-14

Singh, N., Armstrong, D. G., & Lipsky, B. A. (2005). Preventing Foot Ulcers in PatientsWith Diabetes. *American Medical Association*, *293*(2), 217–228.

Singh, S., Pai, D. R., & Yuhhui, C. (2013). Diabetic Foot Ulcer – Diagnosis and Management. *Clinical Research on Foot & Ankle*, *01*(03), 1–9. doi:10.4172/2329-910X.1000120

Sinor, M. Z. (2013). Oral Health Assessment among Elderly Staying in Shelter (Rumah Seri Kenangan), Kelantan , Malaysia. *International Journal of Humanities and Social Science Invention*, *2*(1), 43–48.

Tang, T. S., Funnell, M. M., Noorulla, S., Oh, M., & Brown, M. B. (2012). Sustaining short-term improvements over the long-term: Results from a 2-year diabetes self-management support (DSMS) intervention. *Diabetes Research and Clinical Practice*, *95*(1), 85–92. doi:10.1016/j.diabres.2011.04.003

Thomas, B., Ciliska, D., Dobbins, M., & Micucci, S. (2004). A process for systematically reviewing the literature: providing the reseach evidence for public health nursing interventions. *Worldviews on Evidence-Based Nursing*, *1*(January), 176–184.

Vileikyte, L., Peyrot, M., & Bundy, C. (2003). The Development and Validation of a Neuropathy- and Foot Ulcer – Specific Quality of Life Instrument. *Diabetes Care*, *26*(9), 2549–2555.

Visvanathan, R., Zaiton, a, Sherina, M. S., & Muhamad, Y. a. (2005). The nutritional status of 1081 elderly people residing in publicly funded shelter homes in Peninsular Malaysia. *European Journal of Clinical Nutrition*, *59*, 318–324. doi:10.1038/sj.ejcn.1602075

Wan Nazaimoon, W. M., Md Isa, S. H., Wan Mohamad, W. B., Khir, a S., Kamaruddin, N. a, Kamarul, I. M., … Khalid, B. a K. (2013). Prevalence of diabetes in Malaysia and usefulness of HbA1c as a diagnostic criterion. *Diabetic Medicine : A Journal of the British Diabetic Association*, *30*(7), 825–8. doi:10.1111/dme.12161

Wan-Ibrahim, W. A., Majid, M. A., & Zainab, I. (2014). Subjective Well-Being of Older Persons at Elderly Day-Care Center. *Middle-East Journal of Scientific Research*, *20*(11), 1461–1464. doi:10.5829/idosi.mejsr.2014.20.11.21081

Wan-Ibrahim W.A, & Zainab, I. (2014). Reasons Behind the Availability of Family Support of Older Persons in Malaysia. *Middle-East Journal of Scientific Research*, *20*(11), 1457–1460. doi:10.5829/idosi.mejsr.2014.20.11.21080

Waxman, R., Woodburn, H., Powell, M., Woodburn, J., Blackburn, S., & Helliwell, P. (2003). FOOTSTEP: A randomized controlled trial investigating the clinical and cost effectiveness of a patient self-management program for basic foot care in the elderly. *Journal of Clinical Epidemiology*, *56*, 1092–1099. doi:10.1016/S0895-4356(03)00197-5

Wilkinson, A., Whitehead, L., & Ritchie, L. (2014). Factors influencing the ability to self-manage diabetes for adults living with type 1 or 2 diabetes. *International Journal of Nursing Studies*, *51*(1), 111–22. doi:10.1016/j.ijnurstu.2013.01.006

Williams, I. C., Utz, S. W., Hinton, I., Yan, G., Jones, R., & Reid, K. (2014). Enhancing diabetes self-care among rural African Americans with diabetes: results of a two-year culturally tailored intervention. *The Diabetes Educator*, *40*(2), 231–239. doi:10.1177/0145721713520570

Wu, S. V., Courtney, M., Edwards, H., & Mcdowell, J. (2008). Psychometric Properties of the Chinese Version of the Perceived Therapeutic Efficacy Scale for Type 2 Diabetes. *Journal Formos Medical Association*, *107*(3), 232–238.

Wu, S. F., Courtney, M., Edwards, H., McDowell, J., Shortridge-Baggett, L. M. & Chang, J. R. (2007). Self-efficacy, outcome expectations and self-care behaviour in people with type 2 diabetes in Taiwan. *Journal compilation,* 250-257.

Appendix 1: Work plan

Work plan Effects of self-efficacy enhancing program on foot self-care behaviour of elderly with diabetes in Rumah Seri Kenangan, Peninsular Malaysia

**Gantt Chart**		Year 1												Year 2											
		2014				2015												2016							
		Sept	Oct	Nov	Dec	Jan	Feb	Mar	Apr	May	Jun	Jul	Aug	Sept	Oct	Nov	Dec	Jan	Feb	Mar	Apr	May	Jun	Jul	Aug
1	Literature review																								
2	Proposal development																								
3	Seminar proposal and ethical approval process																								
4	Pilot study / material preparation																								
5	Data collection / intervention process																								
6	Data analysis and statistical conclusion																								

Gantt Chart		Year 3											
		2016				2017							
		Sept	Oct	Nov	Dec	Jan	Feb	Mar	Apr	May	Jun	Jul	Aug
8	Discussion/ implication												
9	Documentation and dissemination												

Appendix 2: Respondent's information sheet and consent form

JAWATANKUASA ETIKA UNIVERSITI UNTUK PENYELIDIKAN MELIBATKAN MANUSIA (JKEUPM)

UNIVERSITI PUTRA MALAYSIA, [illegible] UPM SERDANG

FORM B1: RESPONDENT'S INFORMATION SHEET AND CONSENT

Please read the following information carefully and do not hesitate to discuss any questions you may have with the researcher.

1. STUDY TITLE :

Effects of Self-Efficacy Enhancing Program on Foot Self-Care Behaviour of Elderly with Diabetes in Rumah Seri Kenangan, Peninsular Malaysia

2. INTRODUCTION:

Diabetes foot complications give a great impact on elderly patients. Foot self-care behaviour is an essential management in preventing foot complications of diabetes.

3. WHAT WILL YOU HAVE TO DO?

You will be assessed for blood glucose, mental health, cognitive and depression level. If you have an abnormal result, we will inform to the health care provider for further action. During the program, you will receive a diabetes education about foot self-care or receive a usual health care.

4. WHO SHOULD NOT PARTICIPATE IN THE STUDY?:

Elderly who has mental health problem, cognitive impairment, depression and not able to do self-care such as bathing, grooming and walking independently.

5. WHAT WILL BE THE BENEFITS OF THE STUDY:

(a) TO YOU AS THE SUBJECT?

The study may increase your knowledge to perform diabetes foot self-care or you may receive a usual health care.

(b) TO THE INVESTIGATOR?

The aim after completed the program, the result can be used to improve diabetes education and health outcome of elderly with diabetes.

6. WHAT ARE THE POSSIBLE RISKS?

We need to prick your finger to obtain a drop of blood for glucose test. You may feel pain after the procedure.

7. WILL THE INFORMATION THAT YOU PROVIDE AND YOUR IDENTITY REMAIN CONFIDENTIAL?

All information will be kept confidential

8. WHO SHOULD YOU CONTACT IF YOU HAVE ADDITIONAL QUESTIONS DURING THE COURSE OF THE RESEARCH?

If you have any question, please contact Associate Professor Dr Hejar Abdul Rahman (03-89472417).

Please initial here if you have read and understood the contents of this page ______________

9. CONSENT

I …………………………………… Identity Card No. …………………………… address………………………………………………………………………………………………… …... …………………………………………………..hereby voluntarily agree to take part in the research stated above *(clinical /drug trial/video recording/ focus group/interview-based/ questionnaire-based). I have been informed about the nature of the research in terms of methodology, possible adverse effects and complications (as written in the Respondent's Information Sheet). I understand that I have the right to withdraw from this research at any time without giving any reason whatsoever. I also understand that this study is confidential and all information provided with regard to my identity will remain private and confidential.

I* wish / do not wish to know the results related to my participation in the research

I agree/do not agree that the images/photos/video recordings/voice recordings related to me be used in any form of publication or presentation (if applicable)

* delete where necessary

Signature …….………………………… Signature …….…………………………

(Respondent) (Witness)

Date :…………………………………….. Name :……………………………………..

I/C No. :……………………………………..

I confirm that I have explained to the respondent the nature and purpose of the above-mentioned research.

Date …….………………………… Signature …….…………………………

(Researcher)

Appendix 3: Questionnaire

Doctor of Philosophy Program
Faculty of Medicine and Health sciences
Universiti Putra Malaysia
Serdang, Selangor
2014/2015

Dear Sir/ Madam,
My name is Siti Khuzaimah Ahmad Sharoni and I am a postgraduate doing my Doctor of Philosophy (PhD) of Community Health Department at Universiti Putra Malaysia, Serdang. As part of the fulfilment of the requirement of the program, I have to complete a thesis. The topic of my thesis is "effects of self-efficacy enhancing program on foot self-care behaviour of elderly with diabetes in Rumah Seri Kenangan, Peninsular Malaysia".

Herewith, I would appreciate for your cooperation in answering my questionnaire. For your information, your name and identity will not be exposed in this study. For legal purposes, all data will be kept confidential and will be used only for this study.

I would be grateful if you would kindly answer the questionnaires and return it to me as soon as you finish it or return to the respective in charge as soon as possible. Lastly, thank you for your co-operation.

The effects of self-efficacy enhancing program on foot self-care behaviour of elderly with diabetes in Rumah Seri Kenangan, Peninsular Malaysia

Instructions:

Please answer all the questions by placing a clear ☑ sign in the space provided or following the given instructions. The questionnaire will take approximately 20 minutes to complete.

The questionnaire is divided into the following sections:

Part A: Demographic data
Part B: Clinical characteristics
Part C: Foot self-care behaviour
Part D: Foot care self-efficacy (expectation)
Part E: Foot care outcome expectation
Part F: Knowledge on foot care
Part G: Quality of life

Please return the completed questionnaire to the respective in charge.
Your contribution to this research study is much appreciated

For the researcher only	
Date : _______________ Setting : RSK ___________ ID : _______________	FBS : _______________ Katz score : _______________ ECAQ score : _______________ GDS score : _______________

Part A: Demographic data

Please **choose** the appropriate answer to each question and tick (√) in the box provided and give answers where specified.

1. Age : _____ years ______ months

2. Gender : ( )1 Male ( )2 Female

3. Ethnic : ( )1 Malay ( )2 Chinese
   ( )3 Indian ( )4 Others

4. Education level : ( )1 Never ( )2 Primary
   ( )3 Secondary ( )4 Tertiary

5. Marital status : ( )1 Never ( )2 Married
   ( )3 Widowed ( )4 Separated

6. No of children : ___________

7. Duration stayed at this RSK: ___________ years _________ months

Part B: Clinical characteristics

Please **choose** the appropriate answer to each question and tick (√) in the box provided and give answers where specified. You may tick (√) more than one answer

1. Duration of diabetes : __________ years _________ months

2. Treatment of diabetes : ( )1 Diet control ( )2 Oral medication
   ( )3 Insulin ( )4 Others, _____________

3. Other disease (except diabetes) : ( )1No
   ( )2Yes, _______________

4. Received diabetes education : ( )1No or
   if yes, the education is about _________________, when: __________ from: nurse/ doctor/ or _____________
5. Current smoking : ( )1No ( )2Yes, _______________
6. Recent hospitalization : ( )1No ( )2Yes, _______________

Part C: Foot self-care behaviour

Section 1: The questions below ask you about your diabetes self-care activities during the past 7 days. If you were sick during the past 7 days, please think back to the last 7 days that you were not sick. Please circle in an appropriate answer.

	**The activities during the past 7 days**							
1. I examine my feet	0	1	2	3	4	5	6	7
2. I examine the bottoms of my feet	0	1	2	3	4	5	6	7
3. I examine between the toes of my feet	0	1	2	3	4	5	6	7
4. I wash my feet	0	1	2	3	4	5	6	7
5. I wash between my toes	0	1	2	3	4	5	6	7
6. I dry my feet after washing it	0	1	2	3	4	5	6	7
7. I dry between my toes after washing my feet.	0	1	2	3	4	5	6	7

Section 2: Below is a list of activities your may have to perform to manage your feet. Please read each one and then circle the category which best reflects what you actually do. For example, if you are always carry out that you are able to protect your feet, circle 7. If you feel that sometimes you could do it, circle 4 or 3. If you feel that all of the time you never do it, circle 0.

	**Never**			**sometimes**				**Always**
8. I trim my toenails straight across.	0	1	2	3	4	5	6	7
9. I apply moisturizing lotion to my feet.	0	1	2	3	4	5	6	7
10. I wear cotton and loose socks every time I walk (includes walking indoors).	0	1	2	3	4	5	6	7
11. I wear suitable (wide) shoes/ sturdy slippers every time I walk (includes walking indoors).	0	1	2	3	4	5	6	7
12. Before I put on my shoes/ sturdy slippers, I check the inside of my shoes/ sturdy slippers.	0	1	2	3	4	5	6	7
13. I break in new shoes/ sturdy slippers slowly	0	1	2	3	4	5	6	7
14. I choose suitable and correct shoes/ sturdy slippers for my feet.	0	1	2	3	4	5	6	7
15. I tell my doctor/ nurse/ medical assistant (if any) about problems with my feet	0	1	2	3	4	5	6	7
16. I recognize when my toenails need to be trimmed by a doctor/ nurse/ medical assistant	0	1	2	3	4	5	6	7

Part D: Foot care self-efficacy (efficacy expectation)

After reading each statement, please tick (√) under the description that best describes how CONFIDENT you are about undertaking yourself each of the following foot-care tasks. Please answer **about your CONFIDENCE to do the foot care, not about what you actually do.**

	Strongly not confident			Strongly confident	
1. I can protect my feet					
2. Even without pain/discomfort, I can look at my feet daily to check for cuts, scratches, blisters, redness or dryness.					
3. After washing my feet, I can dry between my toes.					
4. I can judge when my toenails need to be trimmed by a podiatrist					
5. I can trim my toenails straight across					
6. I can figure out when to use a pumice stone to smooth corns and/or calluses on my feet.					
7. I can test the temperature of the water before putting my feet into it.					
8. If I was told to do so, I can wear shoes and socks every time I walk (includes walking indoor.					
9. When I go shopping for new shoes, I can choose shoes that are good for my feet.					
10. I can call my doctor about problems with my feet .					
11. Before putting them on, I can check the insides of my shoes for problems that could harm my feet.					
12. If I directed to do so, I can routinely apply lotion to my feet.					

Part E: Foot care outcome expectation

After reading each statement, please tick (√) under the description that best describes how CONFIDENT you are about undertaking yourself each of the following foot-care tasks. Please answer **about your CONFIDENCE to do the foot care, not about what you actually do.**

When I perform good foot self-care behaviour, I become more confident that I can	**Strongly disagree**				**Strongly agree**
1. protect my feet from injury	1	2	3	4	5
2. look at my feet daily to check for cuts, scratches, blisters, redness or dryness	1	2	3	4	5
3. keep hygiene of my foot/ feet	1	2	3	4	5
4. keep my toes always dry	1	2	3	4	5
5. Keep my feet/ foot not dry (by putting lotion when my skin is dry)	1	2	3	4	5
6. judge when my foot needs appropriate treatment by a doctor/ nurse/ medical assistant	1	2	3	4	5

Part F: Knowledge on foot care

The questions below ask about your knowledge on foot care. Please **circle** in an appropriate answer.

People with diabetes should…….	**Answer**			**Remark**
1. take medication regularly because they liable to get diabetes complication	True	False	Don't know	
1. look after their feet because they are more liable to get infection (true)	True	False	Don't know	
2. look after their feet because they may not feel a minor injury to their feet (true)	True	False	Don't know	
3. look after their feet because wounds and infection may not heal quickly (true)	True	False	Don't know	
4. look after their feet because they may get a foot ulcer (true)	True	False	Don't know	
5. not smoke because smoking causes poor circulation affecting the feet (true)	True	False	Don't know	
6. check their feet daily (true)	True	False	Don't know	
7. treat themselves if they found redness/ bleeding between their toes (false).	True	False	Don't know	
8. Tell doctor if they have corn/ hard skin lesion	True	False	Don't know	
9. wash their feet daily (true)	True	False	Don't know	
10. check temperature of the water before wash their feet to avoid injury (true)	True	False	Don't know	
11. check inside of their shoes only when necessary (false)	True	False	Don't know	

Part G: Quality of life

These questions ask about the effect your **FOOT PROBLEMS** may have on your daily life and well-being. By **foot problems** we mean **lost or reduced feeling** in your extremities**, pain, discomfort** and/or **ulcers (open sores) on your feet** and, in some cases **unsteadiness** while walking or standing. Please note that many **questions have three parts**. Answer every question by filling in the blank or ticking one box for each part tick (√) two boxes per line). Please make sure you answer all questions. Please concentrate on how you have felt **IN THE PAST 4 WEEKS** for all of the questions.

**In the past 4 weeks how often have you experienced the following symptoms?**	All the time	Some time	Never	**How much bother did this cause you?**		
				Very much	Some bother	None
1. Burning in your legs or feet						
2. Excessive heat or cold in your legs or feet						
3. Pins and needles in your legs or feet						
4. Shooting or stabbing pain in your legs or feet						
5. Throbbing in your legs or feet						
6. Sensations in your legs or feet that make them jump						
7. Irritation of the skin caused by something touching your feet, such as bedsheets or socks						

**In the past 4 weeks how often have you experienced the**	All the time	Some time	Never	**How much bother did this cause you?**

following symptoms?				Very much	Some bother	None
8. Numbness in your feet						
9. Inability to feel the difference between hot and cold with your feet						
10. Inability to feel objects with your feet						

**In the past 4 weeks how often have you experienced the following symptoms?**	All the time	Some time	Never	**How much bother did this cause you?**		
				Very much	Some bother	None
11. Weakness in your feet						
12.Problems with balance or unsteadiness while walking						
13.Problems with balance or unsteadiness while standing						

The following questions ask about how your FOOT PROBLEMS affect your daily activities, relationships and feelings.

Are you in PAID WORK?	Yes	No	If **YES** please go to Question 14.<br>If **NO** please go to Question 15.

**In the past 4 weeks, HOW MUCH have your foot problems interfered with your:**	Very much	Some what	Not at all	**How important is this aspect of your life to you?**		
				Very much	Some what	Not at all
14. Ability to perform your paid work?						
15. Ability to perform tasks around the house or garden?						
16. Ability to take part in leisure activities?						

**In the past 4 weeks:**				**How important is this aspect of your life to you?**		
	Very much	Some what	Not at all	Very much	Some what	Not at all
17. How much has your foot problems interfered with your relationships with people close to you?						
18. Have you felt more physically dependent than you would like to be on people close to you as a result of your foot problems?						
19. Have you felt more emotionally dependent than you would like to be on people close to you as a result of your foot problems?						
20. Has your role in the family changed as a result of your foot problems?						

	**How much bother did this cause you?**

How much do you agree with the following statements:	Agree	Neither agree or disagree	Disagree	Very much	Some bother	None
21. People treat me differently from other people as a result of my foot problems.						
22. I feel older than my years as a result of my foot problems.						
23. My self - confidence is affected as a result of my foot problems.						
24. My foot problems make my life a struggle.						
25. I generally feel frustrated because of my foot problems.						
26. My foot problems cause me embarrassment.						
27. I feel depressed because of my foot problems						

Appendix 4: Permission (instrument used) from original author

Permission to use the instrument was granted from the original researcher
The Foot Self-Care Behaviour Scale

• Re: Development and Validation of a Diabetes Foot Self-Care Behavior Scale People ★

Siti Khuzaimah Ahmad Sharoni
No of student: GS40555 (Universiti Putra Malaysia)

On Sunday, September 28, 2014 8:50 AM, "yenfan@mail.cgu.edu.tw" <yenfan@mail.cgu.edu.tw> wrote:

Dear Siti Khuzaimah
We are glad to know that you are interested to the Diabetes Foot Self-Care Behavior Scale (DFSBS). Dr. Huang and I both agree to let you use the DFSBS in your program. The attachment is the instruction of the DFSBS and the DFSBS. Please let me you if you have any question about the DFSBS.
Sincerely yours
Tzu-Ting Huang and Yen-fan Chin

寄件人: "Siti Khuzaimah" <sitik123@yahoo.com>
收件人: yenfan@mail.cgu.edu.tw
寄件箱: 2014年9 月27日, 星期六 下午 7:15:49

The Quality of Life Scale

• RE: The development and validation of a neuropathy- and foot ulcer-specific quality of lif... People ★

Vileikyte, Loretta Sep 29
To me

Please find attached.
Loretta Vileikyte, MD, PhD
Senior Lecturer in Medicine
School of Clinical and Laboratory Sciences,
University of Manchester, Manchester, UK
phone: +44 (0) 161 276 8953
fax: +44 (0) 161 274 4740
email: lvileikyte@med.miami.edu

From: Siti Khuzaimah [sitik123@yahoo.com]
Sent: 26 September 2014 23:23
To: Vileikyte, Loretta
Subject: The development and validation of a neuropathy- and foot ulcer-specific quality of life instrument

Dear Dr Loretta Vileikyte,

Thank you for replying my email.

After further reading, I am also interested with the instrument of neuropathy and foot ulcer specific QOL. Here, I am seeking your permission to adopt/adapt your both scale into my study.

Knowledge of diabetic foot care

• Re: DIABETIC FOOT CARE: SELF REPORTED KNOWLEDGE AND PRACTICE AMONG PATIE... People

desalu olufemi <femuy1967@yahoo.co.uk> 02/19/15 at 8:35 PM
To Siti Khuzaimah

Doc,
Permission is granted with pleasure.
The questionnaire is attached
Thanks
Dr Olufemi .O Desalu MB,Ch.B FMCP(Nig)
Senior Lecturer /Consultant Physician & Pulmonologist
University of Ilorin /University Ilorin Teaching Hospital
Division of Pulmonary Medicine
Department of Medicine
P.M.B 1459, Ilorin
Kwara State, Nigeria 240001
MobileTel:+2348035025771
http://www.unilorin.edu.ng/

On Wednesday, 4 February 2015, 3:01, Siti Khuzaimah <sitik123@yahoo.com> wrote:

Appendix 5: Instrument for screening

Katz Index of Independence in Activities of Daily Living

**ACTIVITIES**<br>POINTS (1 OR 0)	**INDEPENDENCE:**<br>(1 POINT)<br>**NO** supervision, direction or personal assistance	**DEPENDENCE:**<br>(0 POINTS)<br>**WITH** supervision, direction, personal assistance or total care
**BATHING**<br>POINTS:___________	**(1 POINT)** Bathes self completely or needs help in bathing only a single part of the body such as the back, genital area or disabled extremity.	**(0 POINTS)** Needs help with bathing more than one part of the body, getting in or out of the tub or shower. Requires total bathing.
**DRESSING**<br>POINTS:___________	**(1 POINT)** Gets clothes from closets and drawers and puts on clothes and outer garments complete with fasteners. May have help tying shoes.	**(0 POINTS)** Needs help with dressing self or needs to be completely dressed.
**TOILETING**<br>POINTS:___________	**(1 POINT)** Goes to toilet, gets on and off, arranges clothes, cleans genital area without help.	**(0 POINTS)** Needs help transferring to the toilet, cleaning self or uses bedpan or commode.
**TRANSFERRING**<br>POINTS:___________	**(1 POINT)** Moves in and out of bed or chair unassisted. Mechanical transferring aides are acceptable.	**(0 POINTS)** Needs help in moving from bed to chair or requires a complete transfer.
**CONTINENCE**<br>POINTS:___________	**(1 POINT)** Exercises complete self control over urination and defecation.	**(0 POINTS)** Is partially or totally incontinent of bowel or bladder.
**FEEDING**<br>POINTS:___________	**(1 POINT)** Gets food from plate into mouth without help. Preparation of food may be done by another person.	**(0 POINTS)** Needs partial or total help with feeding or requires parenteral feeding.

***ELDERLY COGNITIVE ASSESSMENT QUESTIONNAIRE* (*ECAQ*)**

No.	ECAQ	Satu Markah Untuk Jawapan Yang Betul
	**MEMORI**	
1.	Saya mahu anda ingatkan nombor ini. Tolong ulang selepas saya sebutkannya (eg. 3517).<br>Saya akan memeriksa anda lagi dalam masa 10 minit	
2.	Berapakah umur anda?	
3.	Bilakah harijadi anda?<br>(Atau dalam tahun berapa anda dilahirkan?)	
	**ORIENTASI-INFORMASI**	
4.	Apakah hari ini?	
5.	Apakah tarikh hari ini?	
6.	Apakah bulan ini?	
7.	Apakah tahun ini?	
8.	Apakah nama tempat ini (contoh klinik, hospital)?	
9.	Apakah pekerjaan dia (contoh doktor, jururawat)?	
	**MEMORI - INGATAN SEMULA**	
10.	Dapatkah anda ingat nombor tadi semula?	
	**JUMLAH**	

***GERIATRIC DEPRESSION SCALE* (Bahasa Malaysia version)**

Dalam masa sebulan yang lepas:

No.	Soalan	Skor	
		**Ya**	**Tidak**
1.	Adakah anda berpuashati dengan kehidupan anda sekarang?	0	1
2.	Adakah anda merasa hidup anda kosong?	1	0
3.	Adakah anda takut akan sesuatu yang buruk akan menimpa anda?	1	0
4.	Adakah anda merasa gembira setiap masa?	0	1
	**JUMLAH**		

Appendix 6: Budget

**A. BELANJAWAN** *BUDGET*			
**Sila anggarkan belanjawan untuk penyelidikan ini dan butiran perbelanjaan dengan merujuk kepada garis panduan yang telah dilampirkan.**<br>*Please indicate the estimated budget for the research and details of expenditure according to the guidelines attached.*			
**Butiran belanjawan**<br>*Budget details*	**Jumlah yang diminta oleh pemohon**<br>*Amount requested by applicant*		
	**Tahun 1**<br>*Year 1*	**Tahun 2**<br>*Year 2*	**Tahun 3**<br>*Year 3*
1. ***Vote 11000***<br>Gaji dan upahan<br>(Tidak terpakai untuk Geran Putra-IPM dan Putra-IPS*)*<br>*Salary and wages*<br>*(Not applicable for Geran Putra-IPM and Putra-IPS)*	**NA**	**NA**	**NA**
2. **Vote 21000**<br>Perbelanjaan perjalanan dan pengangkutan<br>*Travel expenses and transportation*<br><br>To and fro = Faculty/ Home – study setting (Rumah Seri Kenangan, Peninsular Malaysia)		RM 1000.00	RM 500.00
3. **Vote 24000**<br>Sewaan *Rental*	**NA**	**NA**	**NA**
4. **Vote 26000** Bekalan bahan penyelidikan serta bekalan bahan mentah dan bahan-bahan untuk penyenggaraan dan pembaikan *termasuk vot 27000.<br>*Research materials and materials for repair and maintenance*including vote 27000*<br>**Research Materials & Supplies**<br>Blood glucose meter<br>Glucose strip<br>Alcohol cotton swab<br>Dry cotton swab<br>Needle<br>Gloves<br>Sharp Bin		RM 1000.00	
5. **Vote 28000**<br>Penyelenggaraan dan pembaikan kecil<br>*Maintenance and minor repair services*	**NA**	**NA**	**NA**
6. **Vote 29000**<br>Perkhidmatan profesional, hospitaliti dan lain-lain<br>*Professional services, hospitality, and other services*<br><br>**Professional services**<br>Translation/ consultation/ proof reading/ kursus jangka pendek		RM 2000.00	RM 1350.00

**Honorarium**<br>Study setting = RM100.00 x 8 RSK<br>Respondents = RM15.00 x 250 people<br>Research assistant = RM150.00 x 8 RSK<br><br>**Printing / photocopy**<br>Reading materials<br>RM2.00 x 250 (respondents and health provider)<br>Questionnaire (pilot study)<br>RM2.00 x 50 respondents = RM100 (x3 series of visit)<br>Questionnaire (real study)<br>RM2.00 x 250 respondents = RM500.00 (x3 series of visit)<br><br>**Stationeries**<br>Files, pen, pencil, A4 paper, envelope, staple, bullet, punch hole, eraser, liquid paper, CD		RM 500.00<br>RM 300.00<br>RM 1500.00<br>RM 700.00	RM 800.00<br>RM 3750.00<br>RM 1600.00
7. **Vote 35000** Peralatan dan aksesori<br>*Equipment and accessories*<br>***Sila rujuk garispanduan untuk keterangan lanjut***<br>***(Please refer to guidelines for more information)***	**NA**	**NA**	**NA**
**Bajet tahunan yang dicadangkan**<br>*Proposed yearly budget*		**RM 7000.00**	**RM 8000.00**
**JUMLAH KESELURUHAN**<br>*TOTAL AMOUNT*	**RM 15000.00**		
