# Supplementary material for: The effects of self-efficacy enhancing program on foot self-care behaviour of older adults with diabetes: A randomised controlled trial in elderly care facility, Peninsular Malaysia
Source: PLoS One. 2018 Mar 13;13(3):e0192417. doi: 10.1371/journal.pone.0192417 (PMC5849313; doi:10.1371/journal.pone.0192417)
Supplement: S4 File — (PDF) [file pone.0192417.s004.pdf]

## ETHICS COMMITTEE FOR RESEARCH INVOLVING HUMAN SUBJECTS (JKEUPM) UNIVERSITI PUTRA MALAYSIA

| | | |
|---|---|---|
| **Research title** | : | **Effects of Self-Efficacy Enhancing Program on Foot Self-Care Behaviour of Elderly with Diabetes in Rumah Seri Kenangan, Peninsular Malaysia** |
| **Study Site** | : | **Peninsular Malaysia** |
| **JKEUPM Ref No.** | : | **FPSK(FR15)P021** |
| **Researcher** | : | **Siti Khuzaimah Ahmad Sharoni** |
| **Supervisor** | : | **Assoc Prof Dr Hejar Abdul Rahman** |

Documents received and reviewed with reference to the above study:

1. Ethics Application Form, received on 2/7/2015.
2. Respondent Information Sheet & Consent, English Version.
3. Respondent Information Sheet & Parent's/Guardian's Consent, English Version.
4. Proposal, English Version.
5. Questionnaire, English and Malay Version.

The University Research Ethics Committee, Universiti Putra Malaysia (JKEUPM) operates in accordance to the ICH-GCP Guidelines.

Decision by JKEUPM:

[X] Approved

[ ] Permission must be obtained from the respective hospitals/ institutions before conducting the research

[ ] Disapproved

Please be informed that you are required to submit annual progress reports, completion reports and "all adverse events, both serious and unexpected" to the committee. If amendments to the original protocol are inevitable, prior approval must be obtained from the committee.

Date of Decision: 10/8/2015

**PROFESSOR DR. ABDUL JALIL NORDIN**
Chairperson,
Ethics Committee for Research involving
Human Subjects (JKEUPM),
Universiti Putra Malaysia

UPM
UNIVERSITI PUTRA MALAYSIA
BERILMU BERBAKTI

QUALITY SYSTEM
SIRIM
UKAS MANAGEMENT SYSTEMS
CERTIFIED TO ISO 9001:2008
CERT. NO. : AR2020

PEJABAT TIMBALAN NAIB CANSELOR (PENYELIDIKAN DAN INOVASI)
*OFFICE OF THE DEPUTY VICE CHANCELLOR (RESEARCH AND INNOVATION)*

Reff. : UPM/TNCPI/RMC/1.4.18.1 (JKEUPM)/F2
Date : 10th August 2015

Assoc Prof Dr Hejar
Department of Community Health
Faculty of Medicine and Health Sciences
Universiti Putra Malaysia
Serdang, Selangor

Dear Madam,

**RESEARCH PROJECT: EFFECT OF SELF-EFFICACY ENHANCING PROGRAM ON FOOT SELF-CARE BEHAVIOUR OF ELDERLY WITH DIABETES IN RUMAH SERI KENANGAN PENINSULAR MALAYSIA**
**PROJECT REF. NO: FPSK (FR15)P021**

**RESEARCHER : SITI KHUZAIMAH AHMAD SHARONI**
**SUPERVISOR : ASSOC PROF DR HEJAR ABDUL RAHMAN**

The Ethics Committee for Research involving Human Subjects of University Putra Malaysia (JKEUPM) has studied the proposal for the above project and found that there were no objectionable ethical issues involved in the proposed study.

Please find the list of documents received and reviewed with reference to the study and committee members who reviewed the documents (as attached).

Notwithstanding above, we will not be responsible for any misconduct on the part of researcher in the course of carrying out the research.

Thank you.

**"WITH KNOWLEDGE WE SERVE"**

Sincerely yours,

**PROFESSOR DR. ABDUL JALIL NORDIN**
Chairperson
Ethics Committee for Research involving Human Subjects (JKEUPM)
Universiti Putra Malaysia
