# Supplementary material for: The effects of self-efficacy enhancing program on foot self-care behaviour of older adults with diabetes: A randomised controlled trial in elderly care facility, Peninsular Malaysia
Source: PLoS One. 2018 Mar 13;13(3):e0192417. doi: 10.1371/journal.pone.0192417 (PMC5849313; doi:10.1371/journal.pone.0192417)
Supplement: S5 File — (PDF) [file pone.0192417.s005.pdf]

JABATAN KEBAJIKAN MASYARAKAT
Department of Social Welfare
Aras 6, 9-18, No. 55 Persiaran Perdana,
Presint 4,
62100 PUTRAJAYA.

Berkat Berjasa

Tel : 603 - 8323 1000
Faks *(Fax)* : 603 - 8323 2045
Laman Web *(Website)* : www.jkm.gov.my

JKMM 100/12/5/2 : 2015 / 003

12 November 2015

SITI KHUZAIMAH AHMAD SHARONI
NO 6, JALAN SP 5/5, SERI PRISTANA
SUNGAI BULOH
47000
SELANGOR

Sir/Madam,

APPROVAL FOR CONDUCTING RESEARCH AT THE SOCIAL WELFARE DEPARTMENT

I refer to the above,

Please be informed that the application sir / madam to conduct studies / research entitled Effects of Self-Efficacy Enhancing Program on Foot Self-Care Behaviour of Elderly with Diabetes in Rumah Seri Kenangan, Peninsular Malaysia and study/ research in the Appendix was approved. The approval period for carry out studies/ research in the chosen place is for TWELVE (12) month starting from 12 November 2015 until 12 November 2016.

Accordingly, sir / madam required to submit two (2) copies thesis / reports / publications and bound to the Department before or at the latest on February 12, 2017. For further information, sir / madam can contact the Planning and Development, Social Welfare Department at 03-83231935 or online mnadzri@jkm.gov.my mail.

Thank you.

"SERVICE TO THE NATION"

Yours faithfully,

DR. ZAITOL BINTI SALLEH
Planning and Development Division
i.p. Director General of Social Welfare, Malaysia
c.c Deputy Director (Strategic) of Social Welfare

ATTACHMENT

Reference : JKMM 100/12/5/2 : 2015 / 003

Date : 12 November 2015

Study title : Effects of Self-Efficacy Enhancing Program on Foot Self-Care Behaviour of Elderly with Diabetes in Rumah Seri Kenangan, Peninsular Malaysia

Address of study setting :

1) RUMAH SERI KENANGAN CHERAS

2) RUMAH SERI KENANGAN TAMAN KEMUMIN

3) RUMAH SERI KENANGAN KANGAR

4) RUMAH SERI KENANGAN BEDONG

5) RUMAH SERI KENANGAN TAIPING

6) RUMAH SERI KENANGAN TANJUNG RAMBUTAN

7) RUMAH SERI KENANGAN CHENG

8) RUMAH SERI KENANGAN JOHOR BAHRU
