# Supplementary material for: The effects of self-efficacy enhancing program on foot self-care behaviour of older adults with diabetes: A randomised controlled trial in elderly care facility, Peninsular Malaysia
Source: PLoS One. 2018 Mar 13;13(3):e0192417. doi: 10.1371/journal.pone.0192417 (PMC5849313; doi:10.1371/journal.pone.0192417)
Supplement: S6 File — (PDF) [file pone.0192417.s006.pdf]

JAWATANKUASA ETIKA UNIVERSITI UNTUK PENYELIDIKAN MELIBATKAN MANUSIA (JKEUPM)
UNIVERSITI PUTRA MALAYSIA, 43400 UPM SERDANG, SELANGOR, MALAYSIA

FORM B1: RESPONDENT'S INFORMATION SHEET AND CONSENT

Please read the following information carefully and do not hesitate to discuss any questions you may have with the researcher.

1. STUDY TITLE :
Effects of Self-Efficacy Enhancing Program on Foot Self-Care Behaviour of Elderly with Diabetes in Rumah Seri Kenangan, Peninsular Malaysia

2. INTRODUCTION:
Diabetes foot complications give a great impact on elderly patients. Foot self-care behaviour is an essential management in preventing foot complications of diabetes.

3. WHAT WILL YOU HAVE TO DO?
You will be assessed for blood glucose, mental health, cognitive and depression level. If you have an abnormal result, we will inform to the health care provider for further action. During the program, you will receive a diabetes education about foot self-care or receive a usual health care.

4. WHO SHOULD NOT PARTICIPATE IN THE STUDY?:
Elderly who has mental health problem, cognitive impairment, depression and not able to do self-care such as bathing, grooming and walking independently.

5. WHAT WILL BE THE BENEFITS OF THE STUDY:

(a) TO YOU AS THE SUBJECT?
The study may increase your knowledge to perform diabetes foot self-care or you may receive a usual health care.

(b) TO THE INVESTIGATOR?
The aim after completed the program, the result can be used to improve diabetes education and health outcome of elderly with diabetes.

6. WHAT ARE THE POSSIBLE RISKS?
We need to prick your finger to obtain a drop of blood for glucose test. You may feel a finger pain after the procedure.

7. WILL THE INFORMATION THAT YOU PROVIDE AND YOUR IDENTITY REMAIN CONFIDENTIAL?
All information will be kept confidential

8. WHO SHOULD YOU CONTACT IF YOU HAVE ADDITIONAL QUESTIONS DURING THE COURSE OF THE RESEARCH?
If you have any question, please contact Associate Professor Dr Hejar Abdul Rahman (03-89472417).

Please initial here if you have read and understood the contents of this page ______________

9. CONSENT

I ………………………………… Identity Card No. …………………………… address…………………………………………………………………………………………………...
…………………………………………………..hereby voluntarily agree to take part in the research stated above *(clinical /drug trial/video recording/ focus group/interview-based/ questionnaire-based).

I have been informed about the nature of the research in terms of methodology, possible adverse effects and complications (as written in the Respondent's Information Sheet). I understand that I have the right to withdraw from this research at any time without giving any reason whatsoever. I also understand that this study is confidential and all information provided with regard to my identity will remain private and confidential.

I* wish / do not wish to know the results related to my participation in the research

I agree/do not agree that the images/photos/video recordings/voice recordings related to me be used in any form of publication or presentation (if applicable)

* delete where necessary

Signature ……..………………………… (Respondent)

Signature ……..………………………… (Witness)

Date :……………………………………..…..

Name :……………………………………..…..

I/C No. :……………………………………..…..

I confirm that I have explained to the respondent the nature and purpose of the above-mentioned research.

Date ……..…………………………

Signature ……..………………………… (Researcher)
